# Coordinative Behavior of a New Hydroxynaphthanyl Sulphonamide Tridentate Schiff Base Towards First Row Late Transition Metal (LTM) and Post-Transitional Metal Atoms Zn and Cd: A Crystallographic and Computational Study

**DOI:** 10.3390/molecules30173543

**Published:** 2025-08-29

**Authors:** Laura Sánchez-Guirao, Joaquín Viqueira, Carlos Silva López, José A. García-Vázquez, Jesús Castro

**Affiliations:** 1 Departamento de Química Orgánica, Universidad de Vigo, 36310 Pontevedra, Galicia, Spain; lasanchez@uvigo.gal; 2 Departamento de Química Inorgánica, Universidad de Santiago de Compostela, 15782 Santiago de Compostela, Galicia, Spain; joaquinangel.viqueira@gmail.com (J.V.); josearturo.garcia@usc.es (J.A.G.-V.); 3 Departamento de Química Inorgánica, Universidad de Vigo, 36310 Pontevedra, Galicia, Spain

**Keywords:** Schiff base ligands, electrochemical synthesis, X-ray structures, density functional calculations

## Abstract

The electrochemical oxidation of anodic metal (cobalt, nickel, zinc or cadmium) in a cell containing an acetonitrile solution of the ligand (E)-N-(2-(((2-hydroxynaphthalen-1-yl)methylene)amino)phenyl)-4-methylbenzenesulphonamide (H_2_L) affords complexes with the general formula [ML] (M = Co, Ni, Zn and Cd). Additionally, it was possible to obtain complexes with the general formula [MLL′] when L′ = 2,2-bipyridine (2,2-bpy), 4,4-bipyridine (4-4′-bpy) or 1,10-phenanthroline (phen) was present in the electrolytic cell. All of the compounds obtained have been characterized via microanalysis, IR spectroscopy, mass spectrometry, UV–visible spectroscopy and, in the case of diamagnetic compounds, via ^1^H NMR spectroscopy. Further structural and electronic characteristics of these adducts have been obtained via DFT simulations. The compounds NEt_4_[CoL_2_] (**1**), [NiL(H_2_O)] (**2**), [NiL(CH_3_CN)(H_2_O)]_2_ (**3**), [Ni_2_L_2_(4,4′-bpy)] (**4**), [Zn_2_L_2_(MeOH)_2_] (**5**) and [ZnL(2,2′-bpy)](CH_3_CN) (**6**) have been characterized via X-ray diffraction. In this paper, we present a detailed study of the different behavior of the above-mentioned ligand depending on the metal and/or the presence of ancillary ligands.

## 1. Introduction

Metal complexes with Schiff base ligands have played an important role from the early days of coordination chemistry, when they were described by Ugo Schiff [1]. In fact, a great deal of work has been carried out on the synthesis and characterization of transition metal compounds with these ligands, not only from an inorganic point of view but also due to their applications in several fields; as a result, a number of reviews were published in last several years [2,3,4,5,6,7,8,9]. Bbiological activity of Schiff base complexes as anticancer, antiviral or antimicrobial agents has probably been one of the most studied fields [10,11,12,13,14], but other properties of these compounds have also been explored, such as catalytic applications [15,16,17,18,19,20], electronic materials and luminescent compounds [21,22,23].

Schiff base ligands usually include other donor atoms within the molecule in order to obtain chelating abilities which provide stability to the complexes. Sulphonamide derivatives incorporate such an additional donor atom, an amide nitrogen atom susceptible to deprotonation. The combination of the Schiff base imine and the sulphonamide donor atom allows the formation of stable chelate rings with the metal ion [24]. The incorporation of an additional phenol group, in this case belonging to a 2-hydroxynaphtalenyl moiety, allows for the formation of a versatile potentially tridentated NNO ligand [25]. Cu(II) complexes with these or similar ligands were previously studied by our group due to their anticancer activity [26,27]. It is also worth noting that Ni(II) and Co(II) complexes of similar ligands have demonstrated biological activity as fungistatic, antiprotozoal or antibacterial agents [28], whereas Co(II) and Zn(II) complexes have shown antiproliferative activity [29]. Other closely related complexes have been described to have a cytotoxic effect [30].

**Figure 1:**
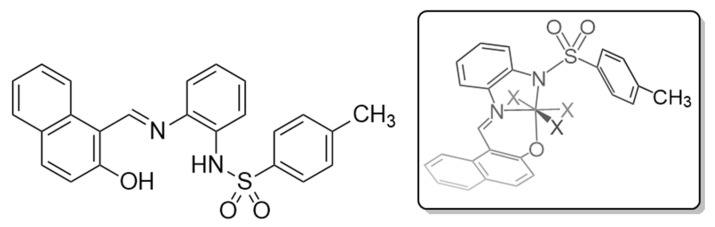
H_2_L. The inset represents the expected coordination mode after double deprotonation in the electrolytic cell.

For these reasons, and as a result of our continuing interest in the chemistry of Schiff base metal complexes, we now report the synthesis, characterization and detailed studies on the coordination mode of metal(II) complexes with the (E)-N-(2-(((2-hydroxynaphthalen-1-yl)methylene)amino)phenyl)-4-methylbenzenesulphonamide ligand [H_2_L]. This molecule is a potential NNO donor ligand with weakly acidic N-H and O-H groups (Figure 1). The homoleptic complexes were prepared using an electrochemical procedure where the metal is the anode of a cell containing the ligand in solution. The presence in the electrolytic cell of the H_2_L ligand and potential co-ligands, such as 2,2′-bipyridine, 4,4′-bipyridine or 1,10-phenanthroline, allows for the synthesis of heteroleptic compounds in one step. This method has been successfully used for the synthesis of metal compounds with other ligands which have weak acid groups, such as thiol, amide or hydroxyl groups [31]. The crystal structure of six compounds with an ML or MLL′ formula synthesized in this manner has been studied by means of single crystal X-ray diffraction, and the supramolecular network was confirmed by the study of the Hirshfeld surfaces, which can be found in the Supplementary Materials together with the 2D fingerprint plots which are an expression of all contacts found in the crystal structures studied in this paper [32]. The potential biological and chemical activity of these compounds is often dependent on the nature of the coordination of the ligand. For this reason, we performed electronic structure simulations to provide detail to the binding mode of the chelating complexes.

## 2. Results

The analytical data of the products synthesized show that the electrochemical procedure described can be satisfactorily used in the synthesis of homoleptic complexes. Additionally, the presence of co-ligands also allows for the synthesis of mixed or heteroleptic complexes in a single step. This method represents an expedited alternative to other standard chemical procedures.

The complexes thus obtained are air-stable solids and do not show a tendency to decompose or oxidize. They are also quite soluble in the reaction medium, so the resulting solution was air concentrated in order to obtain the corresponding solids.

The electrochemical efficiency, defined as the number of moles of metal dissolved per Faraday of charge, for the synthesis of Ni, Co, Zn and Cd complexes was found close to 0.50 mol·F^−1^. This fact, along with the evolution of hydrogen at the cathode, is compatible with the following reaction mechanisms:

Cathode: H_2_L + 2e^−^ → H_2_ + L^2−^

Anode: M → M^2+^ + 2e^−^ where M = Co, Ni, Zn or Cd

Overall: H_2_L + M → [ML] + H_2_, where M = Ni, Zn or Cd

or H_2_L + M + n L′ → [ML(L′)_n_] + H_2(g)_

where M = Co, Ni, Zn or Cd; L is the dianion due to the dehydronation of H_2_L; n = 1 for L′ = 2,2′-bipyridine or 1,10-phenanthroline; and n = ½ for L′ = 4,4′-bipyridine.

One of the obtained compounds, the cobalt compound NEt_4_[CoL_2_], contains the metal in its trivalent state, Co(III). This could be the result of aerial oxidation in the solution of the Co(II) species initially originated in the electrochemical reaction, which is a common feature of the chemistry of Co(II). Only the incorporation of N-donor co-ligands in the heteroleptic complexes would stabilize the low oxidation state for this metal, as was observed previously [31].

A number of complexes were successfully synthesized with the protocol described above, and crystals suitable for X-ray studies were obtained for six of them: NEt_4_[CoL_2_] (**1**), [NiL(H_2_O)] (**2**), [NiL(CH_3_CN)(H_2_O)] (**3**), [Ni_2_L_2_(4,4′-bpy)] (**4**), [Zn_2_L_2_(MeOH)_2_] (**5**) and [ZnL(2,2′-bpy)]·(CH_3_CN) (**6**). They were obtained via the slow air concentration of the mother liquor or crystallization at room temperature from the initial product. All compounds were also characterized by several spectroscopic techniques in solid or solution states when applicable. The compounds for which crystal structures are available have also been fully analyzed and characterized via a series of computational techniques:

1. Density functional theory simulations allowed us to add detail to the electronic structure description of these complexes, and in all cases, a good fit between the obtained crystallographic structure and the optimized structure was obtained.
2. The Quantum Theory of Atoms in Molecules was applied to offer deeper insight into the nature of key ligand-to-metal interactions.
3. Hirshfeld surface analysis was conducted to provide a description of the supramolecular structure in the crystal lattice (due to the extension of this latter analysis, it has been consigned to the supporting information).

### 2.1. Description of the Structure of NEt_4_[CoL_2_] (***1***)

**Figure 2:**
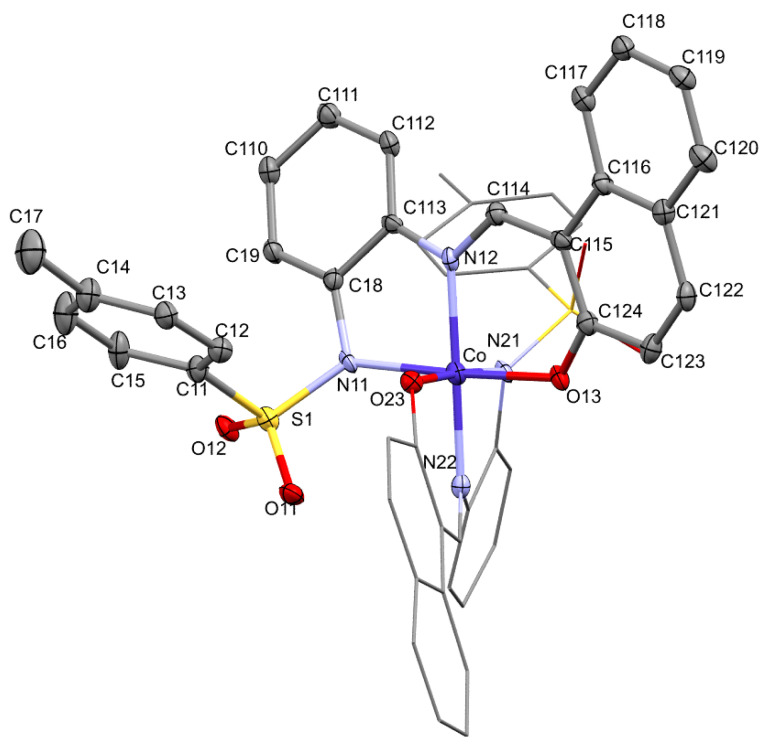
Crystal structure of anion [CoL_2_]^−^. One of ligands’ molecules drawn at 30% probability level. Tetraethylammonium cation was not drawn for clarity.

Figure 2 shows the anion of NEt_4_[CoL_2_] (**1**) together with the atom labeling scheme adopted. The structural analysis of the compound shows the presence of the anion complex [CoL_2_]^−^ balanced by a tetraethylammonium cation whose origin must be in the NEt_4_ClO_4_ added to the electrolyte solution to enhance the initial conductivity (see Section 4). In the anion, the metal atom is coordinated to two tridentate dianionic ligands that bind to the metal through amidate and imine nitrogen atoms and the oxygen atom from the naphtholate fragment. The geometry around the metal center can be described as a slightly distorted octahedral one, with each ligand adopting a meridional disposition. Thus, the trans and cis bond angles are close to the expected values except for those corresponding to five member chelate rings, whose values are slightly lower. The cobalt atom lies 0.012(4) Å and 0.011(4) Å out of the planes formed by the three donor atoms of each of the ligands, which form between them a dihedral angle of 87.9(2)°, close to the theoretical value for an octahedron.

The bond distances (See Table 1) between cobalt and the nitrogen atoms of the ligand indicate different degrees of interaction between the metal and these donor atoms, in such a way that the largest one corresponds to the deprotonated amidate group. This relative trend is also observed with all other complexes described here, except for Zn (see below). In anion [CoL_2_]^−^, the bond distances involving the metal and iminic nitrogen atoms [1.871(8) and 1.867(8) Å], sulphonamide nitrogen atoms [1.967(7) and 1.973(8) Å] and naphtholate oxygen atoms [1.875(6) and 1.876(6) Å] could be considered normal and comparable to those found in other six-coordinated Co(III) compounds containing similar ligands [33,34,35]. A detailed study of the supramolecular network, supported on the analysis of the Hirshfeld surfaces and the 2D fingerprint plots which are an expression of all contacts, can be found in the Supplementary Materials for this, and also for the other crystal structures studied in this paper.

#### DFT Studies on NEt_4_[CoL_2_] (**1**)

**Figure 3:**
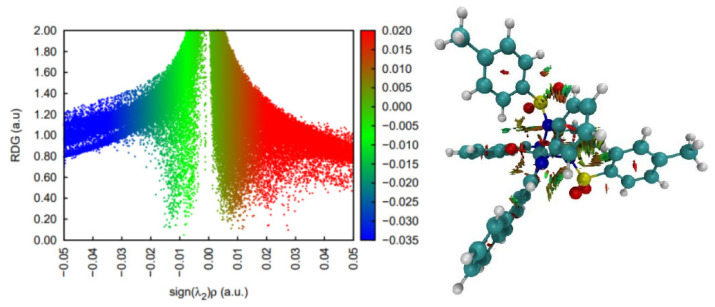
Non-covalent interaction analysis for [CoL_2_]^−^. Residual density gradient plot (**left**) and 3D plot with weak interactions (**right**).

DFT simulations and analyses of non-covalent interactions suggest that this complex is most stable as a singlet state and displays a wide range of stabilizing interactions, most of which are weak interactions (Figure 3). There are also some steric contacts and destabilizing interactions in the pose of the crystal structure. In terms of stabilizing interactions, two CH-π contacts and two hydrogen bonds are worth mentioning. The latter are, however, weaker than the usual polar hydrogen bonds, since the hydrogen involved is attached to a carbon atom. The remaining stabilizing contacts seem to be very weak and dispersion related.

### 2.2. Description of the Structure of the Nickel Compounds [NiL(H_2_O)] (***2***), [NiL(CH_3_CN)(H_2_O)]_2_ (***3***) and [Ni_2_L_2_(4,4′-bpy)] (***4***)

**Figure 4:**
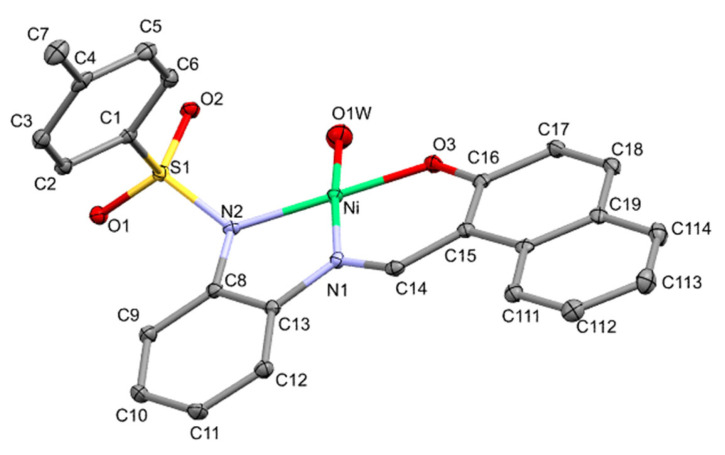
ORTEP view of [NiL(H_2_O)].

**Figure 5:**
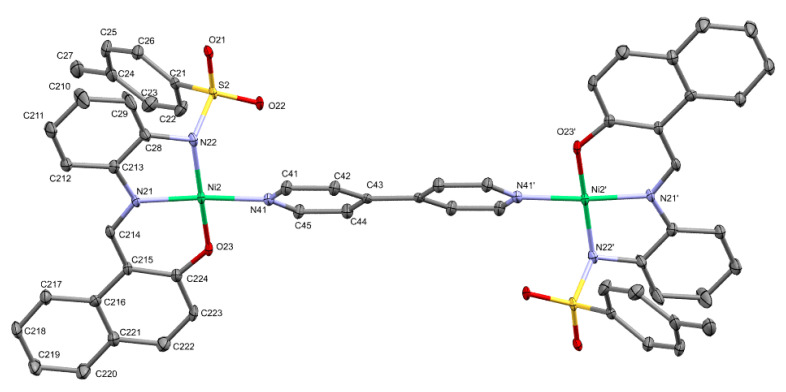
ORTEP drawn of [Ni_2_L_2_(4,4′-bpy)], [i = 1−x, 1−y, 1−z].

**Figure 6:**
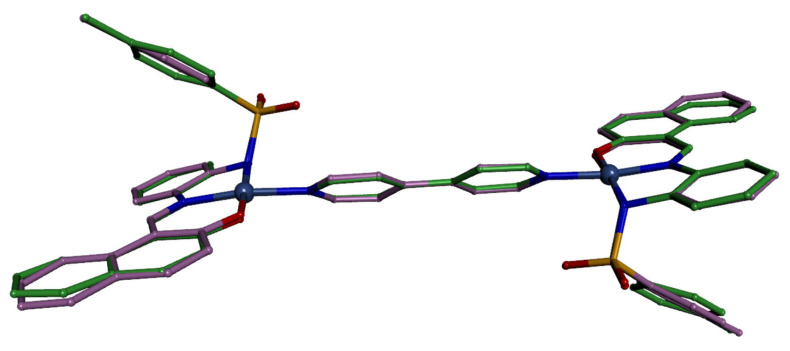
[Ni_2_L_2_(4,4′-bpy)]. Superimposition of both molecules, showing scarce difference between them. Carbon atoms in green or in dull magenta for each molecule.

Figure 4 shows the crystal structure of the first isolated nickel compound in our study together with the atom labeling scheme adopted. The recrystallization of this compound in acetonitrile yields another compound that incorporates the solvent in the coordination sphere, as is shown in Figure 5. The incorporation into the electrochemical cell of other additional ligands provides new compounds. [Ni_2_L_2_(4,4′-bpy)] was crystallized (see Figure 6) and included in the study. Table 2 provides a selection of bond lengths for the three nickel complexes here described, and is ordered to show the differences between related bonds depending on the coordination number: four for this one and also for [Ni_2_L_2_(4,4′-bpy)], and six for the dimeric [NiL(CH_3_CN)(H_2_O)]_2_.

The structural study of compound **2** via X-ray diffraction shows the presence of a mononuclear species of nickel(II), in which the metal atom is tetracoordinated via the imino and amidate nitrogen atoms and the oxygen atom of the naphtholate fragment of the tridentate Schiff base and an oxygen atom of a molecule of water. The environment around the metal is square planar [NiO_2_N_2_], with values for bond angles for those in the *trans* position near 180° [179.59(6) and 173.32(7)°] and in the range of 83.73(7)–95.96(7)°] for those in the *cis* position, and it shows little distortion with respect to the regular geometry. Surprisingly, the free (or non-chelating) coordinated water molecule is implicated in the most deviated angles even considering that the other *cis* angles are in the five or six membered metallacycles, where the chelating bite conditioned this bond angle. This deviation of the water molecule could be due to the hydrogen bond network discussed in the Supplementary Materials. The four donor atoms form a plane with a maximum deviation of 0.0547 Å. The nickel atom is located at 0.0490(9) Å above the plane originated by the four donor atoms. The square planar geometry has a slight distortion of a tetrahedral type, as inferred from the value of 6.44(11)° found for the dihedral angle between the chelate plane Ni-N-N and the Ni-O-O plane. Both metallacycles are more or less planar, with the five-member one somewhat distorted, with Cremer–Pople puckering parameters [36] Q(2) of 0.1166(15) Å, and ϕ(2) of 182.4(9)°, showing a conformation defined as an envelope on the position occupied by the Ni atom. The Ni-N bond distances between the metal atom and the amidate and imine nitrogen atoms are different; the largest one corresponds to the deprotonated amidate group, similar to other Ni and Co complexes here described, and also to Cu compounds described elsewhere [27]. The Ni-N(imino) bond distance [1.851(2) Å] is very similar to those found in other nickel square planar complexes with Schiff bases, as is the Ni-N(amidate) bond distance [1.909(2) Å] [36,37,38]. The bond length between the nickel atom and the oxygen atom from the naphtholate group [1.8277(13) Å] could also be comparable with other planar tetracoordinated compounds with this ligand moiety [39]. In contrast, the value of the bond distance between the nickel atom and the oxygen atom of the water molecule [1.930(2) Å] is quite short when compared with other nickel(II) compounds with a similar environment [40,41].

The compound [Ni_2_L_2_(4,4′-bpy)] (**4**) is also a planar tetracoordinated nickel compound, and due to the nature of the 4,4′-bpy ligand, it generates a dinuclear complex. The geometry of both metal atoms in the complex can be considered to be distorted square planar (r.m.s = 0.1230 Å), with the nickel atom 0.0267 Å out of the plane. The metal environment shows a slight distortion towards a tetrahedral geometry, this time being more pronounced than in [NiL(H_2_O)] (dihedral angle 11.1(3)°, in average). The τ_4_ value [42,43] is now 0.11 for both nickel atoms in this compound. The bond distance between the nickel atom and the nitrogen atom of the molecule of 4,4′-bipyridine, Ni-N(bpy) [1.922(4) and 1.939(4) Å] is also similar to those found in other Ni(II) complexes with N4O2 coordination including 4,4′-bipyridine in the coordination sphere [44]. It is worth noting that [Ni_2_L_2_(4,4′-bpy)] (**4**) crystallizes in the triclinic system, space group *P*-1. The asymmetric unit contains two halves of two molecules. The other two halves are symmetry-generated. In Figure 5, for clarity, just one of the molecules was drawn (two halves). Note that the geometrical parameters for both molecules are identical (within the experimental errors, Table 2). Both dinuclear units were generated by an inversion center located in the middle of the bond linking the two 4,4′-bipyridine rings; one of these units is shown in Figure 5. Figure 6 shows a superimposition of the two molecules to highlight their similarities.

**Figure 7:**
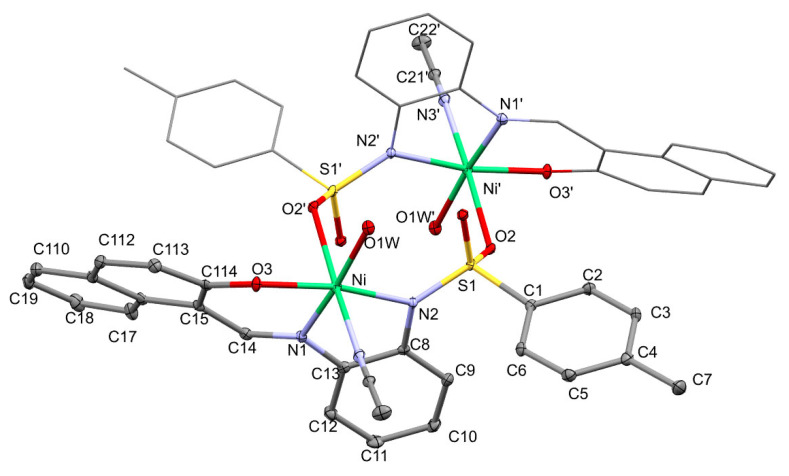
ORTEP drawn of [NiL(CH_3_CN)(H_2_O)]_2_, [i = −x, −y, −z].

On the other hand, recrystallization of (**2**) in acetonitrile gives an octahedrally coordinated compound (**3**)**,** showed in Figure 7. The asymmetric unit contains one half of the molecule. The presence of one of the oxygen atoms from the sulphonic groups as a donor is not strange; it was found several times in our previous research [45] and, in the case of Ni(II) ions, this behavior was also reported for a nickel complex with (μ_2_-4-amino-N-(pyrimidin-2-yl)benzenesulphonamide) as a ligand [46], which is, additionally, a clear example of a chalcogen bond (ChB) [47]. It should be noted that the distance between the nickel atom and the sulphonyl oxygen atom is the longest in the octahedron, 2.2069(15) Å, about 0.2 Å longer than the other distances. The nitrogen atom N(4) from the acetonitrile molecule occupies the trans position to this atom in one of the 4-fold axes. The three donor atoms of the Schiff base ligand and the oxygen atom of the water molecule are in the basal plane. The situation of these four donor atoms and the nickel atom is not exactly coplanar (r.m.s = 0.0775 Å). Atoms N(1) and O(1W), in trans positions, are both below the plane (−0.085(1) and −0.0693(8) Å), while the atoms N(2) and O(3) are above the plane (0.0756(9) and 0.079(1) Å). Meanwhile, the nickel atom is out of the plane (0.024(1) Å), and as a consequence, the bond angles between O(3)-Ni(1)-N(2) 169.45(7)° and N(1)-N(1)-O(1W) 174.15(8)° differ significantly from the expected 180°. One of the major causes of distortion with respect to a regular geometry is the small bite of the five-membered chelate ring N(1)-Ni(1)-N(2), 81.05(8)°, a similar value to that found for the other complexes in this paper. As in the previous complexes, the five-membered ring is somewhat puckered (with the envelope conformation with the Ni atom out of the plane; Q(2), 0.1975(19) Å; ϕ(2), 177.4(7)°), but this time the six-membered ring is also slightly puckered; Q, 0.4015(17) Å, θ, 63.7(3)°; ϕ, 4.2(4)°. Again, the bond distances between the nickel and imine and amidate nitrogen atoms are different, with the imine corresponding to the shorter one Ni-N(1) 1.9898(19) Å. As a consequence of the different coordination numbers, these distances are now longer than those found in [NiL(H_2_O)] **2**, although they can still be considered normal when compared with hexacoordinate complexes with similar ligands [48,49,50]. The values of the bonds Ni-O_naphtholate_ [2.0009(16) Å], Ni-O_water_ [2.0527(17) Å] and Ni-N_acetonitrile_ [2.099(2) Å] are also similar to other hexacoordinated nickel(II) complexes with these coordinated fragments [51,52].

#### DFT Studies on [NiL(H_2_O)] (**2**), [NiL(CH_3_CN)(H_2_O)]_2_ (**3**) and [Ni_2_L_2_(4,4′-bpy)] (**4**)

**Figure 8:**
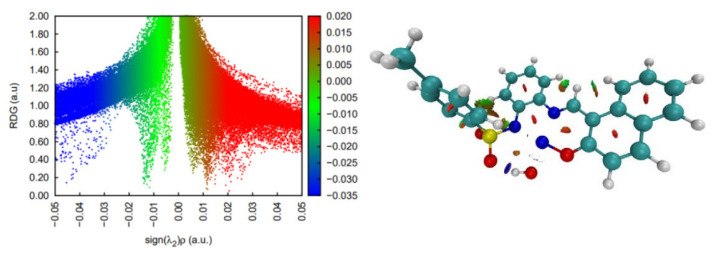
Non-covalent interaction analysis for [NiL(H_2_O)]. Residual density gradient plot (**left**) and 3D plot with weak interactions (**right**).

DFT simulations suggest that this complex is stable in its triplet state, although a low-lying singlet configuration is only 3 kcal/mol higher in energy, which may make both configurations easily available at ambient temperature. Its non-covalent interactions analysis clearly reveals an intramolecular hydrogen bond that pulls the water ligand towards the sulphonamide oxygen atom and a handful of weaker dispersion interactions (Figure 8).

**Figure 9:**
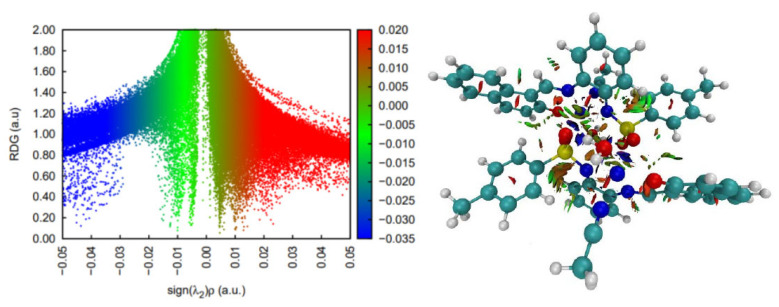
Non-covalent interaction analysis for [NiL(CH_3_CN)(H_2_O)]_2_ (**3**). Residual density gradient plot (**left**) and 3D plot with weak interactions (**right**).

DFT calculations on the spin multiplicities for complex **3** strongly suggest that the Ni center is most stable as a quintet; hence, it features a high spin configuration. This state seems to lay about 10 kcal/mol below the singlet state configuration and more than 14 kcal/mol below the triplet spin state. In terms of non-covalent interactions within the complex, strongly stabilizing contacts are found (in blue in Figure 9) mainly between water units and the S=O bonds of the sulphamide groups. In terms of the supramolecular structure, the two units of the dimeric structure seem to be organized around interactions of the Ni atom of one unit with the sulphonamide oxygen atoms of the second unit.

**Figure 10:**
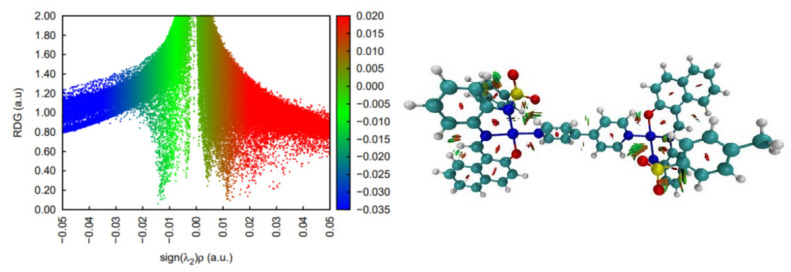
Non-covalent interaction analysis for [Ni_2_L_2_(4,4′-bpy)]. Residual density gradient plot (**left**) and 3D plot with weak interactions (**right**).

We also performed DFT simulations on the structure of [Ni_2_L_2_(4,4′-bpy)] to account for spin multiplicity. Geometry optimizations on the dinuclear complex led to structures in which a quintet and a triplet state were low-lying states, about ~2 kcal/mol below the singlet state. Geometry superposition of the optimized triplet and quintet states, however, displayed important deviations with respect to the crystal structure. The singlet state, however, provided a very good fit with the crystallographic data. With this evidence in hand, we concluded that this dinuclear complex in the solution may actually present a high spin configuration, although its crystalline packing forces a conformation where the low spin state is more stable. We obtained spin densities for both the triplet and quintet states and observed that they are concentrated around one of the Ni centers, with negligible spin polarization of the ligands in both cases. In terms of non-covalent interactions, this complex features stabilizing O···H and N···H interactions between the ortho hydrogen atoms of the bipyridine moiety and oxygen and nitrogen atoms from the tridentate ligand. The latter also displays weak O···H intraligand interactions. Additionally, dispersion-type interactions are found between the pyridine N atom and an oxygen atom from the SO_2_ group (See Figure 10).

### 2.3. Description of the Structure of [Zn_2_L_2_(MeOH)_2_] (***5***) and [ZnL(2,2′-bpy)]·CH_3_CN (***6***)

**Figure 11:**
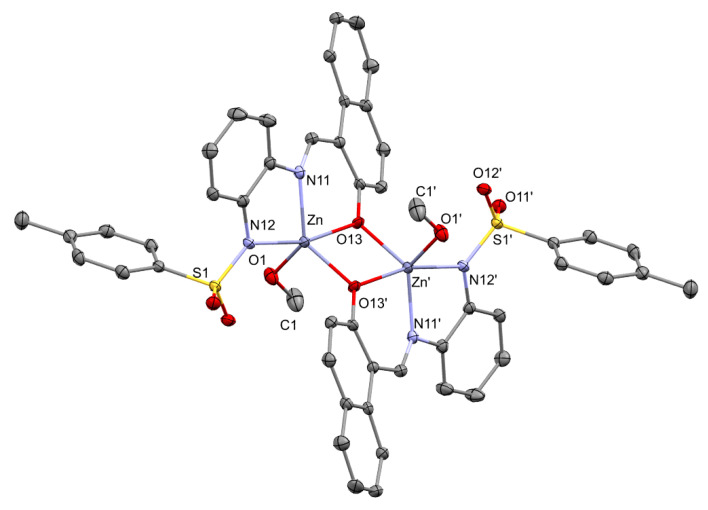
Crystal structure of [ZnL(MeOH)]_2_.

Two zinc complexes were synthesized and fully characterized in this work, one with two units of methanol as a co-ligand and another including a 2,2′-bipyridine ligand. Interestingly, these two complexes are remarkably different in a number of aspects, stemming from the fact that one is a dinuclear complex (Figure 11), whereas the second is mononuclear (see below). Some geometrical features, however, maintain similarity. For this reason, and for the sake of comparison, a selection of related bond lengths and bond angles is provided in Table 3.

The structure of [ZnL(MeOH)]_2_ consists of a dimeric adduct, including two units of [ZnL(MeOH)] in which the zinc atoms are linked by two bridging naphtholate oxygen atoms. The molecule has an inversion center at the middle of the 4-membered ring Zn(1)O(1)Zn(1^i^)O(1^i^), symmetrically flat although slightly asymmetrical in their distances Zn(1)-O(1^i^) of 1.9951(17) Å and Zn(1)-O(1) of 2.0666(17) Å. The presence of the phenolic oxygen as a bridge is not infrequent. A. W. Kleij and coworkers, for example, have described a number of compounds where the oxygen atom of a 2-oxynaphthyl (or related group) acts as a bridge, even when two bulky t-butyl substituents are present in the vicinity of such a bridging atom [53]. The distance between the two zinc atoms, 3.1572(13) Å, similar to that found in other dinuclear complexes of zinc(II) with phenoxo bridges, is high enough to suggest the absence of an orbital interaction between the two metal atoms [54,55].

In each symmetry-related unit, the zinc atom is pentacoordinated by the imine and amide nitrogen atoms, the oxygen atom of the naphtholate the ligand and another oxygen atom belonging to the naphtholate fragment of the other monomer. The coordination around the metal is completed with the oxygen atom of one molecule of methanol, a solvent used in the crystallization of the compound. Thus, the metal is in a pentacoordinated [ZnO_3_N_2_] environment.

The geometry around each of the zinc atoms can be described as highly distorted; a value between a trigonal bipyramid and a square pyramid is obtained for the well-known τ_5_ parameter, 0.61 [56]. Assuming a much-distorted trigonal pyramidal geometry, each tridentate ligand occupies the two axial positions and one equatorial site. The remaining coordination positions in the equatorial plane are occupied by a bridging naphtholate oxygen atom and by the oxygen atom of the methanol ligand. However, due to the distorted shape, the axis is bent to an angle of 165.57(7)° and the other values differ largely from the theoretical 90 or 120 degrees.

The bond lengths between the zinc atom and the naphtholate oxygen atoms, although significantly different between them ([O(11)-Zn(1^i^) 1.9951(17) Å and O(11)-Zn(1) 2.0666(17) Å]), are within the range of mean values observed in other Zn(II) pentacoordinated complexes with Zn-O(naphtholate) bridges mentioned above [57]. It is worth noting that the Zn-N(amide) bond length is slightly shorter than the Zn-N(imine) one. This situation is different from what we found in the transition metal complexes described earlier in this paper and marks the difference between transition metals and group 12 metals. The value of the distance of Zn-O(methanol) [2.069(2) Å] is in the normal range for a neutral MeOH ligand coordinated to a pentaccordinated Zn(II) atom [58,59] and is expectedly longer than what is observed for the anionic form of the methanide ligand [60]. The two metallacycles are, again, puckered, with both showing an envelope conformation [five-member ring, Q(2), 0.186(2) Å; ϕ(2), 180.8(8)°; six-member ring, Q, 0.3914(16) Å; θ, 54.8(3)°; ϕ, 17.5(4)°].

**Figure 12:**
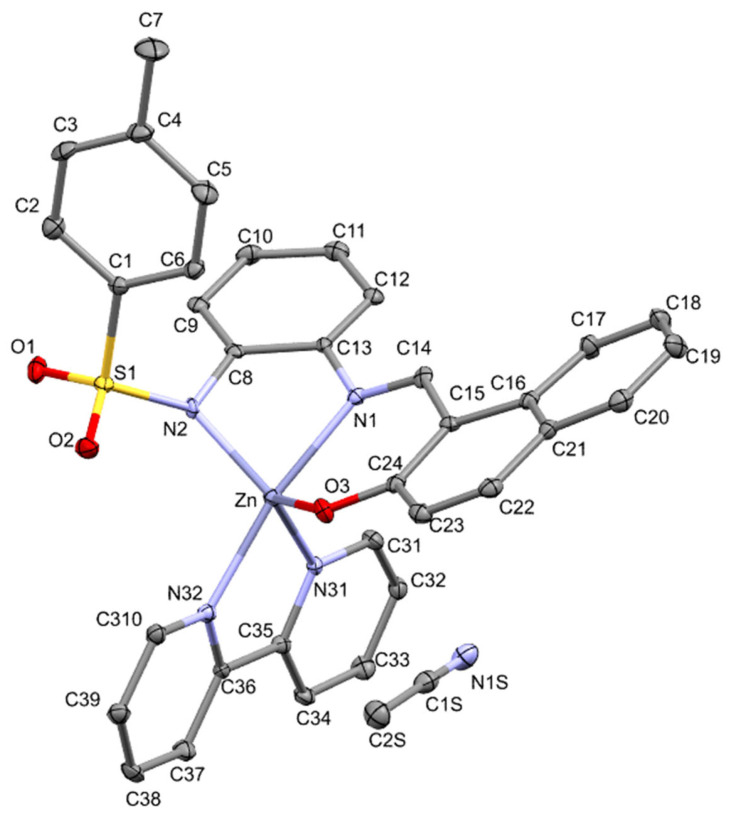
Crystal structure of [ZnL(2,2′-bpy)]·CH_3_CN (**6**).

On the other hand, the structure of [ZnL(2,2′-bpy)]·CH_3_CN contains the tridentate dianionic Schiff base coordinating to the metal by using both the amide and imine nitrogen atoms and also the naphtholate oxygen atom, as in the other complexes in this paper (see Figure 12). Two nitrogen atoms of a bipyridine ligand complete the coordination sphere up to a pentacoordinated Zn atom in an overall coordinative environment that somewhat resembles complex **5**. The geometrical parameter τ_5_, which gives information about the geometry, also has a similar value: 0.65 (vs. 0.61 for [ZnL(MeOH)]_2_). If we choose a trigonal bipyramid as a descriptor, the basal plane of the bipyramid would comprise the amide group nitrogen, the oxygen of the naphtholate and one of the nitrogen atoms of the 2,2′-bipyridine ligand. The axial positions would be occupied by the other nitrogen of this co-ligand and the imine nitrogen atom, forming an angle of 169.6(1)° (compared with the regular 180°). Basal plane bond angles range from 105.6(1) to 130.5(1)° and those angles with expected theoretical values of 90° go from 77.8(1) to 108.5(1)°. As it is in the other complexes described in this paper, the small bite of the five-membered chelate ring is an important source of distortion, and this complex contains an additional five-membered ring due to the bipyridine. These N-Zn-N bond angles are 79.4(1) and 77.8(1)°. The Zn-O(naphtholate) bond distance [1.953(3) Å] is shorter than those found in the previously described [ZnL(MeOH)]_2_ [1.995(2) and 2.069(2) Å], but it should be remembered that in the latter, those atoms act as bridges between two zinc centers. The Zn-N(amide) bond length, 2.040(4) Å, is shorter than the Zn-N(imine) bond length, 2.094(3) Å, as it is in [ZnL(MeOH)]_2_ (**5**). Finally, the two Zn-N(bpy) bond distances [2.117(3) and 2.119(3) Å] show a symmetric coordination mode for this ligand [57].

#### DFT Studies on [Zn_2_L_2_(MeOH)_2_] (**5**) and [ZnL(2,2′-bpy)]·CH_3_CN (**6**)

**Figure 13:**
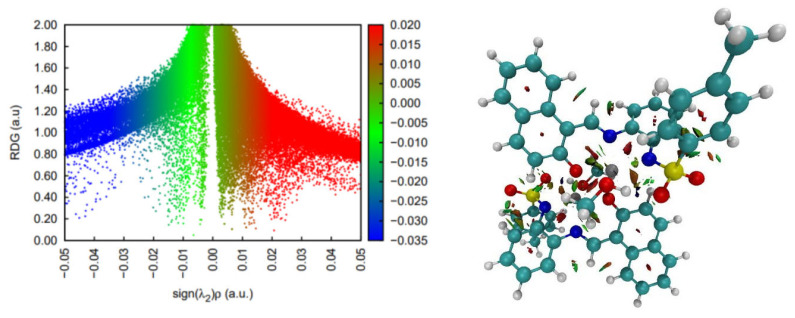
Non-covalent interaction analysis for [Zn_2_L_2_(MeOH)_2_]. Residual density gradient plot (**left**) and 3D plot with weak interactions (**right**).

The structure of [Zn_2_L_2_(MeOH)_2_] (**5**) presents a high degree of non-covalent O···H and N···H interactions, which are probably responsible for the formation of the dimer. Our simulations from the perspective of non-covalent interactions and the quantum theory of atoms in molecules concur with the starting hypothesis that there is no bonding interaction between the Zn centers (Figure 13).

**Figure 14:**
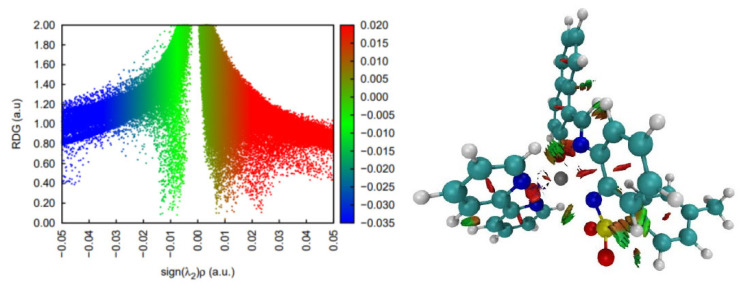
Non-covalent interaction analysis for [ZnL(2,2′-bpy)]. Residual density gradient plot (**left**) and 3D plot with weak interactions (**right**).

For the mononuclear [ZnL(2,2′-bpy)], in terms of non-covalent interactions (Figure 14), it is interesting to note that the tridentate ligand and the bipyridine unit clearly establish two stabilizing interactions involving heteroatoms on the L side and both ortho hydrogen atoms from the bidentate ligand. The resulting O···H and N···H contacts could be partly responsible for the distorted geometry around the metal center.

### 2.4. Metal-N Bond Length Conclusions

As indicated in the discussion above, one interesting observation in the course of this investigation is the fact that the Schiff base type ligand shows a metal–nitrogen bond length which is systematically shorter than the equivalent bond of the sulphonamide fragment when the metal is Co and Ni; however, the opposite relationship is observed for the Zn complexes. In an attempt to bring light onto this observation, we decided to resort to the Quantum Theory of Atoms in Molecules [61] and to analyze the properties of the electron density at the bond critical point (the point along a bond where the density is minimal in the direction of the bond). The laplacian of the electron density at such a point yields mathematical information on whether a local accumulation or depletion of electrons occurs in the vicinity of such a point, which provides a good measure for the covalent or ionic character of the bond itself. When the laplacian of the electron density is positive, it means that the electron density is locally depleted in the vicinity, and this situation usually signals a bond with an ionic character. A local charge concentration at the bond critical point, on the contrary, is normally a signature for a bond with strong covalent character. Inspection of the laplacian at the M···N bond critical points in these complexes reveals larger and positive values when M is Co and Ni; hence, these bonds are driven mostly by ionic interactions, and the Schiff base nitrogen atom seems to establish these interactions in a more efficient manner than the deprotonated sulphonamide. When the metal center is Zn, however, the laplacian shows much lower values for [Zn_2_L_2_(MeOH)_2_] (**6**) (see Supp. Information), which indicates that this metal center requires interactions to be much more covalent in nature. Under this paradigm, it seems that the sulphonamide nitrogen atom is capable of a more efficient covalent interaction. We assume that the probable sp^3^ nature of the lone pair in sulphonamide vs. the sp^2^ of the lone pair in the Schiff base could be partially responsible for this enhanced covalent interaction, since the lower the s character in the interacting hybrid, the more directional this hybrid is, hence allowing a greater overlap with the available orbitals at the metal center.

## 3. Spectroscopic Studies

### 3.1. IR Spectrocopy

The IR spectra of the metallic complexes (see Section 4 and also the Supplementary Materials) do not show the bands attributable to the stretching vibration ν(O-H) and ν(N-H), which in the free ligand appear at 3450 and 3264 cm^−1^, respectively. This is an indication of the deprotonation of the ligand during the electrochemical procedure and that the ligand is in a dianionic form coordinating to the metal through the amide nitrogen atom and the naphtholato oxygen atom. In addition, the spectra of all compounds show a strong band at 1600–1615 cm^−1^, shifted ca 20 cm^−1^ to a lower frequency with respect to the free ligand as a consequence of the coordination to the metal with the imine nitrogen atom. Two strong bands in the spectra of the complexes, in the ranges of 1330–1344 cm^−1^ and 1120–1144 cm^−1^, slightly shifted with respect to the free ligand, are assigned to the ν_as_(S=O) and ν_s_(S=O) vibration group. The spectra of the complexes also show additional medium–low-intensity bands in the range 3070–2874 cm^−1^, which correspond to the ν(CH_3_). In the complexes containing water, a broad band at about 3420–3500 cm^−1^ is also observed, and those containing acetonitrile are characterized by a medium intensity band around 2185–2195 cm^−1^, attributable to the ν(C=N) fragment. In the heteroleptic complexes, additional bands at 770 and 730 cm^−1^; 1600, 1530 and 1000 cm^−1^, and 1510, 850 and 730 cm^−1^ are also observed, which are typical of coordinated 2,2′-bipyridine, 4,4′-bipyridine and 1,10-phenanthroline, respectively [26,62].

### 3.2. Mass Spectra

The FAB mass spectra (see Section 4) show, in practically all cases, the peak associate to the molecular ion and also peaks corresponding to fragments from the molecular species. Noteworthy are the FAB mass spectrum of NEt_4_[CoL_2_], because in addition to the peak at *m*/*z* 1018 assignable to the molecular ion, a peak at *m*/*z* 473 is assignable to the fragment [CoL] formed by the loss of the tetraethylammonium ion and one of the Schiff base ligands. In the same way, the FAB mass spectrum of the compound [NiL(CH_3_CN)(H_2_O)]_2_, the peaks of the dimeric [Ni_2_L_2_] and monomeric [NiL] species are also observed at *m*/*z* 945 and 472, respectively, and also the fragment [NiL-Ts] at *m*/*z* 317 resulting from the loss of the tosyl group from the monomeric species, thus confirming the structures of the compounds described earlier. The FAB spectra for the heteroleptic compounds (containing 2,2′-bipyridine or 1,10′-phenanthroline as a co-ligand) are also remarkable because they show the peaks corresponding to the molecular monomeric species [ML(bpy)]^+^ or [ML(phen)]^+^ and also the peak corresponding to [ML] resulting from the loss of the co-ligand. For species [M_2_L_2_(4,4′-bpy)], although the peak of the dimeric species is not observed, peaks attributable to [M_2_L_2_(4,4′-bpy)] -Ts and the monomeric [ML(4,4′-bpy)] are observed.

### 3.3. Electronic Spectroscopy

The diffuse reflectance spectrum of NEt_4_[CoL_2_] is characteristic of six-coordinated low spin Co(III) complexes with an octahedral stereochemistry. The compound shows in the UV–visible region two allowed spin transitions at 15,670 cm^−1^ and 21,180 cm^−1^ assignable to ^1^A_1g_ → ^1^T_1g_ and ^1^A_1g_ → ^1^T_2g_, transitions in an octahedral environment around the metal, as confirmed by X-ray diffraction (vide supra) [63,64].

The diffuse reflectance spectra of the two heteroleptic Co(II) complexes, [CoL(2,2′-bpy)] and [CoL(phen)] (see Section 4), are similar and characteristic of five-coordinate complexes with a distorted trigonal bipyramidal geometry. Although it is difficult to unequivocally assign the bands observed to specific electronic transitions, according to the level energy diagram of low-spin Co(II) in a trigonal bipyramidal field, the bands in the visible region at 8750–9000, 15,240–15,440 and 18,750–20,800 cm^−1^ are assigned ^4^A_2_′(F) → ^4^E″(F)″, ^4^A_2_′(F) → ^4^A_2_′(P) and ^4^A′(F) → ^4^E″(P) transitions, respectively.

The diffuse reflectance spectrum of heteroleptic [Co_2_L_2_(4,4′-bpy)] shows the characteristics of species with a tetrahedral environment around the cobalt metal. The spectrum shows a band at 15.770 cm^−1^ and other broad bands around 7.650–8800 cm^−1^. The band in the visible region is assignable to a ^4^A_2_ → ^4^T_1_(P) (ν_3_) transition, and the multicomponent band located in the near-infrared region to ^4^A_2_ → ^4^T_1_(F) (ν_2_). The transition ^4^A_2_ → ^4^T_2_ (ν_1_) was not observed, but its position could be calculated (4872 cm^−1^) from the position of the band assigned to ν_3_, taking ν_2_ as the center of the multiple band according to the procedure followed by Lever [63] (ca. de 8.225 cm^−1^). The splitting observed for ν_2_ in the spectrum of [Co_2_L_2_(4,4′-bpy)] can be attributed to the distortion of the tetrahedral symmetry, a regular situation that arises from the non-equivalence of the donor atoms and small values of the bite angles of the chelate ligands.

The electronic spectrum of the complex of [NiL(CH_3_CN)(H_2_O)]_2_ is consistent with an octahedral geometry around the metal as confirmed by X-ray diffraction data. Thus, the electronic spectrum of the complex shows bands at 8750, 15,770 and 20,570 cm^−1^, which are within the expected range for octahedral complexes of Ni(II), and they can be assigned to transitions ^3^A_2g_ → ^3^T_2g_ (ν_1_), ^3^A_2g_ → ^3^T_1g_(F) (ν_2_) and ^3^A_2g_ → ^3^T_1g_ (ν_3_), respectively. In the diffuse reflectance spectra of heteroleptic compounds of nickel (II) with 2,2′-bipyridine and 1,10-phenanthroline as co-ligands (4000 to 30,000 cm^−1^), several bands are observed in the UV-vis which may be assigned to d-d bands, charge transfer and/or transitions from the ligand itself. In the complexes, bands are observed in the environment to 7750, 11,200, 15,600 and 21,000 cm^−1^. These bands are located within the range of accepted values for pentacoordinated high-spin complexes of nickel (II) in square pyramid environments, and can be assigned to the ^3^B_1_ → ^3^E, ^3^B_1_ → ^3^B_2_, ^3^B_1_ → ^3^E and ^3^B_1_ → ^3^A_2_,^3^E (P) transitions, respectively.

### 3.4. NMR Spectra

The ligand and the diamagnetic complexes were studied in solution using ^1^H NMR spectroscopy. The spectra were in all cases recorded in DMSO-d6 as deuterated solvents due to the low solubility of complexes in other least coordinative solvents (see the Supplementary Materials).

The ^1^H NMR of the complexes show, in all cases, the disappearance of the signals corresponding to the hydrogens of the -NH (δ = 14.40 ppm) and –OH groups (δ = 9.88 ppm), thus confirming their deprotonation in the electrochemical process and consequently their coordination to the metal in dianionic form. Moreover, the imino group signal undergoes extensive downfield shift in the complex, about 0.4 to 0.7 ppm, when compared with the same signal in the free ligand (δ= 9.08 ppm). This is interpreted to be because of the coordination of the Schiff base to the metal through the imino nitrogen, which causes a change in the electron density of the C = N, which leads to an imine proton deshielding. Furthermore, in the spectra of the cadmium complexes, two low-intensity peaks around this signal are observed, and these correspond to the coupling of the imine hydrogen with the ^113^Cd isotope, with values of ^3^*J*(^1^H-^113^Cd)] in the range of 26–27 Hz. This coupling provides evidence for coordination of to the metal through the imino nitrogen in solution. A higher field in the range of 7.94–6.20 ppm can be seen in a group of signals corresponding to aromatic hydrogens, and singlet signals corresponding to the methyl groups are in the intervals δ 2.14 to 2.30 ppm. In the ^1^H NMR spectra of heteroleptic complexes of zinc and cadmium, in addition to the signals corresponding to the Schiff base ligand, signals attributable to co-ligand protons of 2,2′-bipyridine, 4,4′-bipyridine or 1,10-phenanthroline could be observed (see Section 4), although in some cases, these signals are partially overlapped with those of the hydrogen atoms of the Schiff base.

## 4. Experimental Section

### 4.1. Materials and Methods

All solvents, 4-toluenesulphonyl chloride, 1,2-diamino-benzene, 2,2′-bipyridine, 4,4′-bipyridine, 1,10-phenanthroline monohydrate and 2-hydroxy-1-naphthaldehyde, and all other reagents were commercial products and were used as supplied. Cobalt, nickel, zinc and cadmium plates were purchased from Aldrich and used as 2 × 2 cm plates. The precursor N-tosyl-1, 2-diaminobenzene was prepared via the reaction of 4-toluenesulphonyl chloride and 1,2-diamino-benzene in pyridine, as described in the literature [65].

### 4.2. Synthesis of the Ligand and the Metal Complexes

#### 4.2.1. Synthesis of the Ligand

The ligand H_2_L was prepared following the procedure described previously [27] by condensing equimolar amounts of 2-hydroxy-1-naphthaldehyde (0.60 g, 3.84 mmol) and N-tosyl-1,2-diaminobenzene (1.0 g, 3.82 mmol) in ethanol (60 mL), with p-toluensulphonic acid as a catalyst, using a Dean–Stark trap. After removal of the water produced in the reaction, the solution was concentrated and the isolated solid was recrystallized from ethanol. Its purity was confirmed by the following data. Elemental analysis calc. (%) for C_24_H_20_N_2_O_3_S (416.49): C, 69.21; H, 4.84; N, 6.73; S, 7.70. Found: 68.91; H, 4.97; N, 6.61; S, 7.51. IR (KBr, cm^−1^): 3450 (m), 3264 (m), 3069–3030 (w), 1625 (s), 1548 (m), 1331 (s), 1290 (s), 1162 (s), 760 (m), 750 (m). ^1^H NMR (DMSO-d6, ppm) 14.40 (s, H, NH), 9.88 (s, H, OH), 9.08 (s, H, HC=N), 8.30–7.00 (m, 10H, phenyl), 1.82 (s, 3H, CH_3_). MS (FAB): *m*/*z*: 417 [H_2_L^2^], 262 [H_2_L^2^]-Ts. Its IR and ^1^H NMR spectra can be found in the Supplementary Materials.

#### 4.2.2. Electrochemical Synthesis of the Complexes

The electrochemical method used in the synthesis of the metal complexes was similar to that described previously [28]. The cell consisted of a tall form beaker (100 mL) fitted with a rubber bung through which the electrical leads passed. An acetonitrile (50 mL) solution of the corresponding Schiff base and a small amount of tetraethylammonium perchlorate (ca. 20 mg) as the supporting electrolyte was electrolyzed using a metal sacrificial anode and a platinum cathode. For the synthesis of heteroleptic complexes, the corresponding co-ligand, 1,10-phenanthroline, 4,4′-bipyridine or 2,2′-bipyridine, was also added to the solution. (Caution: Although no problems were found in this work, all perchlorate compounds are potentially explosive and should be handled in small quantities and with great care!) Direct current was obtained from a purpose-built d.c. power supply. Applied voltages of 10–16 V allowed sufficient current flow for smooth dissolution of the metal. The cells can be summarized as follows: Pt(−)/H_2_L + CH_3_CN/M(+) or Pt(−)/H_2_L + L′ + CH_3_CN/M(+), where H_2_L stands for the Schiff bases, L′ is the co-ligand and M represents the metal. At the end of the electrochemical reactions, the solution obtained was filtered to remove any impurities, and the filtrate was allowed to stand in air at room temperature until it produced a solid, which was collected, washed with acetonitrile and diethyl ether and dried in vacuo.

[CoL]. Electrolysis of 0.10 g (0.231 mmol) of ligand in acetonitrile for 1.3 h at 13 V and 10 mA resulted in the dissolution of 14.9 mg of cobalt; Ef = 0.52 mol F^−1^. At the end of the experiment, small quantities of green crystals of NEt_4_[CoL_2_] suitable for X-ray studies were isolated and the brown filtrate was slowly concentrated at room temperature to produce a dark brown powder characterized as [CoL]·2H_2_O). Anal. Calc. for C_24_H_20_N_2_O_4_SCo: (509.44) C, 56.58; H, 4.35; N, 5.49; S, 6.29. Found: C, 56.31; H, 4.58; N, 5.23; S, 6.10%. IR (KBr, cm^−1^): 3432 (m, a), 3060 (w), 2980 (w), 2924 (w), 1615 (s), 1536 (m), 1506 (s), 1395 (m), 1364 (m), 1342 (m), 1302 (m), 1277 (m), 1249 (m), 1142 (s), 1123 (m), 1088 (s), 950 (m). MS (FAB) *m*/*z*: 473 [CoL], 417 L. UV (cm^−1^): 7200–8700, 16,600, 22,040.

This experiment was repeated and more of the NEt_4_[CoL_2_] product was isolated. MS (FAB) *m*/*z*: 1018 NEt_4_[CoL_2_], 473 [CoL], 417 L. UV-vis (cm^−1^): 15,670, 21,180, 22,200, 28,900. ^1^H NMR (DMSO-d6, ppm): 9.20 (s, 1H, CH=N), 8.44–6.53 (m, 10H, phenyl), 2.14 (s, 3H, CH3), 1.12 (t, 3H, CH3), 3.17 (c, 2H, CH2).

[CoL(2,2′-bpy)]. A similar experiment (10 mA, 10V, 1.3 h) was conducted using 0.10 g (0.231 mmol) of H_2_L and 0.037 g (0.237 mmol) of 2,2′-bipyridine dissolved 14.9 mg of cobalt from the anode; Ef = 0.53 mol F^−1^. The brown solid compound obtained via slow evaporation of the resulting solution at room temperature was characterized as [CoL(2,2′-bpy)]. H_2_O. Yield: 0.0971 g (0.150 mmol, 65%). Anal. Calc. for C_34_H_28_N_4_O_4_SCo: (647.61) C, 63.05; H, 4.36; N, 8.65; S, 4.95. Found: C, 62.73; H, 4.15; N, 8.94; S, 4.73%. IR (KBr, cm^−1^): 3435 (m), 1612 (s), 1534 (m), 1505 (m), 1480 (m), 1449 (m), 1394 (m), 1363 (m), 1340 (m), 1313 (m), 1143 (m), 1121 (m), 1088 (m), 985 (s), 824 (m), 767 (m),749 (m), 730 (m). MS (FAB) *m*/*z*: 629 [CoL(2,2′-bpy)], 473 [CoL], 318 [CoL-Ts]. UV-vis (cm^−1^): 8980, 15,240, 18,750, 22,200, 29,400.

[CoL(4,4′-bpy)]. Electrolysis of H_2_L (0.10 g, 0.231 mmol) and 2,2′-bipyridine (0.037 g, 0.237 mmol) in acetonitrile for 1.3 h at 15 V and 10 mA dissolved 13.8 mg of cobalt (Ef = 0.49 mol F^−1^). Slow evaporation of the brown solution at room temperature produced a brown powder of [Co_2_L_2_(4,4′-bpy)]. 2CH_3_CN. Yield: 0.0986 g (0.083 mmol, 72%). Anal. Calc. for C_31_H_25_N_4_O_3_SCo: (592.55) C, 62.63; H, 4.25; N, 9.45; S, 5.41. Found: C, 62.49; H, 4.01; N, 9.62; S, 5.03%. IR (KBr, cm^−1^): 2174 (m), 1612 (s), 1603 (m), 1534 (m), 1480 (m), 1453 (m), 1341 (m), 1308 (m), 1139 (m), 1120 (m), 1087 (m), 988 (s), 952 (m), 811 (m), 748 (m). MS (FAB) *m*/*z*: 947 [Co_2_L_2_], 629 [CoL(4,4′-bpy)], 473 [CoL], 417 L. UV-vis (cm^−1^): 7650–8800, 15,770, 20,580, 22,100, 29,240.

[CoLphen]. A similar experiment (10 mA, 10 V, 1.3 h) using 0.10 g (0.231 mmol) of H_2_L and 0.047 g (0.240 mmol) of 1,10-phenanthroline dissolved 14.8 mg of cobalt from the anode; Ef = 0.52 mol F^−1^. Air concentration of the resulting solution yielded a brown solid identified as [CoLphen]. H_2_O CH_3_CN. Yield: 0.127 g (0.178 mmol, 77%). Anal. Calc. for C_38_H_31_N_5_O_4_SCo: (712.68) C, 64.04; H, 4.38; N, 9.82; S, 4.50. Found: C, 64.17; H, 4.39; N, 9.66; S, 4.23%. IR (KBr, cm^−1^): 1613 (s), 1598 (m), 1533 (m), 1508 (m), 1480 (m), 1428 (m), 1342 (m), 1302 (m), 1280 (m), 1247 (m), 1212 (m), 1144 (s), 1120 (m), 1088 (s), 984 (m), 844 (m), 748 (m), 724 (m). MS (FAB) *m*/*z*: 653 [CoL(phen)], 498 [CoL(phen)-Ts], 472 [CoL]. UV-vis (cm^−1^): 8750, 15,440, 20,830, 22,420, 29,760.

[NiL]. When a nickel anode was oxidized in a solution of H_2_L (0.10 g, 0.231 mmol) in acetonitrile, an applied voltage of 12 V produced a current of 10 mA, and after 1.3 h, 13.6 mg of nickel had been dissolved (Ef = 0.50 mol F^−1^). At the end of the electrolysis, a few deep green crystals of [NiL(H_2_O)] suitable for X-ray studies appeared at the bottom of the vessel. The crystals were isolated via filtration, and the filtrate was left to stand at room temperature for slow evaporation. A powder solid was collected after several days. Recrystallization from a water/acetonitrile 1:1 mixture of the solid compound produced crystals of [NiL]·H_2_O·CH_3_CN suitable for X-ray studies. Yield: 0.0762 g (0.143 mmol, 62%). Anal. Calc. for C_26_H_23_N_3_O_4_SNi: (532.24) C, 58.67; H, 4.36; N, 7.90; S, 6.02. Found: C, 59.04; H, 4.60; N, 7.83 S, 6.03%. IR (KBr, cm^−1^): 3330 (m), 2195 (m), 1616 (s), 1535 (m), 1482 (m), 1452 (m), 1390 (m), 1384 (m), 1341 (m), 1307 (m), 1276 (m), 1250 (m), 1164 (m), 1142 (m), 1122 (m), 1088 (m), 949 (m), 825 (m), 814 (m),748 (m). MS (FAB) *m*/*z*: 945 [Ni2L2], 472 [NiL], 414 L. UV-vis (cm^−1^): 8750, 15,770, 20,570, 22,200, 28,900.

[NiL(2,2′-bpy)]. A similar experiment (10 mA, 10 V, 1.3 h) containing 0.10 g (0.231 mmol) of H_2_L and 0.037 g (0.237 mmol) of 2,2′-bipyridine dissolved 13.8 mg of nickel from the anode; Ef = 0.51 mol F^−1^. Slow evaporation of the red solution at room temperature produced a red powder of [NiL(2,2′-bpy)]. Yield: 0.1162 g (0.185 mmol, 80%). Anal. Calc. for C_34_H_26_N_4_O_3_SNi: (629.36) C, 64.88; H, 4.16; N, 8.90; S, 5.09. Found: C, 64.89; H, 4.34; N, 8.85; S, 4.73%. IR (KBr, cm^−1^): 1614 (s), 1535 (m), 1474 (m), 1444 (m), 1396 (m), 1362 (m), 1337 (m), 1305 (m), 1248 (m), 1136 (m), 1087 (m), 943 (m), 823 (m), 760 (m), 738 (m). MS (FAB) *m*/*z*: 628 [NiL(2,2′-bpy)], 472 [NiL], 317 [NiL-Ts]. UV-vis (cm^−1^): 7720, 11,100, 15,500, 21,180, 28,900.

[Ni_2_L_2_(4,4′-bpy)]. Electrolysis of H_2_L (0.10 g, 0.231 mmol) and 4,4′-bipyridine (0.037 g, 0.237 mmol) in acetonitrile for 1.3 h at 15 V and 10 mA dissolved 13.3 mg of nickel (Ef = 0.47 mol F^−1^). Via slow evaporation of the resulting red solution at room temperature, after a few days, orange crystals of [Ni_2_L_2_(4,4′-bpy)]. H_2_O suitable for X-ray studies were isolated. Yield: 0.0944 g (0.0843 mmol, 72%). Anal. Calc. for C_58_H_46_N_6_O_7_S_2_Ni_2_: (1120.54) C, 62.17; H, 4.14; N, 7.50; S, 5.72. Found: C, 62.56; H, 3.95; N, 8.12; S, 5.33%. IR (KBr, cm^−1^): 1613 (s), 1534 (m), 1482 (m), 1453 (m), 1343 (m), 1306 (m), 1214 (m), 1147 (m), 1120 (m), 1087 (m), 1038 (m), 989 (m), 950 (m), 811 (m), 749 (m). MS (FAB) *m*/*z*: 945 [Ni_2_L_2_], 628 [NiL(4,4′-bpy)], 472 [NiL], 317 [NiL-Ts]. UV-vis (cm^−1^): 11,960, 20,750, 2870. ^1^H NMR (DMSO-d6, ppm): 8.91 (s, 1H, CH=N), 8.22–7.48 (m, 10H, phenyl), 2.27 (s, 3H, CH_3_), 8.37 (d, 4H, 4,4′-bpy-H3,5), 7.75 (m, 4H, 4,4′-bpy-H2,6).

[NiLphen]. Electrochemical oxidation of a nickel anode in a solution of H_2_L (0.10 g, 0.231 mmol), and 1,10-phentrholine (0.047 g, 0.240 mmol) in acetonitrile, at 16 V and 10 mA for 1.3 h caused 13.5 mg of nickel to be dissolved; Ef = 0.47 mol F^−1^. After electrolysis, the red solution was allowed to stand in air at room temperature, and a brown powder solid of [NiLphen]·H_2_O was produced. Yield: 0.1286 g (0.1917 mmol, 83%). Anal. Calc. for C_36_H_28_N_4_O_4_SNi: (671.34) C, 64.40; H, 4.20; N, 8.34; S, 4.77. Found: C, 64.48; H, 4.60; N, 8.06; S, 4.24%. IR (KBr, cm^−1^): 1603 (s), 1538 (m), 1508 (m), 1456 (m), 1399 (m), 1361 (m), 1344 (m), 1305 (m), 1252 (m), 1181 (m), 1136 (m), 1088 (s), 973 (m), 857 (m), 824 (m),747 (m). MS (FAB) *m*/*z*: 652 [NiL(phen)], 499 [NiL(phen)-Ts], 472 [NiL]. UV-vis (cm^−1^): 7810, 11,400, 15,670, 20,490, 22,320, 30,300.

[ZnL(CH_3_OH)]_2_. In the case of zinc, electrolysis of 0.10 g (0.231 mmol) of H_2_L in acetonitrile for 1.3 h at 13 V and 10 mA resulted in the dissolution of 14.9 mg of zinc (Ef = 0.47 mol F^−1^). The brown microcrystalline product which was precipitated via evaporation at room temperature was collected and recrystallized from methanol to produce yellow crystals suitable for X-ray diffraction studies. These were isolated and identified as [ZnL(CH_3_OH)]_2_. Yield: 0.0781 g (0.1525 mmol, 66%). Anal. Calc. for C_26_H_22_N_2_O_4_SZn: (511.91) C, 58.65; H, 4.33; N, 5.47; S, 6.26. Found: C, 58.60; H, 4.41; N, 5.41; S, 6.08%. IR (KBr, cm^−1^): 3373 (m), 1617 (s), 1538 (s), 1480 (m), 1455 (m), 1399 (m), 1362 (m), 1342 (m), 1305 (m), 1252 (m), 1134 (s), 1088 (m), 974 (m), 823 (m), 747 (m). MS (FAB) *m*/*z*: 960 [Zn_2_L_2_], 480 [ZnL]. ^1^H NMR (DMSO-d6, ppm): 9.54 (s, 1H, CH=N), 8.25–6.70 (m, 10H, phenyl), 2.28 (s, 3H, CH_3_).

[ZnL(2,2′-bpy)]·CH_3_CN. The electrolysis of 0.10 g (0.231 mmol) g of H_2_L and 0.037 g (0.237 mmol) of 2,2′-bipyridine in acetonitrile for 1.3 h at 13 V and 10 mA resulted in the dissolution of 15.2 mg of zinc (Ef = 0.48 mol F^−1^). Air concentration of the resulting solution yielded crystals of [ZnL(2,2′-bpy)]·CH_3_CN suitable for single-crystal X-ray diffraction studies. Yield: 0.1360 g (0.2010 mmol, 87%). Anal. Calc. for C_36_H_29_N_5_O_3_SZn: (677.1) C, 63.86; H, 4.32; N, 10.34; S, 4.73. Found: C, 63.45; H, 4.36; N, 9.99; S, 4.72%. IR (KBr, cm^−1^): 2184 (m), 1617 (s), 1535 (m), 1475 (m), 1453 (m), 1390 (m), 1349 (m), 1307 (m), 1256 (s), 1136 (m), 1110 (m), 1088 (m), 981 (m), 830 (m), 775 (m), 765 (m), 755 (m), 734 (m). MS (FAB) *m*/*z*: 635 [ZnL(2,2′-bpy)], 479 [ZnL], 417 L. ^1^H NMR (DMSO-d6, ppm): 9.57 (s, 1H, CH=N), 7.67–6.67 (m, 10H, phenyl), 2.22 (s, 3H, CH_3_), 8.49 (d, 2H, 2,2′-bpy-H6,6′), 8.27 (d, 2H, 2,2′-bpy-H3,3′), 8.08 (m, 2H, 2,2′-bpy-H4,4′).

[ZnL(4,4′-bpy)]. A similar experiment (10 mA, 10V, 1.3 h) used 0.10 g (0.231 mmol) of H_2_L and 0.037 g (0.237 mmol) of 4,4′-bipyridine dissolved 15.0 mg of cadmium from the anode; Ef = 0.48 mol F^−1^. Concentration of the solution via slow evaporation produced a brown solid identified as [Zn_2_L_2_(4,4′-bpy)]. H_2_O. Yield: 0.0746 g (0.066 mmol, 57%). Anal. Calc. for C_58_H_46_N_6_O_7_S_2_Zn_2_: (1133.93) C, 61.43; H, 4.01; N, 7.41; S, 5.65. Found: C, 60.91; H, 4.06; N, 7.60; S, 5.52%. IR (KBr, cm^−1^): 3412 (m), 1614 (s), 1602 (m), 1537 (m), 1479 (m), 1455 (m), 1401 (m), 1362 (m), 1339 (m), 1303 (m), 1252 (s), 1218 (m), 1161 (m), 1133 (m), 1086 (m), 1019 (m), 973 (m), 828 (m), 812 (m), 747 (m). MS (FAB) *m*/*z*: 960 [Zn_2_L_2_], 635 [ZnL(4,4′-bpy)], 479 [ZnL], 417 L. ^1^H NMR (DMSO-d6, ppm): 9.55 (s, 1H, CH=N), 8.29–6.74 (m, 10H, phenyl), 2.27 (s, 3H, CH_3_), 8.71 (d, 4H, 4,4′-bpy-H3,5), 7.84 (m, 4H, 4,4′-bpy-H2,6).

[ZnLphen]. Electrolysis of an acetonitrile solution containing H_2_L (0.10 g, 0.231 mmol) and 0.047 g (0.240 mmol) of 1,10-phenantrholine at 10 mA and 13 V for 1.3 h dissolved 15.0 mg of zinc (Ef = 0.48 mol F^−1^). Slow evaporation of the brown solution at room temperature produced a brown solid product of [ZnLphen]. H_2_O CH_3_CN. Yield: 0.1312 g (0.1825 mmol, 79%). Anal. Calc. for C_38_H_31_N_5_O_4_SZn: (719.14) C, 63.46; H, 4.34; N, 9.74; S, 4.46. Found: C, 62.67; H, 4.50; N, 9.28; S, 3.973%. IR (KBr, cm^−1^): 3438 (m), 2181 (m), 1616 (m), 1584 (m), 1536 (m), 1519 (m), 1454 (m), 1392 (m), 1343 (m), 1306 (m), 1253 (m), 1138 (m), 1088 (s), 972 (m), 845 (m), 830 (m),750 (m), 726 (m). MS (FAB) *m*/*z*: 658 [ZnL(phen)], 503 [ZnL(phen)-Ts], 478, [ZnL]. ^1^H NMR (DMSO-d6, ppm): 9.60 (s,1H, CH=N), 8.30–6.40 (m, 10H, phenyl), 2.08 (s, 3H, CH3), 8.94 (d,2H, phen-H2,9), 8.74 (m, 2H, phen-H4,7), 8.15 (s,2H, phen-H5,6).

[CdL(2,2′-bpy)]. The oxidation of cadmium in a solution of acetonitrile containing 0.10 g (0.231 mmol) of H_2_L and 0.037 g (0.237 mmol) of 2,2′-bipyridine in acetonitrile (1.3 h at 12 V and 10 mA) dissolved 26.7 mg of cadmium (Ef = 0.49 mol F^−1^). Concentration of the solution via slow evaporation produced a yellow solid identified as [CdL(2,2′-bpy)]. Yield: 0.1214 g (0.1779 mmol, 77%). Anal. Calc. for C_34_H_26_N_4_O_3_SCd: (683.07) C, 59.78; H, 3.84; N, 8.20; S, 4.69. Found: C, 59.11; H, 3.76; N, 8.07; S, 4.84%. IR (KBr, cm^−1^): 1616 (m), 1535 (m), 1477 (m), 1446 (m), 1396 (m), 1339 (m), 1299 (m), 1258 (s), 1164 (m), 1158 (m), 1133 (s), 1088 (s), 976 (m), 814 (m), 754 (m),742 (m). ^1^H NMR (DMSO-d6, ppm): 9.42 (s, 1H, CH=N), 8.17–6.62 (m, 10H, phenyl), 2.25 (s, 3H, CH3), 8.64 (d, 2H, 2,2′-bpy-H6,6′), 8.49 (d 2H, 2,2′-bpy-H3,3′), 8.06 (m, 2H, 2,2′-bpy-H4,4′).

[CdL(4,4′-bpy)]. A similar experiment (10 mA, 10 V, 1.3 h) using 0.10 g (0.231 mmol) of H_2_L and 0.037 g (0.237 mmol) of 4,4′-bipyridine dissolved 25.6 mg of cadmium from the anode; Ef = 0.48 mol F^−1^. The brown solid compound obtained via slow evaporation of the resulting brown–green solution at room temperature was characterized as [Cd_2_L_2_(4,4′-bpy)]. H_2_O. Yield: 0.1092 g (0.089 mmol, 77%). Anal. Calc. for C_58_H_46_N_6_O_7_S_2_Cd_2_: (1227.98) C, 56.73; H, 3.78; N, 6.84; S, 5.22. Found: C, 62.49; H, 3.49; N, 6.71; S, 5.02%. IR (KBr, cm^−1^): 3448 (m), 1613 (m), 1602 (m), 1535 (m), 1479 (m), 1455 (m), 1426 (m), 1399 (m), 1340 (m), 1301 (m), 1257 (m), 1130 (m), 1088 (s), 1008 (m), 980 (m), 845 (m), 824 (m), 809 (m), 744 (m). ^1^H NMR (DMSO-d6, ppm): 9.36 (s, 1H, CH=N), 8.16–6.70 (m, 10H, phenyl), 2.29 (s, 3H, CH_3_), 8.77 (d, 4H, 4,4′-bpy-H3,5), 7.84 (m, 4H, 4,4′-bpy-H2,6).

[CdLphen]. The oxidation of cadmium in the presence of 1,10 phenanthroline (0.047 g (0.240 mmol) and H_2_L (0.10 g, 0.231 mmol) for 1.3 h at 12 V and 10 mA dissolved 27.5 mg of cadmium (Ef = 0.50 mol F^−1^). By slowly evaporating the resulting orange solution at room temperature, an orange powder solid of [CdLphen] was isolated. Anal. Calc. for C_36_H_26_N_4_O_3_SCd: (707.09) C, 61.15; H, 3.71; N, 7.92; S, 4.53. Found: C, 60.71; H, 3.66; N, 7.92; S, 54.28%. IR (KBr, cm^−1^): 3448 (m), 1613s (m), 1598 (m), 1578 (m), 1534 (m), 1515 (m), 1475 (m), 1366 (m), 1297 (m), 1256 (s), 1135 (s), 1088 (s), 975 (m), 844 (m), 814 (m), 749 (m), 728 (m). MS (FAB) *m*/*z*: 708, [CdL(phen)], 551 [CdL(phen)-Ts], 526 [CdL]. ^1^H NMR (DMSO-d6, ppm): 9.57 (s, ^1^H, CH=N), 8.00–6.20 (m, 10H, phenyl), 2.15 (s, 3H, CH_3_), 8.96 (d, 2H, phen-H2,9), 8.70 (m, 2H, phen-H4,7), 8.12 (s, 2H, phen-H5,6).

### 4.3. Instruments

Microanalyses were performed using a PerkinElmer 240B microanalyzer (Shelton, CT, USA). IR spectra were recorded as KBr mulls (spectroscopy grade, Merck, Darmstadt, Germany) on a Bruker IFS-66 V spectrophotometer (Billerica, MA, USA). The ^1^H spectrum of the ligand was recorded on a Bruker WM 350 MHz spectrophotometer (Billerica, MA, USA) using the DMSO-d6 solvent. IE and FAB mass spectra were recorded on Hewlet-Packard HP5988A (Palo Alto, CA, USA) and Micromass Autospec instruments (Milford, MA, USA), using 3-nitrobenzyl alcohol (3-NBA) (spectroscopy grade, Merck) as the matrix material for the FAB spectra. Solid-state electronic spectra (UV-vis) were measured on UV-3101 PC Shimadzu spectrophotometer (Kyoto, Japan).

### 4.4. Crystal Structure Determinations

The crystallographic data were collected at CACTUS (Universidade de Santiago de Compostela) using a Bruker CCD SMART1000, or in a Bruker X8 Kappa with Mo-Kα radiation (λ = 0.71073 Å). The software APEX [66] was used for collecting frames of data, indexing reflections and determining lattice parameters; SAINT [66] was used for the integration of the intensity of reflections; and SADABS [66] was used for scaling and empirical absorption correction. The crystallographic treatment was performed using the program Oscail, version 4.7.1 [67], and was solved using the program SHELXT, version 2018/2 [68]. The structure was subsequently refined using a full-matrix least-squares methods based on F^2^ using the program SHELXL, version 2018/3 [69]. Non-hydrogen atoms were refined using anisotropic displacement parameters. Hydrogen atoms were included in idealized positions and refined using isotropic displacement parameters. In the case of the compounds NEt_4_[CoL_2_] and [Ni_2_L_2_(4,4′-bpy)], the program Squeeze provided by PLATON (version 60720) [70] was used to correct the reflection data for the diffuse scattering due to a disordered unknown solvent. Unfortunately, these structures maintain high electronic densities in the final map. However, for the compound NEt_4_[CoL_2_], the low quality of data [*R*_int_ = 0.2063] did not allow us to improve the model to satisfactory final indices. For this reason, the geometrical parameters discussed for this compound should be read with appropriate caution. Other details concerning crystal data and structural refinement are given in Table 4, Table 5 and Table 6. CCDC 2467583-2467588 contain the supplementary crystallographic data for this paper. These data can be obtained free of charge from the Cambridge Crystallographic Data Centre via www.ccdc.cam.ac.uk/structures (accessed on 25 August 2025). PLATON (version 60720) [70] was used to obtain some geometrical parameters from the .cif files.

### 4.5. Hirshfeld Surface Analysis

The Hirshfeld surfaces were calculated using the Crystal Explorer v.3.1 program package and the 2D fingerprint was prepared using the same software [71]. This technique allows us to visualize the interactions between crystal structures [72,73]. The method uses visual recognition of several properties of intermolecular interactions by mapping them onto a surface (curvedness, shape index, *d_norm_*, etc.). Surfaces were drawn in this paper using the representation of the normalized contact distance, *d_norm_*, defined as the sum of the normalized by the van der Waals radius of the atoms involved, *d_i_* and *d_e_* [74]. *d_i_* and *d_e_* are defined, respectively, as the distance from the Hirshfeld surface to the nearest nucleus outwards from the surface and the distance from the surface to the nearest atom in the molecule itself. In this paper, the representations of such surfaces were shown in two figures, with the second one rotated 180° on the vertical axis, so the front and back were visible. They can be found in the Supplementary Materials. Finally, all (*d_i_*, *d_e_*) contacts can be expressed in the form of a two-dimensional plot, known as the 2D fingerprint plot [32,75,76]. The shape of this plot, which is unique for each molecule, is determined by dominating intermolecular contacts. The 2D fingerprint plots were constructed by using reciprocal interactions; that is, both X···Y and Y···X interactions were included in the fingerprints shown in the Supplementary Materials. The input file for calculations was the deposited .cif file for all of the compounds. And in this respect, it should be noted that compounds NEt_4_[CoL_2_] and [Ni_2_L_2_(4,4′-bpy)] crystallized with an unknown molecule of solvent (see Section 4), which is not included in the calculations, so their interactions were lost.

### 4.6. Electronic Structure Calculations

All of the structures were fully optimized using density functional theory within the Kohn–Sham framework [77,78]. The single-parameter hybrid PBE0 functional was employed along with the double-z 6-31G(d,p) basis set. A pruned grid with 175 radial shells and 974 angular points was used for numerical integration [79]. To avoid possible gas phase artifacts in these charged systems, the polarizable continuum method was applied (PCM) using parameters for acetonitrile [80]. Secondary derivatives were obtained for both the wavefunction, with respect to orbital rotations, and for the energy, with respect to atom displacements, to verify that wavefunction and molecular geometry are both minima in their respective hypersurfaces [81]. All of the simulations were performed using the Gaussian 16 suite of programs, except for the topological analysis of the electron density, which was performed using Multiwfn, and the non-covalent interactions analysis, which was performed using NCI [82,83,84].

## 5. Conclusions

The electrochemical oxidation of a metal anode (cobalt, nickel, zinc or cadmium) in a cell containing the potentially tridentate Schiff base ligand allowed us to synthesize neutral complexes with high purities and good yields. The presence in the electrochemical cell of additional bidentate nitrogen donors allows the synthesis of mixed complexes in one step. The highly pure isolated compounds were characterized using spectroscopic methods and, when possible, using single crystal X-ray diffraction. In all cases, the Schiff base acted as a dianionic tridentate ligand. X-ray analysis of the crystal structures along with DFT simulations and non-covalent interactions analysis revealed some interesting structural features regarding the intramolecular interactions. In particular, a metal-dependent reversal of bond lengths between the metal center and the amidate/imine nitrogen atoms of the tridentate ligand was described. Full characterization of the supramolecular structure was also provided via the analysis of the Hirshfeld surfaces.

## Figures and Tables

**Table 1:** Selected bond lengths (Å) and angles (°) for the anion [CoL_2_]^−^.

Co-N(11)	1.967(7)	Co-N(12)	1.871(8)
Co-N(21)	1.973(8)	Co-N(22)	1.867(8)
Co-O(13)	1.876(6)	Co-O(23)	1.875(6)
N(11)-Co-N(12)	83.5(3)	N(22)-Co-N(21)	83.3(3)
N(12)-Co-O(23)	89.7(3)	N(12)-Co-O(13)	93.5(3)
N(22)-Co-O(23)	93.3(3)	N(22)-Co-O(13)	88.6(3)
N(22)-Co-N(11)	94.5(3)	O(23)-Co-N(11)	92.2(3)
O(13)-Co-O(23)	87.3(3)	O(13)-Co-N(21)	91.8(3)
N(12)-Co-N(21)	93.7(3)	N(11)-Co-N(21)	88.8(3)
O(13)-Co-N(11)	176.9(3)	O(23)-Co-N(21)	176.5(3)
N(22)-Co-N(12)	176.4(3)		

**Table 2:** Selected bond lengths [Å] and angles [°] for the nickel compounds.

	[NiL(H_2_O)]	[NiL(CH_3_CN)(H_2_O)]_2_	[Ni_2_L_2_(4,4′bpy)] Mol. 1	[Ni_2_L_2_(4,4′bpy)] Mol. 2
Ni-N(1)	1.8502(16)	1.9898(19)	1.897(4)	1.854(3)
Ni-N(2)	1.9083(16)	2.0810(19)	1.850(3)	1.904(4)
Ni-O(3)	1.8277(13)	2.0009(16)	1.811(4)	1.819(3)
Ni-O(1W)	1.9309(17)	2.0527(17)		
Ni-N(3)		2.099(2)		
Ni-O(2 ^i^)		2.2069(16)		
Ni-N_4,4bpy_			1.922(4)	1.939(4)
Ni-Ni ^i^		5.4071(6)		
N(1)-Ni-N(2)	86.02(7)	81.05(8)	86.31(16)	86.11(16)
N(1)-Ni-O(3)	94.32(6)	89.02(7)	94.42(16)	94.14(15)
N(2)-Ni-O(3)	179.59(6)	169.45(7)	173.96(17)	173.85(17)
N(1)-Ni-O(1W)	173.32(7)	174.15(8)		
N(2)-Ni-O(1W)	95.96(7)	100.14(8)		
O(3)-Ni-O(1W)	83.73(7)	90.10(7)		
N(1)-Ni-N(3)		97.10(8)		
O(3)-Ni-N(3)		88.26(7)		
O(1W)-Ni-N(3)		88.65(8)		
N(2)-Ni-N(3)		89.44(8)		
N(1)-Ni-O(2 ^i^)		89.42(7)		
O(3)-Ni-O(2 ^i^)		90.04(6)		
O(1W)-Ni-O(2 ^i^)		84.80(7)		
N(2)-Ni-O(2 ^i^)		93.39(7)		
N(3)-Ni-O(2 ^i^)		173.23(7)		
N(1)-Ni-N_4,4′bpy_			92.33(16)	93.85(16)
N(2)-Ni-N_4,4′bpy_			170.76(17)	170.91(18)
O(3)-Ni-N_4,4′bpy_			87.89(15)	86.87(15)

Data were carefully ordered to show related bond distances or angles in the same row. Symmetry operations: i: 2−x, 1−y, 1−z.

**Table 3:** Selected bond lengths [Å] and bond angles [°] for the zinc compounds.

	[ZnL(MeOH)]_2_	[ZnL(2,2′-bpy)]
Zn-N(11)	2.045(2)	2.094(3)
Zn-N(12)	2.037(2)	2.040(4)
Zn-O(13)	2.0666(17)	1.953(3)
Zn-O(13 ^i^)	1.9951(17)	
Zn-O(1)	2.069(2)	
Zn-N(31)		2.118(3)
Zn-N(32)		2.117(3)
Zn-Zn ^i^	3.1572(13)	
N(12)-Zn-N(11)	80.79(8)	79.45(13)
N(11)-Zn-O(13)	84.98(8)	87.77(12)
N(12)-Zn-O(13)	165.57(7)	130.50(13)
N(11)-Zn-O(1)	125.78(8)	
N(12)-Zn-O(1)	95.33(8)	
O(13)-Zn-O(1)	91.01(7)	
N(11)-Zn-O(13 ^i^)	128.95(8)	
N(12)-Zn-O(13 ^i^)	113.05(8)	
O(13)-Zn-O(13 ^i^)	77.99(7)	
O(1)-Zn-O(13 ^i^)	102.50(8)	
N(1)-Zn-N(32)		169.61(13)
N(2)-Zn-N(32)		108.53(13)
O(3)-Zn-N(32)		91.67(12)
N(1)-Zn-N(31)		93.83(13)
N(2)-Zn-N(31)		105.57(13)
O(3)-Zn-N(31)		122.98(13)
N(31)-Zn-N(32)		77.78(13)

Symmetry operation, i, 1−x, −y, 1−z.

**Table 4:** Crystal data and structure refinement for NEt_4_[CoL_2_].

Identification code	NEt_4_[CoL_2_]
Empirical formula	C_56_H_56_CoN_5_O_6_S_2_
Moiety formula	C_56_H_56_CoN_5_O_6_S_2_
Formula weight	1018.1
Temperature	100(2) K
Wavelength	0.71073 Å
Crystal system	Monoclinic
Space group	*P*2_1_*/n*
Unit cell dimensions	a = 15.527(4) Å
	b = 20.322(5) Å
	c = 17.487(4) Å
	α = 90°
	β = 106.295(4)°
	γ = 90°
Volume	5296(2) Å^3^
Z	4
Density (calculated)	1.277 Mg/m^3^
Absorption coefficient	0.457 mm^−1^
F(000)	2136
Crystal size	0.220 × 0.210 × 0.070 mm^3^
Theta range for data collection	1.552 to 26.451°
Index ranges	−19 ≤ *h* ≤ 19
	−25 ≤ *k* ≤ 25
	−21 ≤ *l* ≤ 21
Reflections collected	44,312
Independent reflections	10,884 [*R*_int_ = 0.2063]
Reflections observed (>2σ)	4070
Data completeness	0.995
Absorption correction	Semi-empirical from equivalents
Max. and min. transmission	0.9593 and 0.2509
Refinement method	Full-matrix least-squares on F^2^
Data/restraints/parameters	10,884/0/634
Goodness-of-fit on *F*^2^	1.018
Final *R* indices [*I* > 2σ(*I*)]	*R*_1_ = 0.1115
	w*R*_2_ = 0.2644
*R* indices (all data)	*R*_1_ = 0.2548
	w*R*_2_ = 0.3805
Largest diff. peak and hole	1.045 and −1.026 e·Å^−3^

**Table 5:** Crystal data and structure refinement for nickel compounds.

Identification code	[NiL(H_2_O)]	[NiLCH_3_CN)(H_2_O)]_2_	[Ni_2_L_2_(4,4′bpy)]
Empirical formula	C_24_H_20_N_2_NiO_4_S	C_26_H_23_N_3_NiO_4_S	C_29_H_22_N_3_NiO_3_S
Moiety formula	C_24_H_20_N_2_NiO_4_S	(C_26_H_23_N_3_NiO_4_S)_2_	C_29_H_22_N_3_NiO_3_S
Formula weight	491.19	532.24	551.26
Temperature	293(2) K	273(2) K	273(2) K
Wavelength	0.71073 Å	0.71073 Å	0.71073 Å
Crystal system	Triclinic	Triclinic	Triclinic
Space group	*P*−1	*P*−1	*P*−1
Unit cell dimensions	a = 8.2708(3) Å	a = 9.5918(2) Å	a = 12.9369(6) Å
	b = 11.0377(5) Å	b = 11.1199(2) Å	b = 15.1884(7) Å
	c = 12.0578(4) Å	c = 12.3547(2) Å	c = 16.3634(7) Å
	α = 73.900(2)°	α = 105.0580(10)°	α = 112.800(2)°
	β = 83.556(2)°	β = 90.7020(10)°	β = 106.758(3)°
	γ = 79.093(2)°	γ = 113.3570(10)°	γ = 98.396(3)°
Volume	1036.40(7) Å^3^	1158.31(4) Å^3^	2714.5(2) Å^3^
Z	2	2	4
Density (calculated)	1.574 Mg/m^3^	1.526 Mg/m^3^	1.349 Mg/m^3^
Absorption coefficient	1.073 mm^−1^	0.968 mm^−1^	0.826 mm^−1^
F(000)	508	552	1140
Crystal size	0.47 × 0.14 × 0.12 mm	0.13 × 0.08 × 0.04 mm	0.360 × 0.050 × 0.030 mm
Theta range for data collection	1.761 to 26.466°	1.721 to 26.427°	1.719 to 26.480°
Index ranges	−9 ≤ *h* ≤ 10	−12 ≤ *h* ≤ 11	−16 ≤ *h* ≤ 16
	−13 ≤ *k* ≤ 13	−13 ≤ *k* ≤ 13	−18 ≤ *k* ≤ 18
	−15 ≤ *l* ≤ 15	−15 ≤ *l* ≤ 15	−20 ≤ *l* ≤ 20
Reflections collected	14,160	19,189	43,941
Independent reflections	4251 [*R*_int_ = 0.0280]	4748 [*R*_int_ = 0.0445]	11,007 [*R*_int_ = 0.0968]
Reflections observed (>2σ)	3720	3611	5951
Data completeness	0.991	0.997	0.981
Absorption correction	Semi-empirical from equivalents	Semi-empirical from equivalents	Semi-empirical from equivalents
Max. and min. transmission	0.8821 and 0.6325	0.9623 and 0.8845	0.9756 and 0.7553
Refinement method	Full-matrix least-squares on *F*^2^	Full-matrix least-squares on *F*^2^	Full-matrix least-squares on *F*^2^
Data/restraints/parameters	4251/0/302	4748/0/326	11,007/0/669
Goodness-of-fit on *F*^2^	1.054	1.007	0.988
Final *R* indices [*I* > 2sigma(*I*)]	*R*_1_ = 0.0299	*R*_1_ = 0.0344	*R*_1_ = 0.0619
	w*R*_2_ = 0.0725	w*R*_2_ = 0.0692	w*R*_2_ = 0.1366
*R* indices (all data)	*R*_1_ = 0.0362	*R*_1_ = 0.0587	*R*_1_ = 0.1363
	w*R*_2_ = 0.0752	w*R*_2_ = 0.0770	w*R*_2_ = 0.1681
Largest diff. peak and hole	0.461 and −0.472 e·Å^−3^	0.420 and −0.436 e·Å^−3^	0.963 and −0.570 e·Å^−3^

**Table 6:** Crystal data and structure refinement for zinc compounds.

Identification code	[ZnL(MeOH)]_2_	[ZnL(2,2′-bpy)]
Empirical formula	C_50_H_44_N_4_O_8_S_2_Zn_2_	C_36_H_28_N_5_O_3_SZn
Moiety formula	C_50_H_44_N_4_O_8_S_2_Zn_2_	C_36_H_28_N_5_O_3_SZn
Formula weight	1023.75	676.06
Temperature	120(2) K	273(2) K
Wavelength	0.71069 Å	0.71073 Å
Crystal system	Triclinic	Monoclinic
Space group	*P*−1	*P*2_1_/*c*
Unit cell dimensions	a = 7.812(5) Å	a = 16.378(3) Å
	b = 11.982(5) Å	b = 10.6843(17) Å
	c = 13.282(5) Å	c = 17.377(3) Å
	α = 72.254(5)°	α = 90°
	β = 73.749(5)°	β = 91.478(6)°
	γ = 71.882(5)°	γ = 90°
Volume	1101.6(9) Å^3^	3039.9(9) Å^3^
Z	1	4
Density (calculated)	1.543 Mg/m^3^	1.477 Mg/m^3^
Absorption coefficient	1.246 mm^−1^	0.924 mm^−1^
F(000)	528	1396
Crystal size	0.440 × 0.130 × 0.110 mm	0.150 × 0.140 × 0.050 mm
Theta range for data collection	1.644 to 27.173°	2.238 to 26.401°
Index ranges	−10 ≤ *h* ≤ 10	−20 ≤ *h* ≤ 20
	−15 ≤ *k* ≤ 15	−12 ≤ *k* ≤ 13
	−17 ≤ l ≤ 17	−20 ≤ *l* ≤ 21
Reflections collected	13,165	25,355
Independent reflections	4851 [*R*_int_ = 0.0358]	6213 [*R*_int_ = 0.0934]
Reflections observed (>2σ)	3910	3843
Data completeness	0.991	0.995
Absorption correction	Semi-empirical from equivalents	Semi-empirical from equivalents
Max. and min. transmission	1.0000 and 0.730982	0.7449 and 0.4653
Refinement method	Full-matrix least-squares on F^2^	Full-matrix least-squares on *F*^2^
Data/restraints/parameters	4851/0/304	6213/0/417
Goodness-of-fit on *F*^2^	1.064	1.023
Final *R* indices [*I* > 2σ(*I*)]	*R*_1_ = 0.0339	*R*_1_ = 0.0494
	w*R*_2_ = 0.0799	w*R*_2_ = 0.0944
*R* indices (all data)	*R*_1_ = 0.0508	*R*_1_ = 0.1113
	w*R*_2_ = 0.0887	w*R*_2_ = 0.1206
Largest diff. peak and hole	0.456 and −0.625 e·Å^−3^	0.712 and −0.994 e·Å^−3^

## Data Availability

The original contributions presented in this study are included in the article/Supplementary Materials. Further inquiries can be directed to the corresponding authors.

## Supplementary Materials

The following supporting information can be downloaded at: https://www.mdpi.com/article/10.3390/molecules30173543/s1, Critical Points and density analysis; Cartesian coordinates of the stationary points computed; IR Spectra, ^1^H RMN Spectra.
