# Supplementary material for: Coordinative Behavior of a New Hydroxynaphthanyl Sulphonamide Tridentate Schiff Base Towards First Row Late Transition Metal (LTM) and Post-Transitional Metal Atoms Zn and Cd: A Crystallographic and Computational Study
Source: Molecules. 2025 Aug 29;30(17):3543. doi: 10.3390/molecules30173543 (PMC12430383; doi:10.3390/molecules30173543)
Supplement: Supplementary file 1 [file molecules-30-03543-s001.zip › molecules-3754295-supplementary.pdf]

Coordinative behaviour of a new hydroxynaphthanyl sulphonamide tridentate Schiff base towards first row late transition metal (LTM) and post-transitional metal atoms Zn and Cd: A crystallographic and computational study.

Laura Sánchez-Guirao[1], Joaquín Viqueira[2], Carlos Silva López[1], José A. García-Vázquez[2], Jesús Castro[3*]

[1] Departamento de Química Orgánica, Universidad de Vigo, 36310 Pontevedra, Galicia, Spain, csilval@uvigo.gal (C.S.); lasanchez@uvigo.gal (L.S.G)

[2] Departamento de Química Inorgánica, Universidad de Santiago de Compostela, 15782 Santiago de Compostela, Galicia, Spain; joaquinangel.viqueira@gmail.com (J.V); josearturo.garcia@usc.es (J.A.G.-V.)

[3] Departamento de Química Inorgánica, Universidad de Vigo, 36310 Pontevedra, Galicia, Spain; jesusc@uvigo.gal

* Correspondence: jesusc@uvigo.gal, csilval@uvigo.gal

Contents

1 Supramolecular and Hirshfeld Surface analysis 2

1.1.1 Supramolecular structure of $NEt_4[CoL_2]$ **(1)** 2

1.1.2 Hirshfeld surface analysis for $NEt_4[CoL_2]$ **(1)** 3

1.2.1 Supremolecular structure of $[NiL(H_2O)]$ **(2)**. 6

1.2.2. Hirshfeld surface analysis of $[NiL(H_2O)]$ (2). 7

1.3.1 Supramolecular structure of $[NiL(CH_3CN)(H_2O)]_2$ **(3)**. 9

1.3.2. Hirshfeld surface analysis of $[NiL(CH_3CN)(H_2O)]$ 10

1.4.1 Supramolecular structure of $[Ni_2L_2(4,4'\text{-}bpy)]$ **(4)**. 11

1.4.2. Hirshfeld surface analysis of $[NiL(4,4'\text{-}bpy)]$. 12

1.5.1 Supramolecular structure of $[Zn_2L_2(MeOH)_2]$ **(5)**. 13

1.5.2 Hirshfeld surface analysis of $[ZnL(MeOH)]_2$ **(5)** 15

1.6.1 Supramolecular structure of $[ZnL(2,2'\text{-}bpy)]\cdot CH_3CN$ **(6).** 15

1.6.2 Hirshfeld surface analysis for $[ZnL(2,2'\text{-}bpy)]$ 17

2 Critical Points and density analysis 19

3 Cartesian coordinates of the stationary points computed 21

4 IR Spectra 36

5 ^{1}H NMR Spectra 52

1 Supramolecular and Hirshfeld Surface analysis

1.1.1 Supramolecular structure of $NEt_4[CoL_2]$ ***(1)***

Data for the supramolecular structure of 1 is summarized in Tables S1 and S2. In the first place, some intramolecular C-H···O interactions between several aromatic C-H groups and both the naphthol and the sulphonyl oxygen atoms are established. Key intermolecular C-H···O interactions are formed due to the presence of tetraethylamonium cations in the crystal lattice, resulting in a linear growing parallel to the zone axis (1, 0, -1).

Table S1.- Hydrogen bond parameters (Å and °) for $NEt_4[CoL_2]$.

Donor-H···Aceptor	D-H	H···A	D···A	D-H···A (°)
C(1)-H(1A)...O(11)	0.99	2.28	3.186(15)	152.6
C(12)-H(12)...O(23)	0.95	2.28	3.178(10)	157.9
C(19)-H(19)...O(12)	0.95	2.32	2.999(9)	127.8
C(15)-H(15)...O(12)	0.95	2.51	2.895(11)	104.1
C(5)-H(5B)...O(21^i)	0.99	2.75	3.106(17)	101.5
C(5)-H(5B)...O(22^i)	0.99	2.54	3.197(19)	123.3

Symmetry operation, i: x+0.5, 1.5-y, z-0.5

The framework is constructed by using the ethyl groups of the cation and the sulphonamide oxygen atoms of the coordinated Schiff base. In this chain some C-H···π interactions are also found as can be seen in Figure S1. These chains are connected with other parallel chains through two new C-H···π interactions, one of them using the tetraethylamonium cation and the other one using a C–H group of a tolyl ring (labelled as C(23)). These interactions produce a sheet growing parallel to the zone axis (1, 0, 1).

Table S2.- Geometrical parameters of C-H···π interactions for $NEt_4[CoL_2]$.

C-H···π	H···Cg[a]	C···Cg	C-H···Cg(°)
C(6)-H(6C)···Cg2	2.69	3.61(3)	157
C(3)-H(3B)···$Cg1^i$	2.91	3.867(15)	162
C(23)-H(23)··· $Cg3^{ii}$	2.73	3.660(10)	165

[a]Cg1: Centroid of the substituted ring of the naphthalene moiety. Cg2: Centroid of the tolyl ring. Cg3: Centroid of benzene in the naphtalene moiety. Symmetry operations, i: x+0.5, 1.5-y, z-0.5; ii: x+0.5, 1.5-y, z+0.5.

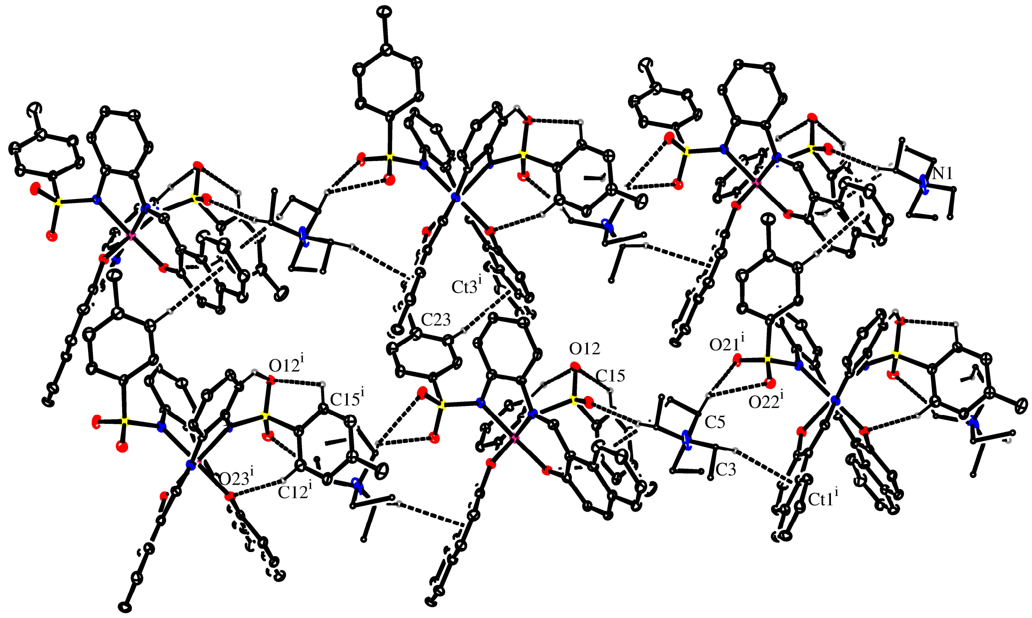

Figure S1. Supramolecular network for $NEt_4[CoL_2]$ **(1)**

1.1.2 Hirshfeld surface analysis for $NEt_4[CoL_2]$ **(1)**

The study of the Hirshfeld surfaces allows the vision of the interactions in crystal structures (see the Experimental Section for more details). The technique allow for visual recognition of several properties of intermolecular interactions through mapping onto this surface (curvedness, shape index, d_{norm}, etc.). Additionally, all (d_i, d_e) contacts can be expressed in the form of a two dimensional plot, known as the 2D fingerprint plot.

The two independent molecules found in the asymmetric unit (anion and cation) were considered and studied as independent ones. Figure S2 shows the front and back Hirshfeld surface modeled on d_{norm} (rotated by 180° around the vertical axis of the plot) for both ions, displayed as semitransparent surfaces to allow the visualization of the molecules behind. These surfaces are intense in color in the proximity of the SO_2 groups due the non classical hydrogen bonds formed.

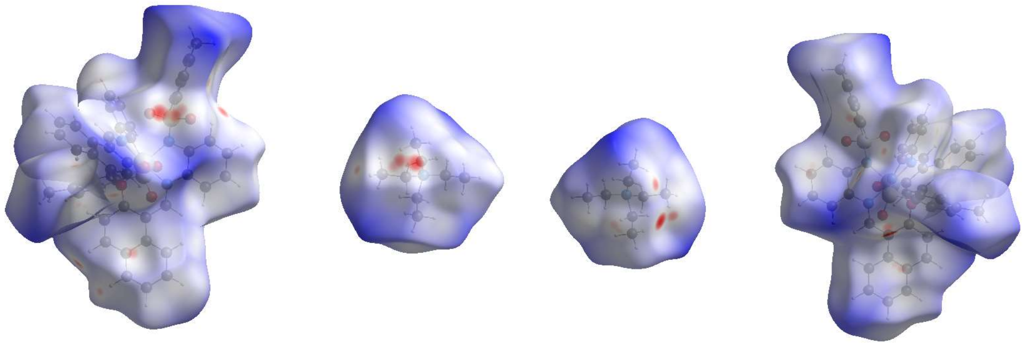

Figure S2. Front and back Hirshfeld surface modeled on d_{norm} for $NEt_4[CoL_2]$.

Figure S3 contains the d_{norm} and the d_i plot of the anionic complex with nearby cations because the most important interactions are produced between the anion and the cation. Note that the deepest red spots correspond with the position of the cations.

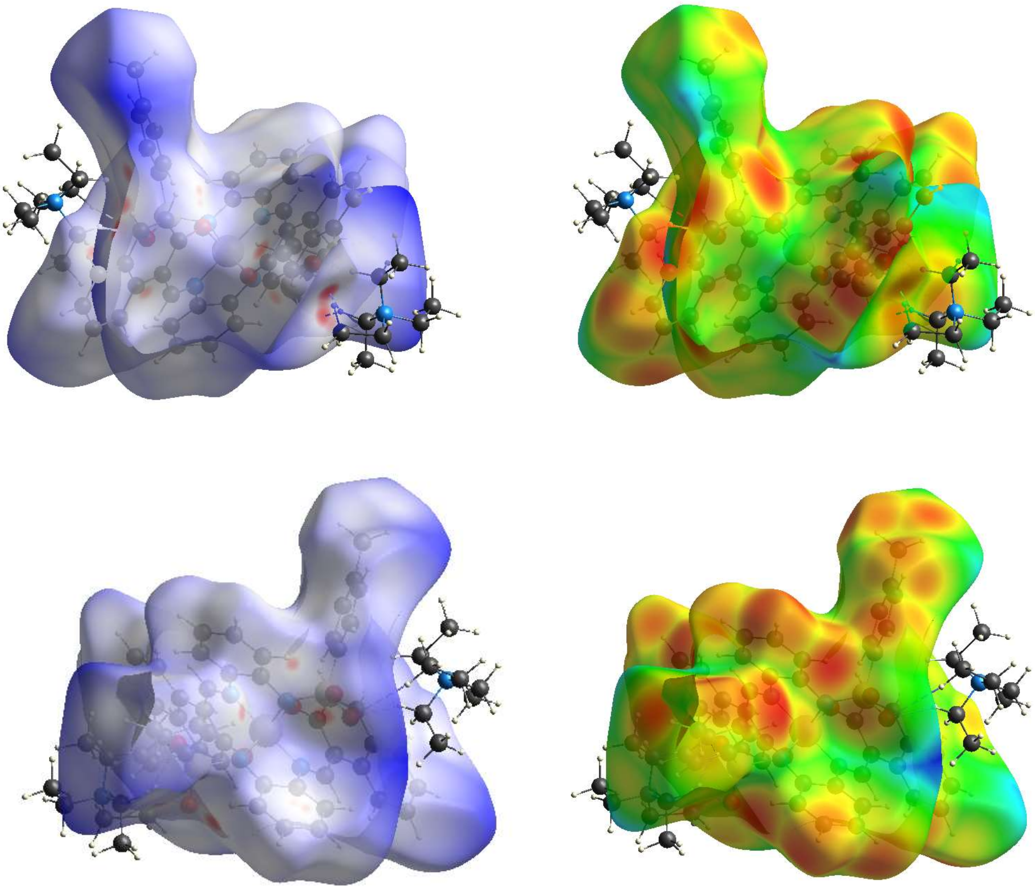

Figure S3. The d_{norm} and the d_i plot of the anionic complex in $NEt_4[CoL_2]$.

The above-mentioned 2D fingerprint plots were also studied separately (Figures S4 and S5). As can be seen, van der Waals forces (H···H contacts, 56.9% for the anionic complex and 56.8 % for the NEt_4^+ cation) constitute the majority of forces contributing to the supramolecular network. Contribution of the C···H and the O···H interactions are 28.6 and 10.9%, respectively and in both cases the asymmetry of the fingerprint is noteworthy. In latter, the shorter interactions correspond with around 0.9 + 1.3 Å, in close agreement with values in Tables S1 and S2. The fingerprint of the NEt_4^+ cation is also quite interesting, since it represents the interaction of this cation with the anionic complex. Two strongly asymmetric spikes can be observed in these fingerprints, one of them due to the H···H interactions with the anion, distances around 1+1 Å, and the other due to the O···H interactions, also 0.9 + 1.3 Å.

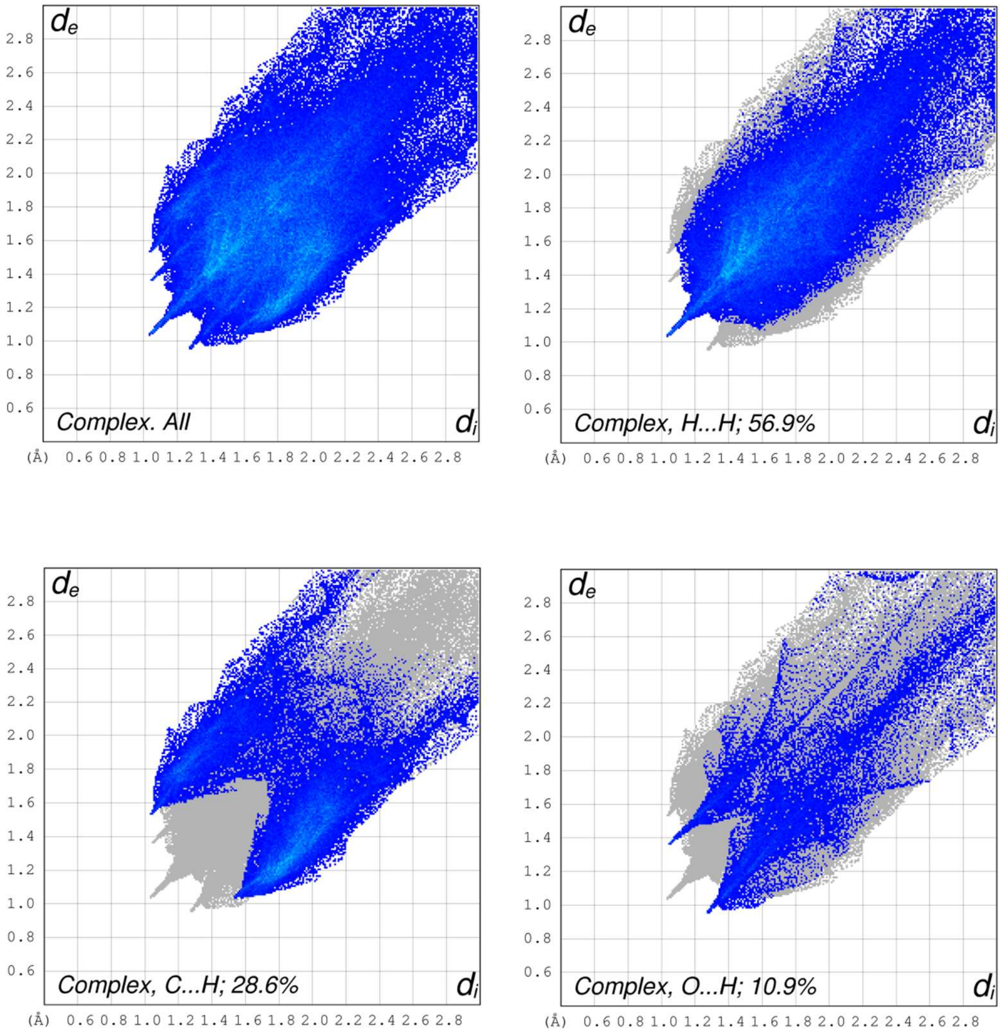

Figure S4. The 2D fingerprint plots of the anion complex in *$NEt_4[CoL_2]$*.

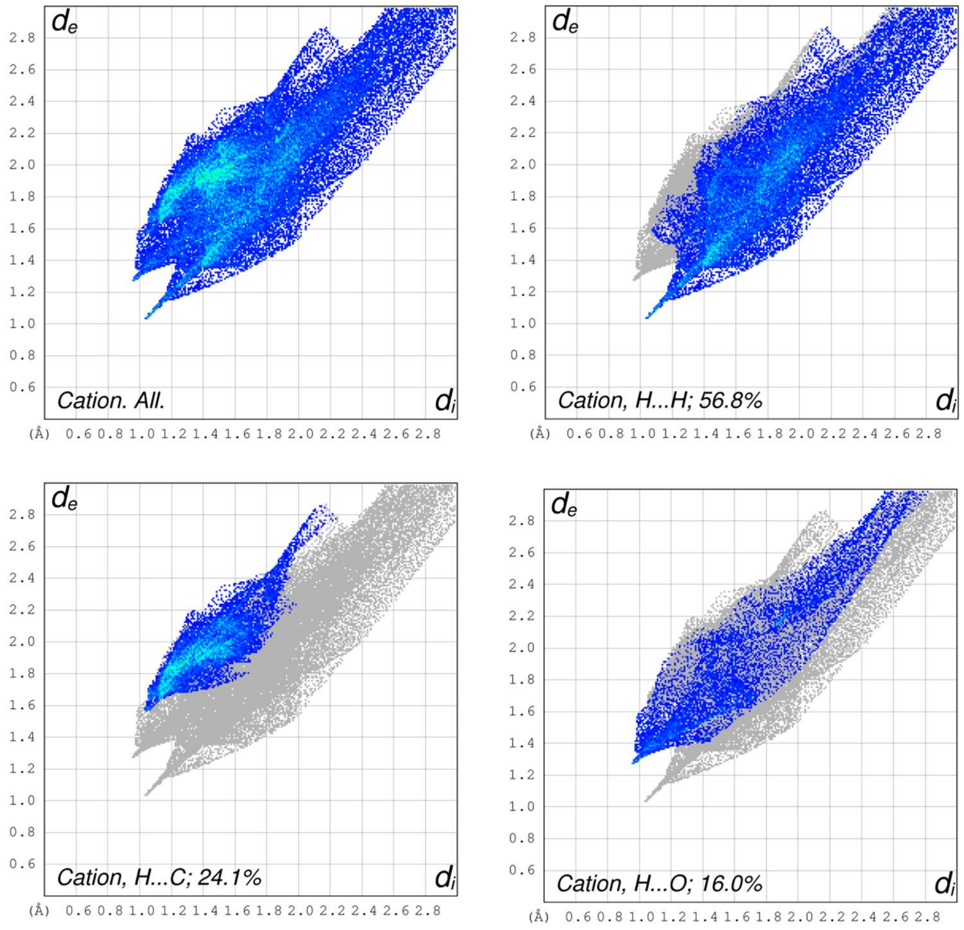

Figure S5. The 2D fingerprint plots of the cation in $NEt_4[CoL_2]$.

1.2.1 Supramolecular structure of [NiL(H_2O)] **(2)**.

Dealing with the supramolecular structure of complex **2** (Figure S6), the presence of coordinated water allows for some hydrogen bonds between water and the oxygen atom of a neighbour sulphonyl group. It is worth noting that the hydrogen atoms on water are disordered in such a way that three positions for them are found with an occupancy factor or 2/3. Whichever position the hydrogen atom occupies, an interaction is established with several surrounding atoms. There are intramolecular interactions, as that of the oxygen phenolate atom with this coordinated water, and there are intermolecular interactions. These ones build a supramolecular zig-zag chain parallel to the c axis. They are established between the water molecule and a SO_2 group of a neighbouring molecule (2-x, -y, 2-z) and also with the π-cloud of the benzene ring of the naphthol of a third molecule (2-x, -y, 1-z), in such a way that water molecules are the glue sticking the different complexes together into the observed chain. Parameters of these interactions are summarized in Table S3.

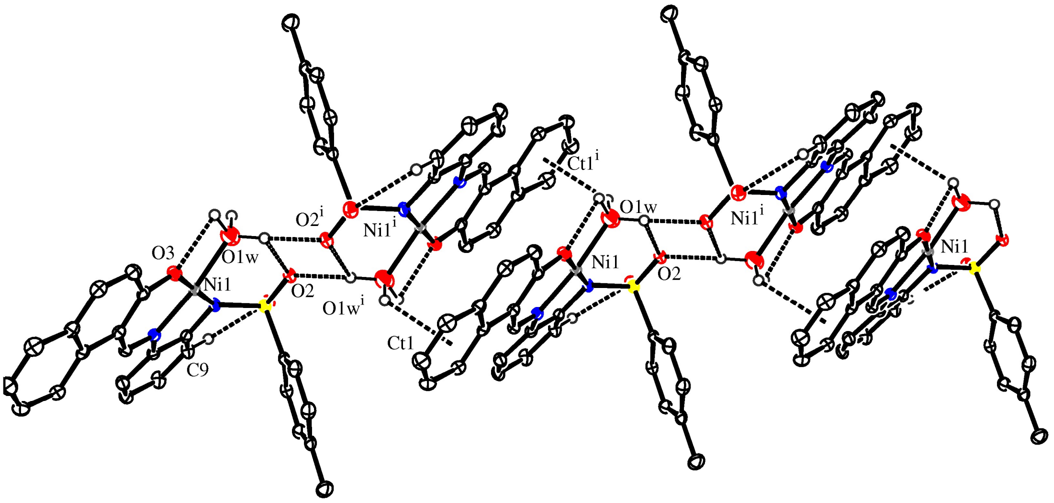

Figure S6. The supramolecular network in [NiL(H_2O)].

Table S3. Hydrogen bonds for [NiL(H_2O)] [Å and °.].

D-H...A	d(D-H)	d(H...A)	d(D...A)	D-H···A (°)
O(1W)-H(3W)...O(2^i)	1.07(3)	2.00(3)	3.064(2)	174(3)
O(1W)-H(2W)...O(3)	1.10(3)	2.22(3)	2.510(2)	91.9(19)
O(1W)-H(3W)...O(2)	1.07(3)	2.34(3)	2.675(2)	95.9(19)
O(1W)-H(1W)...CT1^{ii}	1.16(3)	2.55(3)	3.3329(19)	124(2)
C(5)-H(5)…O(1^{iii})	0.93	2.53	3.412(3)	159

Symmetry Opp.: i: 2-x, -y, 2-z; ii: 2-x, -y, 1-z; iii: x-1, y, z.

1.2.1. Hirshfeld surface analysis of [NiL(H_2O)] (2).

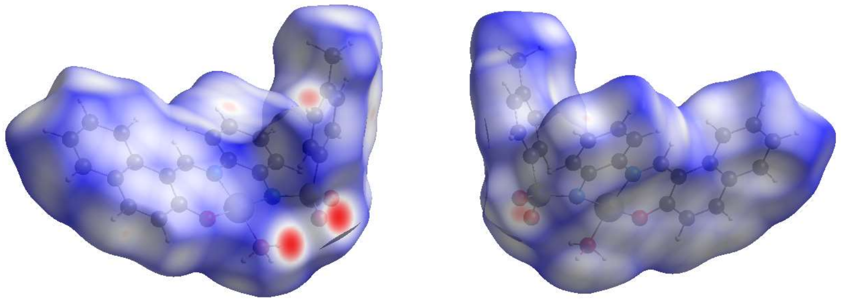

Figure S7. Front and back Hirshfeld surface modeled on d_{norm} for [NiL(H_2O)] **(2)**

Figure S7 shows the Hirshfeld surface for this compound showing a 180° rotation pair. In the figure on the left, two red spots can be seen, one of them corresponds with the position of the coordinated water molecule, which interacts with the sulfonyl group of the neighbor molecule, where the other red spot is visible. Figure S8 shows the mentioned two molecules.

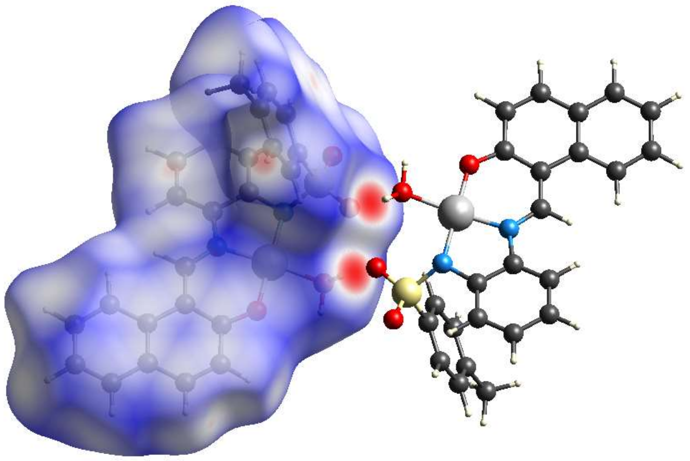

Figure S8 Hirshfeld surface modeled on d_{norm} for [NiL(H_2O)] and the nearest molecule.

The 2D fingerprints shown in Figure S9 indicate the importance of these interactions, with a weight of the O···H interactions up to 12.6%, although the closest distance is slightly bigger than 1.2+0.8 Å. Almost 50% of the interaction with neighbor molecules are H···H interactions, revealing a relatively low packing for the chair shape molecule.

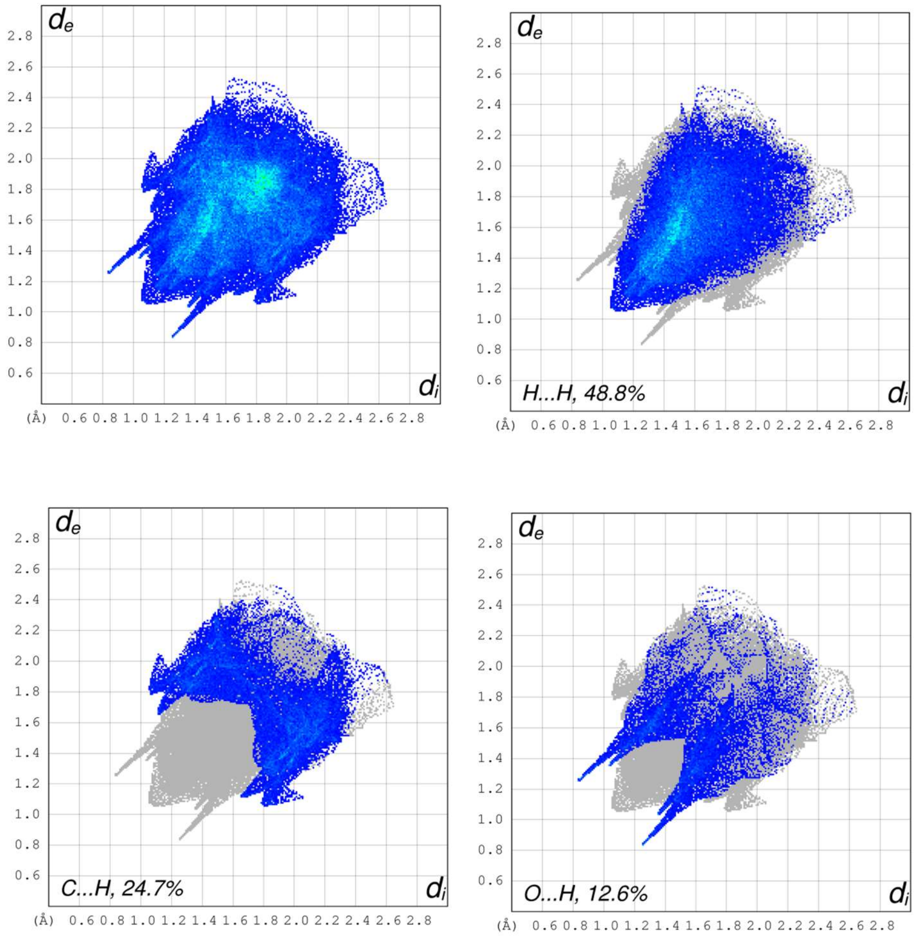

Figure S9. The 2D fingerprint plots of [NiL(H_2O)] **(2)**.

*1.3.1 Supramolecular structure of [NiL(CH_3CN)(H_2O)]$_2$ **(3)**.*

Once more the presence of coordinated water allows an interesting supramolecular structure via hydrogen bonds, and also some intramolecular interactions, one of them between the coordinated water and the SO_2 group within the same complex (see Table S4 and Figure S10). The intermolecular network is constructed connecting dimeric units with an intermolecular hydrogen bond in such a way than a linear chain parallel to the *a* axis is build. Additionally, the naphthalene moiety allows some π,π-stacking contacts in a "face to tail" mode. All the naphthalene moieties are involved in the stacking contacts so the dimeric units are connected in another chain parallel to the zone axis (1,0,1). The resulting supramolecular structure is a sheet in the *ac* plane.

Table S4. Hydrogen bond parameters for [NiL(CH_3CN)(H_2O)]$_2$.

Donor-H···Aceptor	D-H	H···A	D···A	D-H···A (°)
O(1W)-H(1W)...O(2)	0.74(3)	2.00(3)	2.698(3)	157(3)
O(1W)-H(2W)···O(3^{i})	0.81(3)	1.92(3)	2.726(3)	172(3)

Symmetry operation: i: 1-x, -y, -z

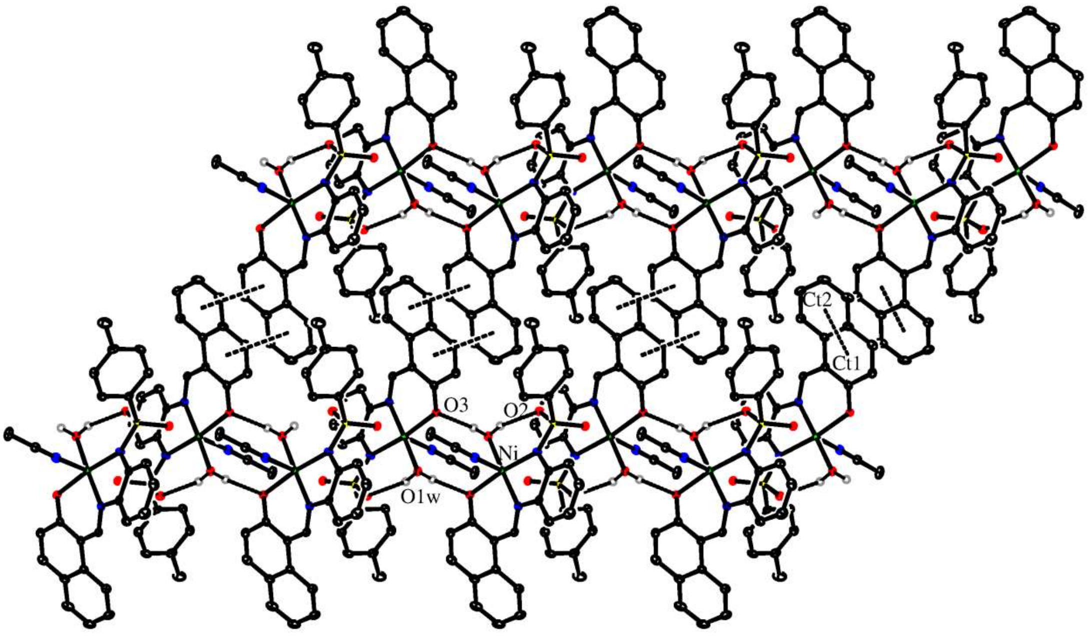

Figure S10. Supramolecular network in [NiL(CH_3CN)(H_2O)]$_2$ **(3)**.

1.3.2. Hirshfeld surface analysis of [NiL(CH_3CN)(H_2O)]

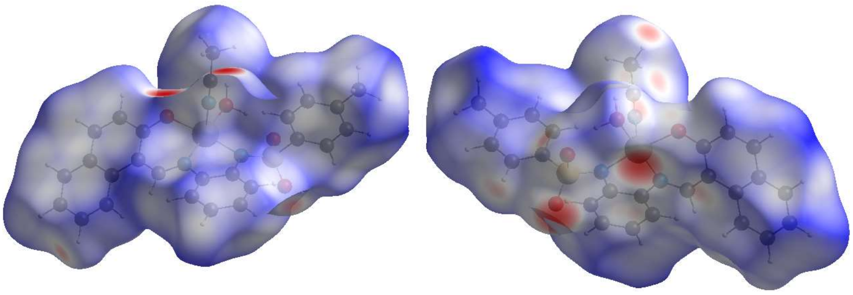

Figure S11. Front and back Hirshfeld surface modeled on d_{norm} for [NiL(CH_3CN)(H_2O)] **(3)**

Figure S11 shows the front and back Hirshfeld surface modeled on dnorm for [NiL(CH_3CN)(H_2O)]. The study was done on the asymmetric unit, pentacoordinated nickel(II) compound, and considering the Ni-O(2') bond of 2.2069(16) Å as an intermolecular one, since it is longer than the other five. The latter would be the main interaction with surroundings. The other interactions found are exactly the same for both symmetry related halves. Figure S12 shows the dimeric units (Hirshfeld surface just on one of the molecules). These O···H interactions are showed in the 2D fingerprints (Figure S13) as two small spikes, with distances of 1.1+0.7 Å.

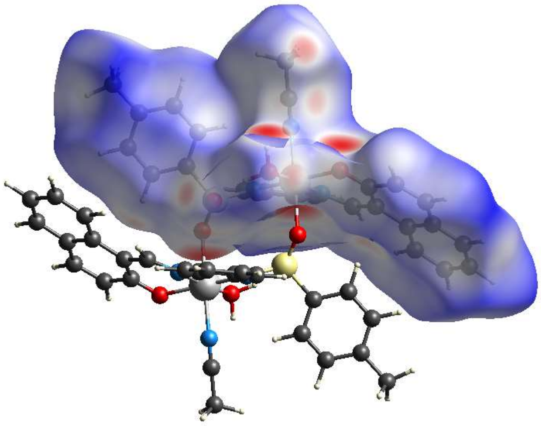

Figure S12. Hirshfeld surface modeled on d_{norm} for [NiL(CH_3CN)(H_2O)] **(3)** and the nearest molecule (below considered as part of the dimeric unit) .

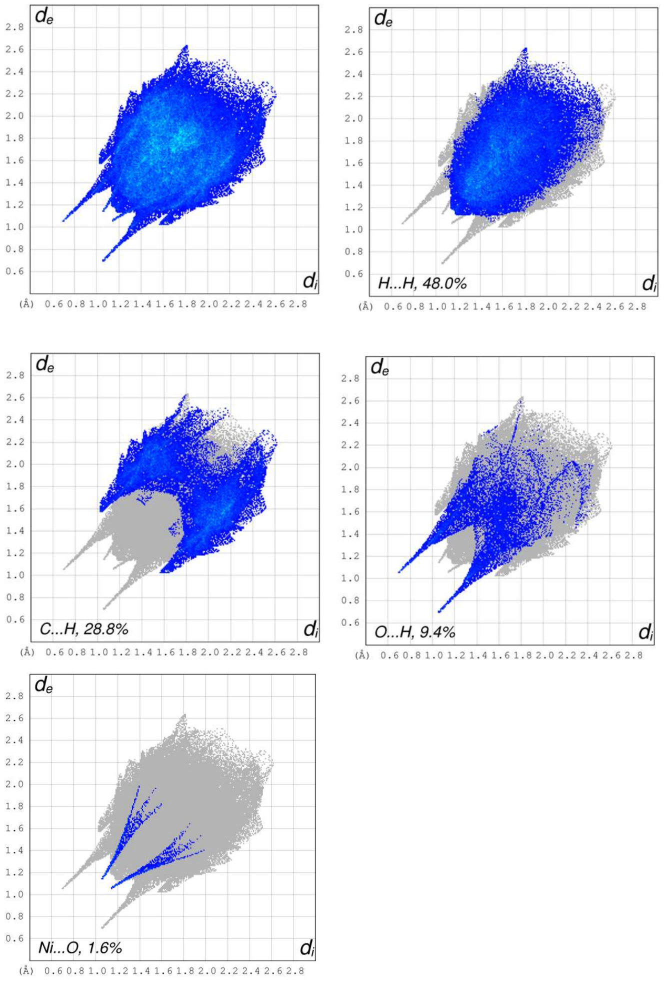

Figure S13 The 2D fingerprint plots of [NiL(CH_3CN)(H_2O)] **(3)**.

1.4.1 Supramolecular structure of [Ni_2L_2(4,4′-bpy)] ***(4)****.*

In terms of the supramolecular structure, the presence in the unit cell of two different molecules allows for some non-classical C-H···O interactions between aromatic rings and SO_2 groups as indicated in Table S5. These interactions connect the two molecules. Figure S14 shows the supramolecular growing along the zone axis (1,1,1) by means of those interactions. Additionally, the second oxygen atom in the SO_2 groups establish other C-H···O interactions, this time along the *a* axis.

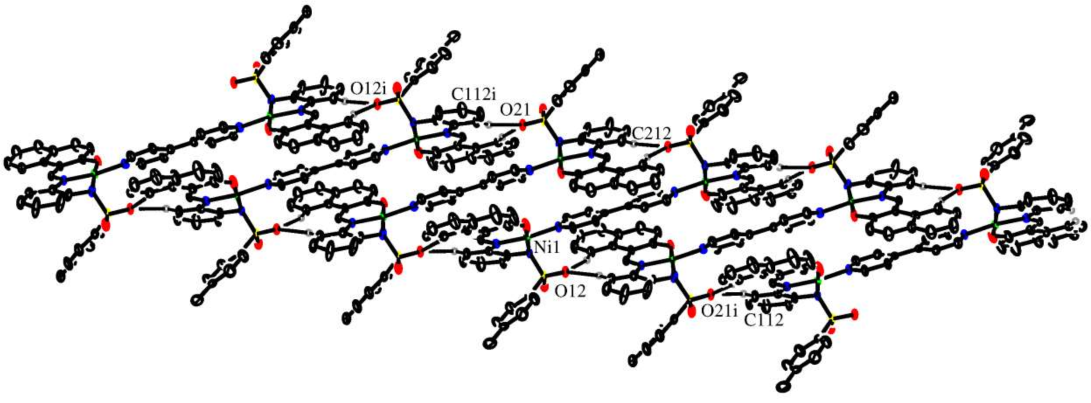

Figure S14. Growing of the supramolecular network in [NiL(4,4'-bpy)]

Table S5. Hydrogen bonds parameters in [NiL(4,4'-bpy)]
Symmetry operations: i: 1+x, 1+y, 1+z; ii: 1+x, y, z

Donor-H···Aceptor	D-H	H···A	D···A	D-H···A (º)
C(112)-H(112)···O(22)	0.93	2.28	3.191(7)	166
C(117)-H(117)···O(22)	0.93	2.56	3.469(7)	168
C(212)-H(212)···O(12^{i})	0.93	2.32	3.235(7)	168
C(217)-H(217)···O(12^{i})	0.93	2.50	3.394(6)	160
C(220)-H(220)···O(22^{ii})	0.93	2.50	3.385(7)	159

1.4.2. Hirshfeld surface analysis of [NiL(4,4′-bpy)].

Figures S15 and S16 show the front and back Hirshfeld surface modeled on d_{norm} for [NiL(4,4'-bpy)]. The study was done independently on the two dimers, that is, Figure S15 shows the Hirshfeld surface on modeled the natural growing (symm. opp. -x, -y, -z) of the one half of the asymmetric unit, and Figure S16 shows the Hirshfeld surface modeled on the natural growing (symm. opp. 1-x, 1-y, 1-z) of the other half. Both figures are quite similar but not exactly the same, as demonstrated by the different 2D fingerprints showed in Figure S17. The most intense red spots on the Hirshfeld surface are around the position of the SO_2 groups and they are related to the other molecule drawn, but also with other ones in the vicinity.

Small differences are found in the 2D fingerprints (See Figure S17), mainly in the percentage of interactions, in such a way that the C···H interactions are 26.5% for one molecule but only 22.3% for the other one, depending on the packing of each one.

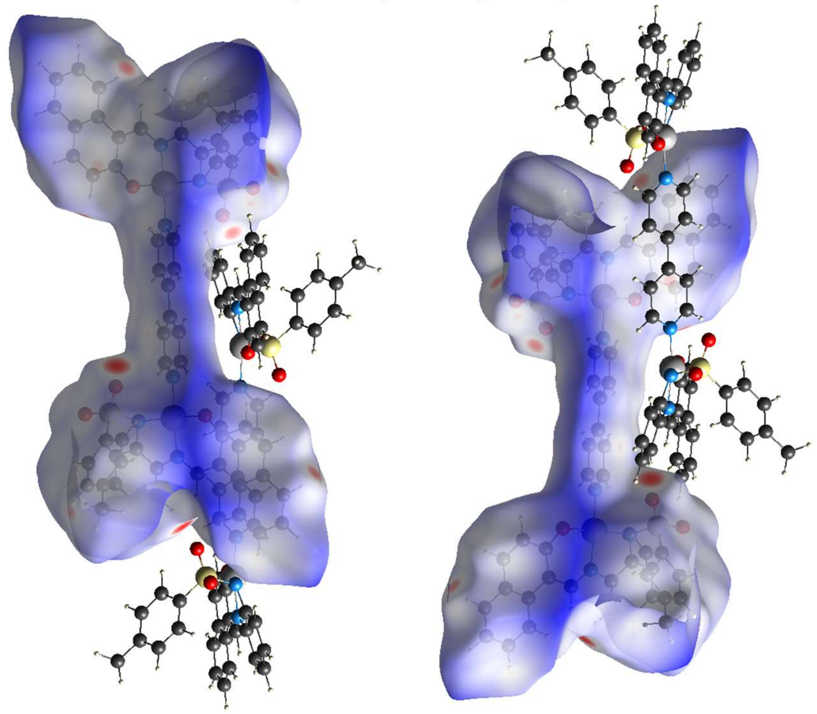

Figure S15. Front and back Hirshfeld surface modeled on d_{norm} for one of the molecules of [NiL(4,4'-bpy)]

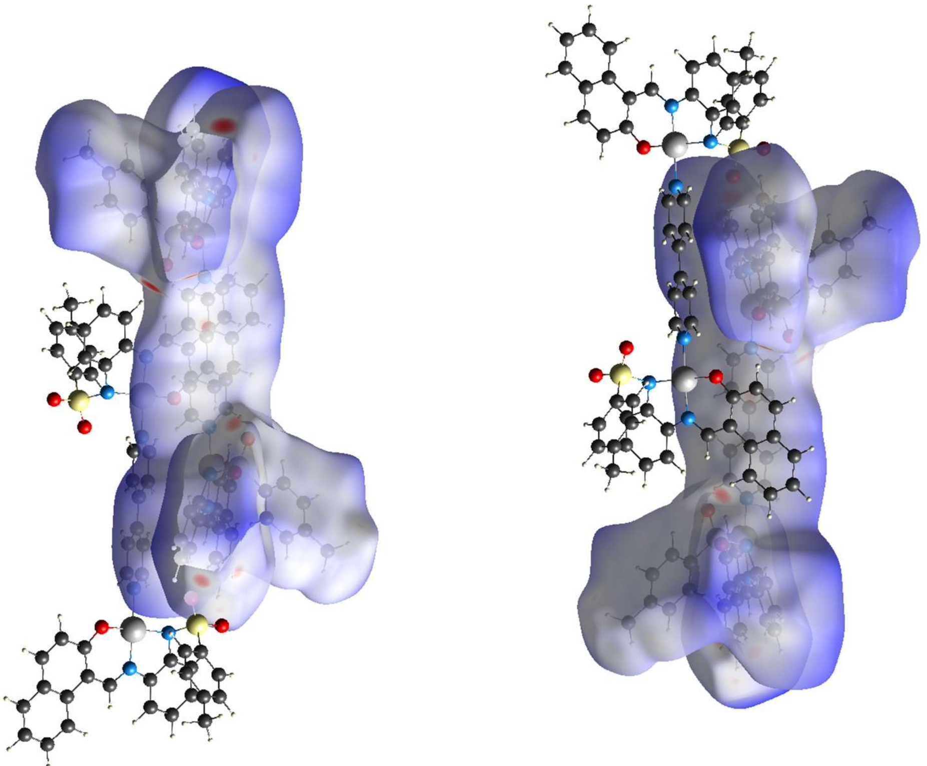

Figure S16. Front and back Hirshfeld surface modeled on d_{norm} for the other molecule of [NiL(4,4'-bpy)]

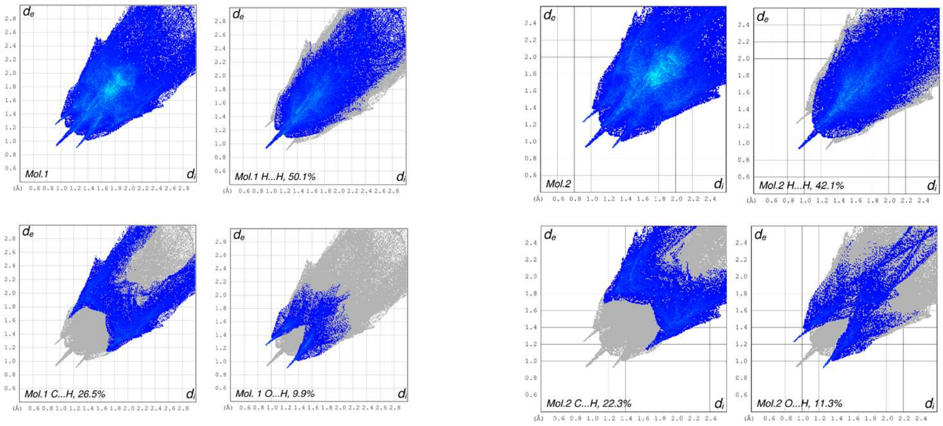

Figure S17. The 2D fingerprint plots for the two molecules in [NiL(4,4'-bpy)].

1.5.1 Supramolecular structure of [$Zn_2L_2(MeOH)_2$] **(5)**.

The supramolecular structure in [ZnL(MeOH)]$_2$ is built up through one CH···O interaction by using one of the oxygen atoms of the sulphonyl group and with one π,π-stacking interaction in which the not substituted benzene ring of the naphthol moiety is implicated. Parameters of both interactions are summarized in Table S5 and Figure S18 shows the growing parallel to the c axis of a chain of dimers. It is worth noting that the oxygen atoms of the sulphonyl group is also implied in an intramolecular CH···O interaction. These chains of dimers are connected with their neighbour chain through another C-H···O interaction between a tolyl ring and the other oxygen atom of the sulphonyl group, thus forming a 2D network in the BC plane showed in Figure S19.

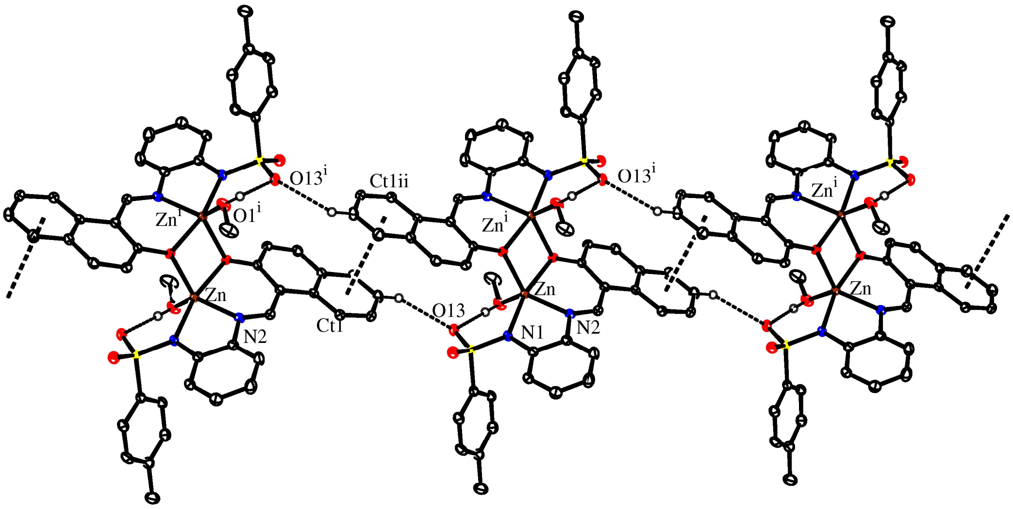

Figure S18. π,π-stacking interaction for $[ZnL(MeOH)]_2$

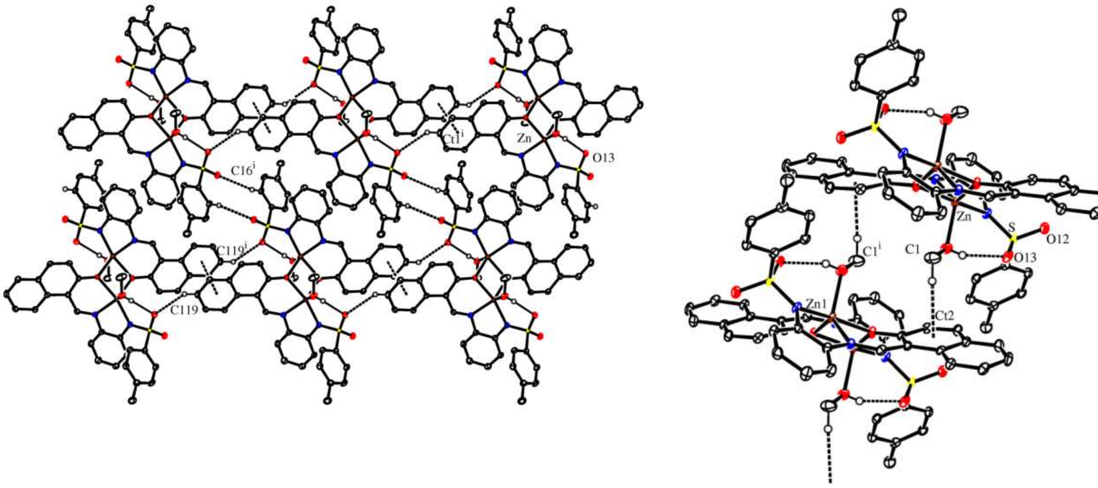

Figure S19. Left, π,π-stacking and CH...O interaction for $[ZnL(MeOH)]_2$ in the plane. Right, C-H··· π interactions.

Finally, in the a axis, other C-H··· π interaction produces the 3D network, one between the carbon atom of the coordinated methanol and the π cloud of the naphthol ring, growing along the a axis.

Table S5. Hydrogen bond and π-π-stacking interaction parameters for $[ZnL(MeOH)]_2$.

Donor-H···Aceptor	D-H	H···A	D···A	D-H···A (°)
O(1)-H(1)···O(13)	0.73(4)	1.98(4)	2.679(3)	159(4)
C(119)-H(119)···O(13*)	0.93	2.55	3.315(4)	139.8
C(16)-H(16)...O(12^{iii})	0.93	2.71	3.499(3)	143.0
C(1)-H(1C)...Ct2^{iv}	0.96	2.64	3.518(3)	152.4

Interaction	Cg···Cga	α	β	γ
Ct1-Ct1^{ii}	3.691(3)	0.00	14.72	14.72

[a] Ct: centroid; Symmetry Op.: i = x, y, z-1; ii = 2-x, 1-y, -z; iii: 2-x, -y, 2-z; iv: x-1, y, z.

1.5.2 Hirshfeld surface analysis of [ZnL(MeOH)]$_2$ **(5)**

Contrary to that made with [NiL(CH_3CN)(H_2O)], studied separately and also as a dimer, in this case only the dimeric unit was considered as input, and, as the molecule is symmetrical, only a view of the Hirshfeld surfaces is provided in Figure S20. The most important interaction (the deepest red spots) are due to interactions with a sulfonyl group and with a naphthol oxygen atom, which interacts with different molecules in the crystal network. The 2D fingerprints shown in Figure S21 clearly indicates that the shorter interactions are van der Waals ones, about 2.2 Å, while the O···H interactions, although they represent the 12.9% of the total, are longer, showing the classical spikes at 1.0+1.4 Å.

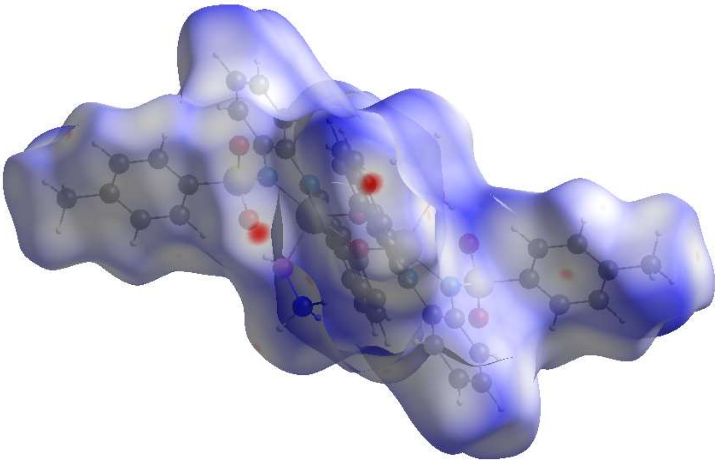

Figure S20. Hirshfeld surfaces for [ZnL(MeOH)]2

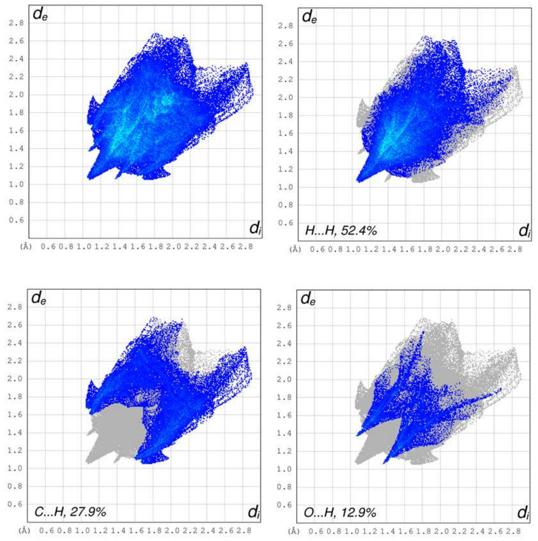

Figure S21. 2D fingerprints for [Zn_2L_2(MeOH)$_2$]

1.6.1 Supramolecular structure of [ZnL(2,2'-bpy)]·CH_3CN **(6).**

Each complex molecule is connected with their neighbour by using mainly two mechanisms. In one hand, the coordinated 2,2'-bipyridine ligand allows a π,π-stacking interaction with another coordinated 2,2'-bipyridine ligand, in a face-to-face fashion in such a way that we could consider dimeric units (Figure S22). In the other hand, a C-H···π interaction between the methyl group of the toluene moiety and the benzene ring makes a chain in the zone axis (1, 1, 0). In addition, the presence of CH_3CN solvent molecule plays an important role in the supramolecular network, in such a way that the

methyl group of this molecule binds to two complex molecules, one by using a non classical C-H···O bond and other by means of a C-H···π interaction. The combination of the mentioned interactions causes the formation of a sheet of molecules in the AB plane. The presence in these sheets of sulphonyl groups alternatively situated above and below the sheets allows another interaction to build the 3D network, by using non classical C-H···O bond (see Figure S23). Table S10 set out the geometrical parameters of these weak inter- and intramolecular interactions.

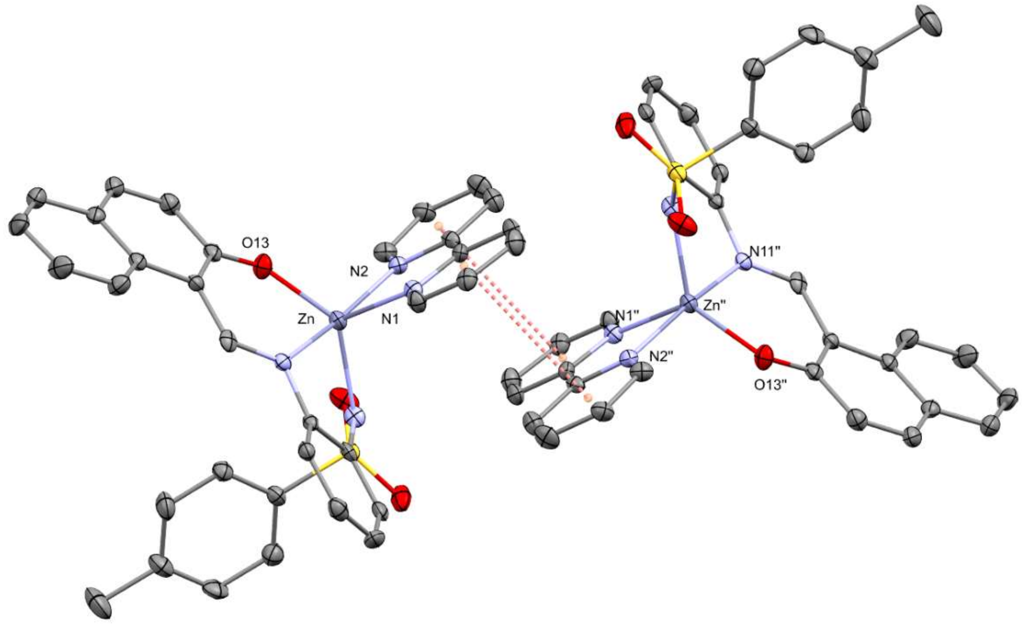

Figure S22. π,π-stacking interaction for [ZnL(2,2'bpy)]

Table S10. Supramolecular parameters for [ZnL(2,2'-bpy)] [Å and °].

D-H...A	d(D-H)	d(H...A)	d(D...A)	<(DHA)
C(1S)-H(1S1)...O(3)	0.98	2.59	3.334(5)	133.3
C(1S)-H(1S3)...Ct3^{i}	0.98	3.30	3.635(4)	102.4
C(17)-H(17A)...Ct4^{ii}	0.98	2.82	3.586(5)	135.8
C(29)-H(29)...O(1^{iv})	0.95	2.50	3.254(5)	136.8
C(112)-H(112)...O(2^{iv})	0.95	2.37	3.142(5)	137.8

Interaction	Cg…Cga	α	β	γ
Ct1-Ct2^{iii}	3.7284(2)	5.1(2)	18.6	15.4

Symmetry transformations, i: x, y+1, z; ii: 2-x,1-y,1-z; iii: 1-x, 2-y, 1-z; iv: x, 1.5 y, 0.5+z.

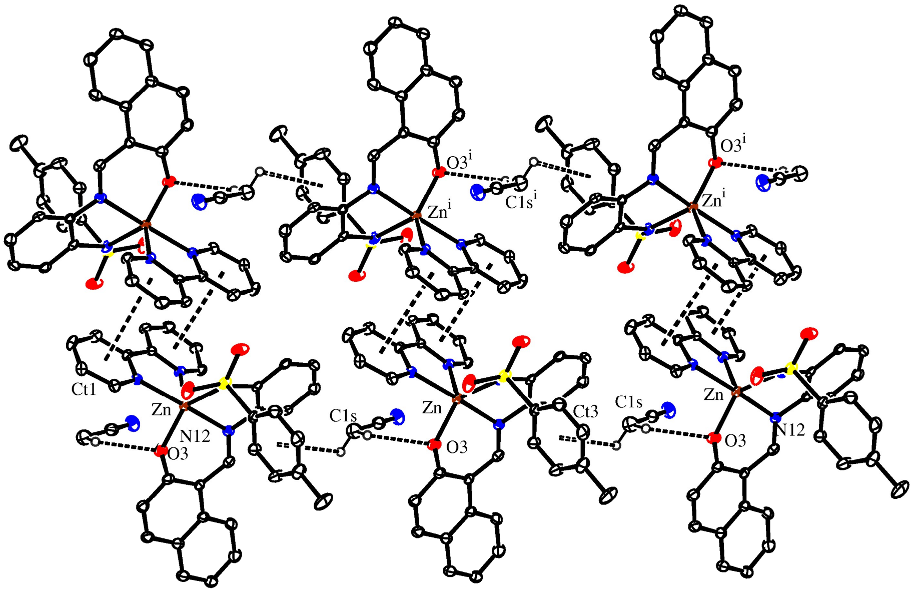

Figure S23. Some intermolecular interactions found in [ZnL(2,2'-bpy)]

1.6.2 Hirshfeld surface analysis for [ZnL(2,2′-bpy)]

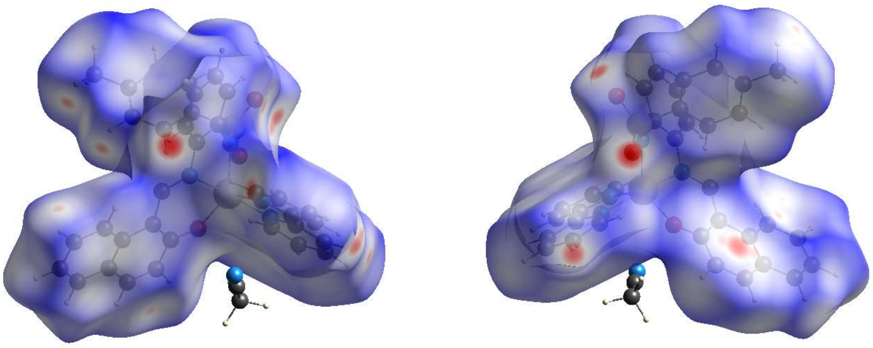

Figure S24. Front and back Hirshfeld surfaces for [ZnL(2,2'-bpy)]

In Figure S24 the front and back Hirshfeld surfaces for this compound are shown. The acetonitrile molecule was not included in the surfaces, because is a different molecule, and although in the crystal analysis the refined one was that closest to the molecule of the metal complex, it is not covalently bonded to it. In the figure on the right, the deepest red spot is close to the sulfonyl group, but other red spots are present close to hydrogen bonds. However, in the 2D fingerprints, Figure S25, the O···H interactions represent the 11.5 % of them. It should be noted that 2D fingerprints were generated only for the complex in this crystal that contains an acetonitrile molecule. For that, some interactions are formed with this solvent molecule, or, in other words, if only the complex were present in the crystal, the 2D fingerprints would be different. Once more the highest weight of the interaction is due to H…H contacts, which are also the shorter ones, about 2 Å.

The 2D fingerprints were generated not only for the complex but also for the acetonitrile molecule (right on Figure S25), as a study of the behaviour of the solvent molecule in the crystal. Of course, the interactions of acetonitrile are established with the complex, the shorter one (1.0 +1.4 Å) involves its nitrogen atom, although there is

another spike due to hydrogens at its methyl fragment interacting with an oxygen from the complex.

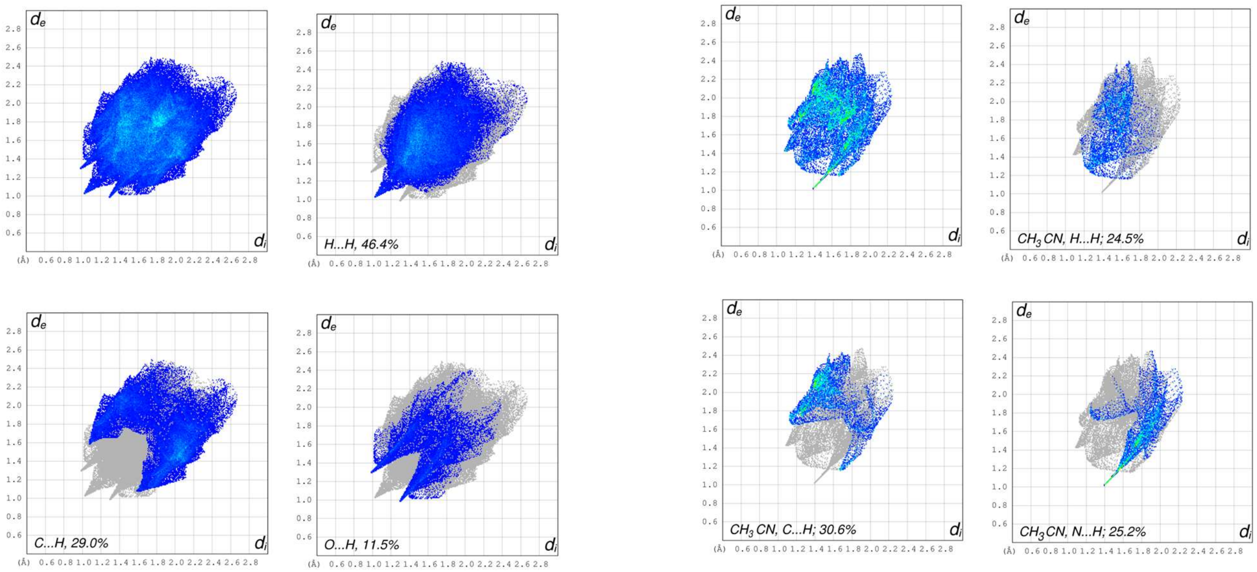

Figure S25. Left, 2D fingerprints for [ZnL(2,2'-bpy)]; right 2D fingerprints for the CH_3CN molecule in the cocrystallization with [ZnL(2,2'-bpy)]

2 Critical Points and density analysis

In an attempt to find evidence for the differences found between the bond distances based on the different metal centers—Co, Ni, and Zn—and the nitrogen atoms of the ligands in the imino and amidate groups (N (1) and N (2), respectively), the critical points associated with these bonds were calculated for the different complexes studied. The tables of critical points and densities for these interactions are shown below, depending on the complex and the specific metal center:

$[CoL_2]^-$

CP	ϱ: Density of all electrons	K(r): Hamiltonian kinetic	V(r): Potential energy density	H(r): Energy density	∇²ϱ: Laplacian
Co-N(1)	1,16E-01	2,61E-02	-1,98E-01	-2,61E-02	5,82E-01
Co-N(1)'	1,16E-01	2,62E-02	-1,99E-01	-2,62E-02	5,85E-01
Co-N(2)	9,13E-02	1,71E-02	-1,43E-01	-1,71E-02	4,37E-01
Co-N(2)'	9,01E-02	1,68E-02	-1,40E-01	-1,68E-02	4,25E-01

$[NiL(H_2O)]$

CP	ϱ: Density of all electrons	K(r): Hamiltonian kinetic	V(r): Potential energy density	H(r): Energy density	∇²ϱ: Laplacian
Ni-N(1)	1,02E-01	3,24E-02	-1,79E-01	-3,24E-02	4,57E-01
Ni-N(2)	9,58E-02	3,40E-02	-1,55E-01	-3,40E-02	3,47E-01

$[Ni_2L_2(4,4'\text{-bipy})]$ Mol. 1

CP	ϱ: Density of all electrons	K(r): Hamiltonian kinetic	V(r): Potential energy density	H(r): Energy density	∇²ϱ: Laplacian
Ni-N(1)	1,29E-01	4,08E-02	-2,38E-01	-4,08E-02	6,27E-01
Ni-N(2)	1,14E-01	3,50E-02	-2,00E-01	-3,50E-02	5,21E-01
Ni-N(1)'	1,29E-01	4,09E-02	-2,39E-01	-4,09E-02	6,28E-01
Ni-N(2)'	1,14E-01	3,50E-02	-2,00E-01	-3,50E-02	5,20E-01

$[Ni_2L_2(4,4'\text{-bipy})]$ Mol. 2

CP	ϱ: Density of all electrons	K(r): Hamiltonian kinetic	V(r): Potential energy density	H(r): Energy density	∇²ϱ: Laplacian
Ni-N(1)	1,29E-01	4,09E-02	-2,39E-01	-4,09E-02	6,28E-01
Ni-N(2)	1,14E-01	3,50E-02	-2,00E-01	-3,50E-02	5,20E-01
Ni-N(1)'	1,29E-01	4,08E-02	-2,38E-01	-4,08E-02	6,27E-01
Ni-N(2)'	1,14E-01	3,50E-02	-2,00E-01	-3,50E-02	5,21E-01

[NiL(CH3CN)(H2O)]

CP	ρ: Density of all electrons	K(r): Hamiltonian kinetic	V(r): Potential energy density	H(r): Energy density	$\nabla^2\rho$: Laplacian
Ni-N(1)	9,38E-02	2,80E-02	-1,61E-01	-2,80E-02	4,20E-01
Ni-N(2)	6,84E-02	2,02E-02	-1,06E-01	-2,02E-02	2,63E-01
Ni-N(1)′	9,38E-02	2,80E-02	-1,61E-01	-2,80E-02	4,20E-01
Ni-N(2)′	6,84E-02	2,01E-02	-1,06E-01	-2,01E-02	2,62E-01

[Zn2L2(MeOH)2]

CP	ρ: Density of all electrons	K(r): Hamiltonian kinetic	V(r): Potential energy density	H(r): Energy density	$\nabla^2\rho$: Laplacian
Zn-N(1)	8,24E-02	3,43E-02	-1,33E-01	-3,43E-02	2,59E-01
Zn-N(2)	7,72E-02	2,95E-02	-1,19E-01	-2,95E-02	2,41E-01
Zn-N(1)′	8,24E-02	3,43E-02	-1,33E-01	-3,43E-02	2,59E-01
Zn-N(2)′	7,71E-02	2,95E-02	-1,19E-01	-2,95E-02	2,41E-01

[ZnL(2,2′-bipy)] · CH3CN

CP	ρ: Density of all electrons	K(r): Hamiltonian kinetic	V(r): Potential energy density	H(r): Energy density	$\nabla^2\rho$: Laplacian
Zn-N(1)	7,50E-02	2,74E-02	-1,15E-01	-2,74E-02	2,40E-01
Zn-N(2)	8,26E-02	3,48E-02	-1,35E-01	-3,48E-02	2,60E-01

3 Cartesian coordinates of the stationary points computed

[CoL2]-

C	5,714912	0,122879	-4,351734
C	-1,623603	-1,442618	-4,875924
C	-2,819375	-0,885123	-4,42653
C	4,457524	0,063737	-3,78627
C	6,870279	0,025495	-3,556925
C	-0,468331	-1,287561	-4,118679
C	-2,875515	-0,196765	-3,218202
C	-0,510546	-0,562053	-2,927408
C	6,734053	-0,120611	-2,194329
C	4,281146	-0,108546	-2,39168
C	1,811506	-0,318583	-2,552434
C	-1,722893	-0,033886	-2,439402
C	2,984465	-0,190323	-1,755739
C	5,458898	-0,185555	-1,593047
C	1,901408	3,853674	-0,259165
C	-5,195745	2,264318	-1,341618
C	-4,19667	1,487041	-0,760484
C	-6,53056	1,947785	-1,110022
C	5,324083	-0,319321	-0,180062
C	2,89028	-0,262737	-0,341335
C	2,427597	4,771218	0,595387
C	1,189008	2,702718	0,217523
C	-4,525389	0,401423	0,0492
C	-6,886738	0,86181	-0,304368
C	-5,863376	0,098046	0,269613
C	-8,328458	0,503734	-0,083291
C	4,103233	-0,350308	0,419179
C	2,306607	4,630562	2,009533
C	0,990646	2,565023	1,617754
C	-1,518809	-1,464375	1,22006
C	2,890319	5,573643	2,881717
C	1,592046	3,51315	2,530316
C	0,100474	1,572745	2,114088
C	0,48772	-3,827712	1,191404
C	-1,405906	-0,243817	1,922439
C	-2,461965	-2,402286	1,663751
C	1,435867	-3,133217	1,943045
C	0,100812	-5,114979	1,546459
C	2,791458	5,434157	4,248503
C	1,529133	3,388131	3,939456
C	-2,245892	0,045354	2,997193
C	-3,270287	-2,118852	2,762723
C	1,99094	-3,743088	3,060224
C	2,106805	4,324368	4,772956
C	-3,174087	-0,897956	3,427161
C	0,66949	-5,712444	2,668529

C	1,618472	-5,039252	3,442013
C	2,212149	-5,674386	4,666804
H	5,808727	0,257018	-5,425749
H	-1,593361	-2,010017	-5,800844
H	-3,728317	-1,007444	-5,008636
H	3,597868	0,176459	-4,438293
H	7,85449	0,075722	-4,012328
H	0,459422	-1,74999	-4,441163
H	1,943959	-0,490125	-3,619264
H	-3,822639	0,204534	-2,877379
H	7,611535	-0,184883	-1,555361
H	2,008392	3,955205	-1,334752
H	-4,922851	3,101592	-1,975449
H	2,96718	5,631146	0,205222
H	-8,653868	-0,258013	-0,801527
H	-7,309188	2,555023	-1,564559
H	-3,742636	-0,206458	0,491817
H	6,227212	-0,383746	0,422446
H	-6,117617	-0,750321	0,900158
H	-8,488123	0,093745	0,917765
H	-8,980049	1,372175	-0,209615
H	3,42607	6,415606	2,449822
H	4,005275	-0,43489	1,497218
H	1,71946	-2,124612	1,649661
H	-2,570837	-3,34082	1,133266
H	-0,640316	-5,636475	0,949864
H	-0,207175	1,648709	3,155803
H	3,244186	6,163318	4,913305
H	-2,194585	1,015293	3,482205
H	1,041155	2,531722	4,392786
H	2,72913	-3,205308	3,650309
H	-4,000985	-2,855578	3,084572
H	0,368267	-6,718729	2,948068
H	-3,8312	-0,670092	4,260535
H	2,037985	4,192095	5,849114
H	2,030597	-6,751722	4,686855
H	3,291947	-5,505638	4,717855
H	1,776506	-5,246774	5,577195
N	0,594107	-0,293968	-2,107986
N	-1,642277	0,586848	-1,180992
N	-0,652277	-1,585945	0,129042
N	-0,418026	0,61072	1,41526
O	-2,46533	2,812939	-2,19243
O	0,754435	1,889571	-0,686857
O	0,936797	-2,960562	-1,253343
O	-2,070221	2,598528	0,272801
O	-1,30279	-3,922012	-0,712352
O	1,788184	-0,291127	0,3362

S	-2,488183	1,970035	-0,983935
S	-0,16586	-3,079542	-0,295757
Co	0,101359	0,169043	-0,346133

[NiL(H_2O)]

C	-6,562379	0,468235	-1,457259
C	-4,951303	-1,377545	-1,725525
C	-6,920466	1,683103	-0,916938
C	-5,303429	-0,107335	-1,18233
C	-3,755654	-1,962367	-1,447769
C	5,121557	2,312715	-3,82483
C	-6,017257	2,344217	-0,067722
C	3,298103	0,88558	-2,82775
C	-4,369474	0,566518	-0,344453
C	-2,780725	-1,329124	-0,608938
C	-4,781442	1,799269	0,216717
C	4,591256	1,419833	-2,740823
C	2,783699	0,08278	-1,819888
C	-3,077158	-0,038867	-0,081561
C	5,361074	1,120652	-1,613775
C	-2,085988	0,722127	0,604544
C	3,572383	-0,193853	-0,702579
C	4,860634	0,3181	-0,592753
C	0,022674	1,124709	1,607198
C	1,35018	0,630034	1,55624
C	-0,246132	2,355288	2,205673
C	2,379473	1,421355	2,078514
C	0,788771	3,130453	2,717705
C	2,097497	2,662604	2,642838
H	-7,248821	-0,07279	-2,103875
H	-5,668558	-1,882419	-2,36836
H	-7,890907	2,119694	-1,131448
H	4,717185	3,32631	-3,721625
H	-3,491827	-2,933249	-1,855507
H	2,686741	1,1043	-3,699115
H	6,210986	2,385699	-3,785877
H	4,833176	1,949882	-4,815404
H	-6,295744	3,292367	0,38335
H	1,778587	-0,320845	-1,896544
H	1,319777	-3,367847	0,611225
H	-4,137713	2,333035	0,907311
H	-2,282894	1,787603	0,720311
H	6,367285	1,522167	-1,53059
H	0,386573	-3,79405	1,794733
H	5,457935	0,088963	0,283389
H	-1,270831	2,703698	2,29269
H	3,399192	1,055355	2,066907

H	0,569459	4,084966	3,185366
H	2,912269	3,256897	3,045903
N	-0,94636	0,270158	1,046678
N	1,481745	-0,646225	0,981529
O	-1,674839	-1,977595	-0,419404
O	0,377168	-3,446865	0,895257
O	2,664987	-2,568026	-0,030119
O	3,90129	-1,227832	1,684643
S	2,930798	-1,241889	0,582124
Ni	-0,264162	-1,52162	0,750553

[Ni_2L_2(4,4'-bipy)] Mol. 1

C	-9,314295	-5,292049	1,433849
C	-10,652996	-4,969209	1,469083
C	7,806018	-2,605236	-4,122492
C	-6,942476	-4,629789	1,297511
C	-8,327357	-4,287383	1,349482
C	8,727386	-1,565104	-4,001446
C	6,694795	-2,668458	-3,286625
C	-11,032743	-3,618072	1,409506
C	-5,974072	-3,680823	1,191489
C	-8,702769	-2,915061	1,306225
C	-10,084622	-2,617891	1,325187
C	8,551517	-0,583463	-3,032156
C	6,514195	-1,695288	-2,301478
C	-6,304284	-2,288961	1,130314
C	7,458255	-0,663564	-2,168069
C	-7,666799	-1,903709	1,229948
C	-9,950743	0,501473	-4,404774
C	7,644556	-3,765571	0,27609
C	-7,622512	0,285765	-3,460506
C	8,740089	-3,814923	1,132195
C	6,520401	-3,033183	0,650474
C	2,699983	-0,322037	-1,32111
C	1,32089	-0,302057	-1,206973
C	-8,794149	1,038837	-3,612077
C	-1,555599	-0,79014	-0,433045
C	-2,926085	-0,718658	-0,253024
C	-7,989398	-0,53118	1,350918
C	-6,537516	0,781337	-2,749258
C	8,730313	-3,14748	2,360958
C	8,029722	1,085185	-0,675503
C	6,485489	-2,358115	1,870129
C	0,733925	0,141971	-0,018757
C	-0,732965	0,181537	0,144358
C	-8,847582	2,309065	-3,030939
C	9,903827	-3,231898	3,294026

C	-6,616256	2,052093	-2,17958
C	7,58646	-2,420982	2,714977
C	2,959318	0,513269	0,810653
C	-7,767981	2,82231	-2,318386
C	1,590055	0,551533	1,007569
C	-2,732148	1,210119	0,993091
C	-1,353148	1,195808	0,879457
C	7,770873	2,122448	0,251708
C	-7,520949	1,786657	1,500154
C	10,218479	2,671443	0,526855
C	-8,630136	2,189514	2,24542
C	6,427934	2,416908	0,604434
C	8,851527	2,941953	0,764568
C	11,210361	3,489728	1,029839
C	-6,620808	2,73447	0,985465
C	-8,866706	3,546618	2,435807
C	6,161062	3,575916	1,401585
C	8,538555	4,07648	1,565368
C	10,891917	4,616883	1,805336
C	-6,862185	4,09414	1,193561
C	-7,989398	4,492925	1,906367
C	7,171078	4,364979	1,856122
C	9,569429	4,897155	2,069674
H	-8,995255	-6,330783	1,467263
H	-11,407206	-5,7473	1,532996
H	7,938564	-3,359476	-4,892413
H	-6,669694	-5,681633	1,33593
H	9,573676	-1,503286	-4,678487
H	5,963962	-3,461407	-3,386997
H	-4,922437	-3,944593	1,14193
H	-12,085923	-3,352497	1,42084
H	7,654908	-4,29692	-0,669664
H	-10,106586	-0,5632	-4,20873
H	9,250687	0,244765	-2,974069
H	-7,559459	-0,701687	-3,910443
H	-9,766793	0,608314	-5,480107
H	-10,428703	-1,591586	1,253383
H	9,617879	-4,385946	0,841318
H	3,18848	-0,683632	-2,217897
H	0,715608	-0,612018	-2,05153
H	-1,138274	-1,615676	-0,998791
H	-3,588391	-1,473978	-0,657473
H	-10,875979	1,034771	-4,173498
H	-5,628008	0,198245	-2,6434
H	-9,014881	-0,27193	1,602907
H	9,042242	0,989788	-1,059946
H	10,83006	-3,44142	2,753046
H	9,760564	-4,037001	4,024225

H	10,516763	1,798367	-0,043621
H	-9,746933	2,909287	-3,140467
H	5,597469	-1,801739	2,153393
H	-9,294346	1,459898	2,697729
H	10,033216	-2,303367	3,85667
H	7,557692	-1,899998	3,668441
H	12,250307	3,250154	0,827444
H	3,648569	0,843593	1,577617
H	-3,248456	2,006357	1,515684
H	1,202378	0,880655	1,965249
H	-7,812213	3,8147	-1,882427
H	-0,776937	1,992475	1,336557
H	-9,725477	3,863763	3,019197
H	5,122721	3,792088	1,631972
H	11,68015	5,252051	2,197236
H	-6,165483	4,821882	0,796152
H	-8,169041	5,550897	2,07273
H	6,946363	5,237364	2,465027
H	9,297443	5,757281	2,676325
N	5,407481	-1,594208	-1,425513
N	7,138515	0,248004	-1,139812
N	3,509252	0,08492	-0,333254
N	-3,508352	0,266668	0,442469
N	-7,142649	0,45696	1,219935
N	-5,491299	2,179073	0,338503
O	5,132898	-4,129768	-1,271237
O	-5,31566	-1,460232	1,015837
O	3,952807	-2,619358	0,351615
O	-4,041407	2,002741	-1,710084
O	5,402974	1,719846	0,22936
O	-5,30681	4,132383	-1,29769
S	5,136417	-2,902018	-0,463769
S	-5,24993	2,66635	-1,215461
Ni	5,384126	0,107276	-0,609508
Ni	-5,383214	0,330612	0,705449

[Ni2L2(4,4′-bipy)] Mol. 2

C	7,989695	-4,492983	-1,905917
C	8,866953	-3,546681	-2,435444
C	6,862441	-4,094198	-1,193178
C	8,630283	-2,189573	-2,245213
C	6,620961	-2,734521	-0,98524
C	7,521053	-1,78671	-1,500016
C	7,767801	-2,822177	2,318967
C	8,8473	-2,30883	3,031614
C	6,616141	-2,051942	2,179795
C	9,950289	-0,500956	4,405238
C	8,79382	-1,038484	3,612472
C	7,989359	0,531168	-1,351034
C	10,084459	2,617995	-1,325386
C	11,03253	3,618216	-1,409779
C	6,537355	-0,78106	2,749199
C	7,622237	-0,285389	3,460535
C	7,666675	1,903691	-1,230232
C	2,732175	-1,210265	-0,993173
C	8,702589	2,915093	-1,306572
C	10,652711	4,969323	-1,469579
C	1,353175	-1,195978	-0,879528
C	6,304131	2,288872	-1,130724
C	8,327101	4,287386	-1,350045
C	-7,1712	-4,364712	-1,856844
C	9,31399	5,292094	-1,434486
C	-9,569579	-4,896695	-2,070588
C	-6,161155	-3,575773	-1,402156
C	2,92609	0,718496	0,252978
C	-1,590036	-0,551544	-1,00768
C	0,732987	-0,181724	-0,144411
C	-8,538672	-4,076165	-1,566116
C	5,973843	3,680707	-1,19212
C	6,942196	4,629715	-1,298216
C	-10,89206	-4,616362	-1,806284
C	-2,9593	-0,513288	-0,810758
C	1,555605	0,789955	0,433009
C	-0,733903	-0,142175	0,018719
C	-6,427988	-2,416859	-0,604855
C	-8,851606	-2,94173	-0,765173
C	-11,210465	-3,489285	-1,030658
C	-7,770923	-2,122379	-0,252131
C	-7,586203	2,421608	-2,714585
C	-1,320857	0,301658	1,207011
C	-10,218551	-2,671144	-0,527506
C	-6,485276	2,358494	-1,869698
C	-9,903437	3,232917	-3,293623

C	-2,699949	0,321644	1,321151
C	-8,730002	3,148152	-2,360491
C	-8,029738	-1,085278	0,67527
C	-6,520182	3,033349	-0,649927
C	-8,739777	3,815369	-1,131605
C	-7,644288	3,765771	-0,275461
C	-7,458204	0,663158	2,168179
C	-6,514108	1,694823	2,30178
C	-8,551462	0,582927	3,032259
C	-6,694671	2,667806	3,28712
C	-8,727293	1,564384	4,00174
C	-7,805888	2,604458	4,122982
H	8,169409	-5,550962	-2,072159
H	9,725756	-3,863833	-3,018782
H	6,165787	-4,821945	-0,795698
H	9,294451	-1,459958	-2,697585
H	7,812065	-3,814658	1,883224
H	9,746602	-2,90907	3,141433
H	10,875259	-1,035302	4,175353
H	9,014861	0,271951	-1,602983
H	10,428587	1,591719	-1,253401
H	12,085726	3,3527	-1,420992
H	9,765488	-0,606105	5,480589
H	3,248497	-2,006485	-1,515778
H	10,107123	0,563335	4,207811
H	0,77697	-1,992648	-1,33663
H	11,406882	5,747446	-1,533549
H	-6,946511	-5,237028	-2,465858
H	5,627886	-0,19795	2,643062
H	7,559144	0,70216	3,910259
H	-9,297624	-5,75676	-2,67734
H	-1,202361	-0,880505	-1,965416
H	-5,122817	-3,791977	-1,632526
H	-3,648559	-0,843459	-1,577778
H	-11,680318	-5,251417	-2,198314
H	8,994894	6,330806	-1,46807
H	3,588392	1,47381	0,657447
H	4,922188	3,94442	-1,142667
H	-7,557441	1,90078	-3,668134
H	6,669353	5,681537	-1,336804
H	1,138268	1,615467	0,998779
H	-5,597296	1,802077	-2,153012
H	-10,032526	2,304758	-3,856951
H	-9,7603	4,038585	-4,023233
H	-12,250405	-3,249655	-0,828299
H	-0,715568	0,611475	2,051615
H	-10,516799	-1,798115	0,043059
H	-10,829786	3,441838	-2,752602

H	-3,18844	0,683088	2,217998
H	-9,042256	-0,989925	1,059733
H	-9,617537	4,386405	-0,840661
H	-7,654636	4,296944	0,67039
H	-9,250661	-0,245265	2,974017
H	-5,963813	3,460712	3,38764
H	-9,573579	1,502461	4,678775
H	-7,938398	3,358549	4,893055
N	5,491397	-2,17912	-0,338378
N	7,142666	-0,457008	-1,219954
N	3,508363	-0,266809	-0,442539
N	-3,509227	-0,085122	0,333223
N	-7,138501	-0,248221	1,139743
N	-5,407397	1,59387	1,4258
O	5,306884	-4,132354	1,297904
O	4,041338	-2,002767	1,710093
O	5,315552	1,460099	-1,016185
O	-5,403004	-1,719903	-0,229647
O	-3,952631	2,619306	-0,351088
O	-5,132665	4,129445	1,272056
S	5,249933	-2,66633	1,215591
S	-5,13624	2,901865	0,46433
Ni	5,383212	-0,330701	-0,705521
Ni	-5,384104	-0,10746	0,609463

[NiL(CH_3CN)(H_2O)]

C	1,443544	-6,409134	1,978712
C	1,445125	-4,989984	1,693242
C	-5,482737	-5,865561	-0,055958
C	-1,220019	3,398092	3,962276
C	5,64932	-1,333958	2,307874
C	0,130816	3,135988	3,755606
C	-7,718433	0,500111	2,232708
C	-3,145692	-4,980801	0,291531
C	6,888015	-0,99648	1,866493
C	-4,488151	-4,741242	-0,021888
C	-2,132178	3,09494	2,957857
C	4,551306	-1,569177	1,405445
C	-8,741638	0,294297	1,292869
C	-6,450779	0,874846	1,834376
C	-2,213924	-3,949332	0,298754
C	0,572322	2,609495	2,544966
C	-4,876654	-3,432426	-0,330076
C	7,168054	-0,844652	0,475212
C	-2,627821	-2,657569	-0,0148
C	-1,71171	2,527554	1,752104

C	-3,958152	-2,389246	-0,331341
C	-0,32836	2,31844	1,510285
C	8,455804	-0,462206	0,044866
C	4,810157	-1,482542	-0,000943
C	-8,457231	0,462968	-0,044315
C	-3,817031	1,835045	0,96146
C	-6,125939	1,077089	0,469522
C	6,124418	-1,076285	-0,468617
C	-7,169441	0,8455	-0,474469
C	-4,811605	1,483361	0,002042
C	3,815421	-1,834123	-0,960221
C	3,955888	2,38965	0,340545
C	8,740048	-0,293643	-1,292364
C	0,326587	-2,317664	-1,508289
C	1,709904	-2,526506	-1,750536
C	2,627173	2,65748	0,017254
C	4,873707	3,433534	0,345761
C	6,449088	-0,87412	-1,833524
C	-0,574355	-2,608838	-2,542709
C	7,716704	-0,499464	-2,23205
C	2,213561	3,949915	-0,294117
C	-6,889225	0,997474	-1,865701
C	-4,552534	1,569905	-1,404307
C	4,487024	4,741681	0,032767
C	2,130114	-3,093685	-2,956479
C	3,144538	4,981975	-0,280456
C	5,492519	5,856658	0,014988
C	-5,650455	1,334899	-2,306895
C	-0,133121	-3,135117	-3,753537
C	1,217711	-3,396907	-3,960653
C	-1,435666	4,987631	-1,702525
C	-1,421864	6,40434	-1,999436
H	1,899027	-6,58868	2,955213
H	0,417275	-6,783481	1,985175
H	2,014928	-6,93812	1,212443
H	-5,192065	-6,676457	0,616821
H	0,599325	-2,19619	3,339146
H	5,435381	-1,435226	3,367882
H	-1,56271	3,841778	4,891914
H	-7,919852	0,355819	3,290626
H	0,857106	3,357575	4,532279
H	7,69357	-0,823412	2,576876
H	-2,824795	-5,99094	0,532045
H	-0,164445	-1,135614	2,494291
H	-5,555122	-6,287174	-1,065352
H	-6,481842	-5,522345	0,225255
H	-9,734935	-0,001206	1,616331
H	-5,697347	0,993379	2,604993

H	-3,184482	3,321147	3,103235
H	-1,171199	-4,140744	0,532914
H	-5,915705	-3,225767	-0,572986
H	1,631285	2,423096	2,410894
H	-4,131171	1,841942	2,005029
H	9,223736	-0,298686	0,797399
H	-4,264333	-1,377192	-0,577046
H	-9,225062	0,299478	-0,796957
H	4,259945	1,378737	0,593377
H	5,909915	3,228507	0,601777
H	9,733316	0,001791	-1,615977
H	4,129398	-1,840973	-2,003838
H	-1,633315	-2,422685	-2,408238
H	6,293546	5,683424	0,738352
H	5,025661	6,819491	0,237953
H	1,170233	4,142502	-0,524479
H	5,695556	-0,992663	-2,604041
H	3,182412	-3,319693	-3,102207
H	-7,694704	0,824517	-2,576199
H	2,822102	5,993793	-0,511598
H	7,917986	-0,355249	-3,290005
H	-0,859601	-3,356784	-4,530009
H	0,163398	1,134743	-2,491858
H	5,958503	5,939971	-0,973897
H	-5,436369	1,436231	-3,366868
H	1,560208	-3,840427	-4,890443
H	-1,636309	6,972086	-1,091104
H	-0,600566	2,19398	-3,338246
H	-0,439082	6,691686	-2,380338
H	-2,180297	6,630117	-2,752452
N	1,44348	-3,85733	1,464434
N	-0,002083	-1,854643	-0,225419
N	-2,578012	2,152274	0,716396
N	2,576416	-2,151276	-0,71499
N	0,000646	1,855332	0,227526
N	-1,442551	3,856758	-1,465187
O	0,73105	-1,405019	2,805149
O	3,420799	-1,866549	1,930147
O	-1,482862	-0,864533	1,505844
O	1,964223	0,311373	0,889747
O	-1,966366	-0,311105	-0,886847
O	1,482031	0,865268	-1,5032
O	-3,421875	1,867039	-1,92882
O	-0,732151	1,403623	-2,803013
S	-1,463983	-1,311252	0,094273
S	1,462611	1,311736	-0,091551
Ni	1,681038	-1,839419	1,012689
Ni	-1,682307	1,839931	-1,011005

[$Zn_2L_2(MeOH)_2$]			
C	-0,132496	6,786839	-0,866655
C	0,853113	7,596178	-0,352416
C	-1,102526	4,557261	-1,243924
C	-0,089901	5,38574	-0,688055
C	1,921054	7,009379	0,349467
C	-5,261869	-0,022029	-3,69488
C	-4,894615	0,669718	-2,543794
C	-4,618914	-1,203954	-4,051935
C	-1,069666	3,201606	-1,106243
C	-7,036962	1,466159	0,377317
C	-8,181694	0,797496	0,80462
C	0,976429	4,78157	0,040515
C	1,985212	5,644508	0,535867
C	-3,890328	0,174295	-1,702015
C	-3,609582	-1,704495	-3,236715
C	-5,790956	1,007817	0,787866
C	-0,029785	2,558504	-0,375755
C	0,889544	-0,295733	-3,012245
C	0,985995	3,342548	0,221808
C	-8,096859	-0,32139	1,635968
C	-3,257085	-1,045227	-2,05828
C	-9,329343	-1,053756	2,081552
C	-5,677074	-0,105098	1,623508
C	2,001575	2,755198	1,045563
C	-6,827056	-0,758481	2,040291
C	6,827148	0,757223	-2,040697
C	9,329494	1,052	-2,082085
C	5,677043	0,104207	-1,623674
C	-2,001339	-2,754807	-1,045771
C	8,096873	0,31999	-1,636291
C	3,257351	1,045723	2,058232
C	5,790711	-1,008487	-0,78771
C	-0,985868	-3,342278	-0,221979
C	3,610027	1,705183	3,236505
C	-0,889524	0,296824	3,012308
C	8,181499	-0,798663	-0,804605
C	0,029752	-2,558288	0,375935
C	3,890433	-0,173935	1,70214
C	-1,984864	-5,644249	-0,536724
C	7,036643	-1,466957	-0,377063
C	-0,976276	-4,781333	-0,040939
C	4,619384	1,204694	4,051726
C	4,894753	-0,669299	2,543918
C	1,069483	-3,201455	1,106575
C	-1,920682	-7,009146	-0,350527
C	5,26218	0,02263	3,694839
C	0,089899	-5,385564	0,687807

C	1,102354	-4,55713	1,244056
C	-0,852906	-7,596	0,35156
C	0,132521	-6,786691	0,866192
H	-0,962744	7,211571	-1,425166
H	0,815684	8,671865	-0,493065
H	-1,911772	5,026143	-1,798286
H	-6,043631	0,385269	-4,329494
H	-7,104635	2,339123	-0,26312
H	-5,376504	1,609864	-2,30187
H	2,714673	7,636297	0,745661
H	-9,15714	1,154756	0,485891
H	-4,883803	-1,722748	-4,967739
H	-1,845974	2,575089	-1,532473
H	0,271684	0,598101	-2,925142
H	1,302686	-0,353791	-4,02272
H	-10,235403	-0,486454	1,856869
H	2,84664	5,247446	1,06053
H	-3,070932	-2,60127	-3,529572
H	0,271432	-1,176482	-2,813152
H	-9,40763	-2,023823	1,577882
H	6,742547	1,621007	-2,694797
H	9,303954	1,250719	-3,157796
H	-9,304142	-1,252025	3,157361
H	2,627844	3,447451	1,609635
H	2,540149	-0,964782	-2,089015
H	4,700213	0,44946	-1,950213
H	9,408398	2,021829	-1,578041
H	-4,700183	-0,450218	1,950013
H	-2,627557	-3,446978	-1,610008
H	-6,742295	-1,622445	2,694133
H	-2,540714	0,964847	2,089132
H	10,235408	0,484236	-1,857972
H	3,0715	2,602073	3,529239
H	-0,2721	1,178033	2,813098
H	-2,846158	-5,24716	-1,061596
H	-1,302549	0,354609	4,022846
H	9,156878	-1,156046	-0,485806
H	-0,270995	-0,596544	2,925144
H	4,884414	1,723635	4,967406
H	-2,714149	-7,636046	-0,747054
H	7,104174	-2,339761	0,263611
H	1,84567	-2,574972	1,533079
H	5,376506	-1,609558	2,30215
H	6,043958	-0,384627	4,329459
H	1,911482	-5,02607	1,798539
H	-0,815457	-8,671708	0,492046
H	0,962646	-7,211469	1,424851
N	-3,422909	0,772433	-0,525484

N	-2,253089	-1,48714	-1,182613
N	2,253322	1,487547	1,182568
N	3,422849	-0,772206	0,525762
O	-4,731574	3,011969	-0,52874
O	-3,542373	2,175345	1,499997
O	-0,055626	1,248577	-0,268198
O	1,945753	-0,176306	-2,060487
O	-1,945781	0,176568	2,060711
O	0,055563	-1,248353	0,268575
O	3,541803	-2,175699	-1,49933
O	4,731004	-3,012031	0,529558
S	-4,31078	1,851306	0,268741
S	4,310385	-1,851522	-0,268248
Zn	-1,600584	0,001811	0,048747
Zn	1,600553	-0,001581	-0,048468

[ZnL(2,2′-bipy)]·CH_3CN

C	0,181466	-4,051276	-3,177666
C	-0,284885	-5,173681	-2,503948
C	0,227632	-2,843804	-2,493084
C	-0,688815	-5,051756	-1,180298
C	-3,431396	0,759825	-2,521575
C	-4,480223	1,624068	-2,510195
C	-0,616474	-3,802622	-0,565089
C	-5,849096	3,335351	-1,377401
C	-2,518486	0,645223	-1,414979
C	-4,73803	2,465927	-1,388292
C	-1,040628	-3,563204	0,835654
C	-6,114618	4,136311	-0,288462
C	-1,439326	-4,583538	1,697773
C	-2,715303	1,5152	-0,298754
C	-3,857221	2,405647	-0,270016
C	3,788744	0,897362	-0,619488
C	3,089626	1,931563	-1,242393
C	5,135485	1,042382	-0,305906
C	3,749506	3,115053	-1,542637
C	5,784967	2,234373	-0,615699
C	5,106023	3,286919	-1,234593
C	5,800046	4,581976	-1,544804
C	-1,831246	-4,259064	2,990709
C	-5,268909	4,065241	0,831603
C	-4,178857	3,218396	0,845058
C	-1,716953	1,645311	0,716986
C	-1,403282	-1,971089	2,473847
C	-1,817433	-2,928421	3,390879
C	1,519883	0,476436	1,5794

C	0,264257	1,077177	1,868739
C	2,575962	0,683379	2,476923
C	0,093929	1,823936	3,035285
C	2,39565	1,45729	3,620627
C	1,157531	2,024397	3,909965
H	0,5058	-4,103894	-4,210568
H	-0,337235	-6,136803	-3,001219
H	-3,24158	0,113702	-3,373617
H	-5,151507	1,679641	-3,364347
H	0,603519	-1,941932	-2,96164
H	-1,061748	-5,916919	-0,646462
H	-6,497634	3,357024	-2,250045
H	-1,441028	-5,617568	1,376103
H	-6,97189	4,80242	-0,28721
H	5,534915	4,945477	-2,542015
H	2,036343	1,812483	-1,478809
H	5,661893	0,227666	0,180253
H	3,204826	3,922599	-2,024985
H	6,837591	2,347813	-0,370118
H	-2,142989	-5,040599	3,675931
H	5,507835	5,360087	-0,830331
H	6,886281	4,475859	-1,493923
H	-5,480646	4,672916	1,706999
H	-1,74282	2,565553	1,307307
H	-3,581259	3,167553	1,749066
H	-1,372063	-0,917565	2,73266
H	-2,11734	-2,632076	4,389368
H	3,536052	0,21747	2,285429
H	-0,88446	2,234908	3,268841
H	3,230634	1,597612	4,30131
H	1,013166	2,603919	4,816475
N	-0,162387	-2,724571	-1,2234
N	-1,022966	-2,27985	1,232847
N	1,565777	-0,271374	0,399774
N	-0,749165	0,807457	0,937096
O	-1,561653	-0,209316	-1,539007
O	2,625157	-1,256488	-1,599196
O	3,870722	-1,435482	0,56265
S	2,965117	-0,654098	-0,300195
Zn	-0,271544	-0,872162	-0,201338

4 IR Spectra

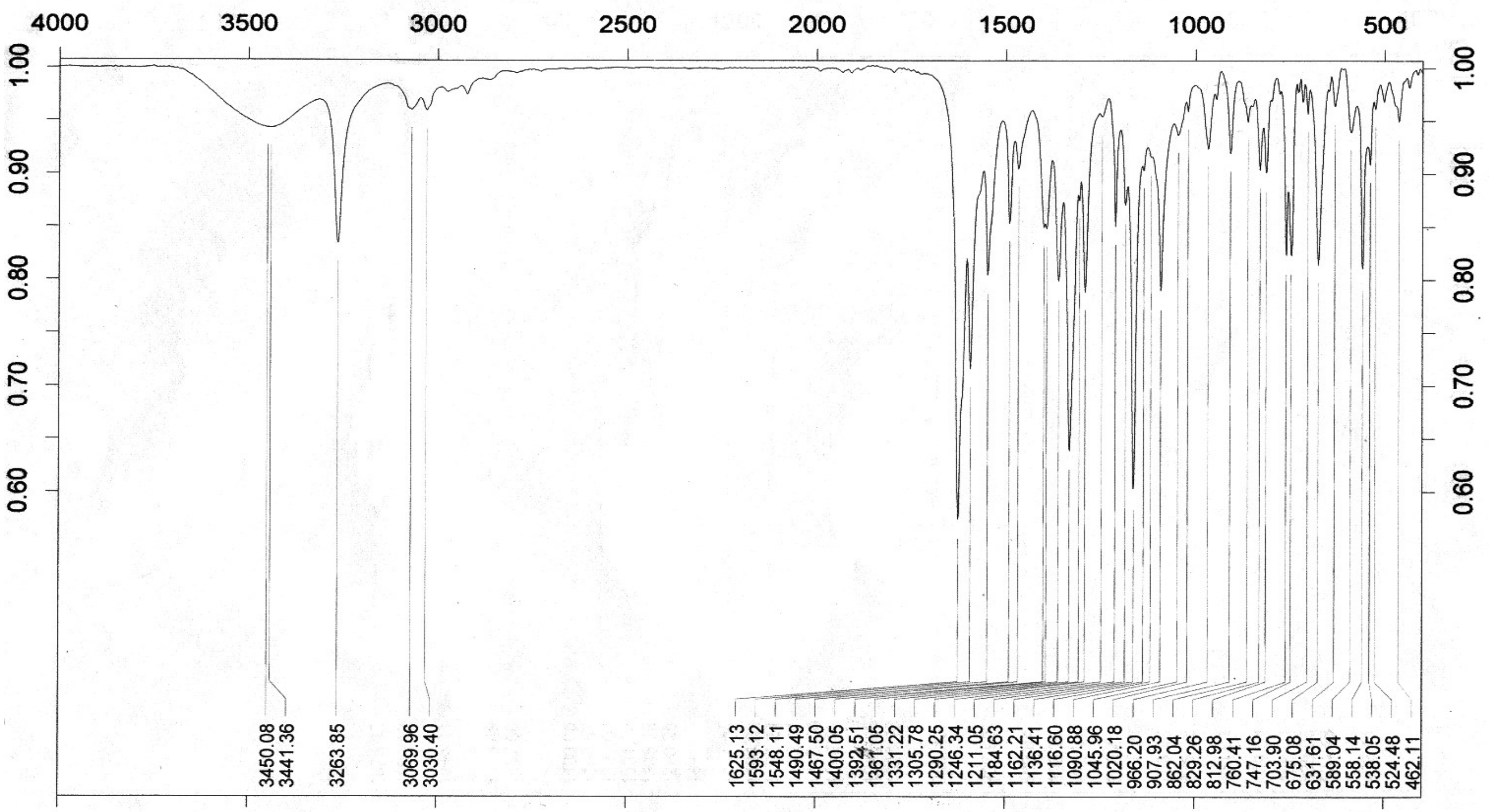

Figure S26. IR spectrum of the ligand (E)-N-(2-(((2-hydroxynaphthalen-1-yl)methylene)amino)phenyl)-4-methylbenzenesulfonamide (H_2L)

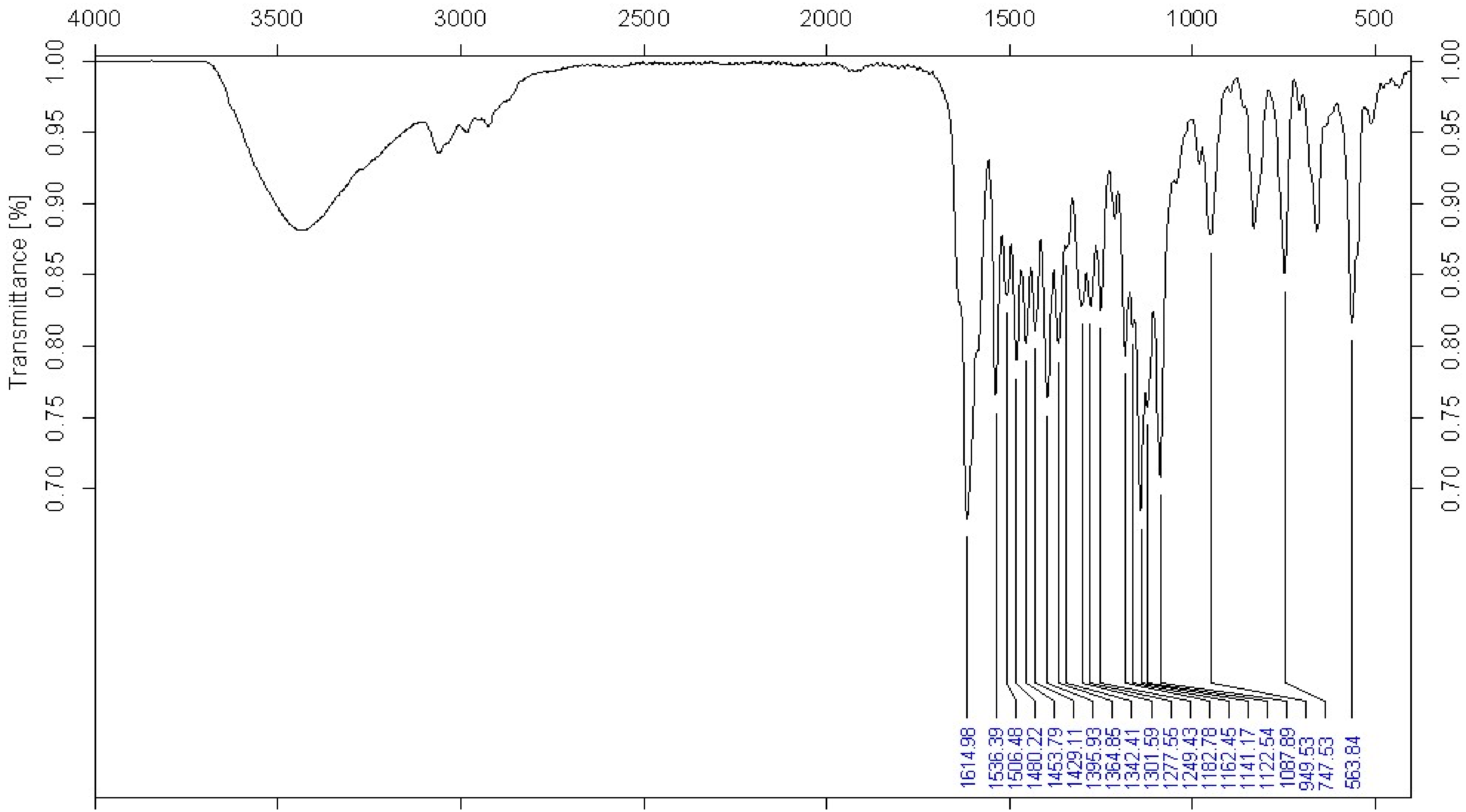

Figure S27. IR spectrum of complex $NEt_4[CoL_2]$

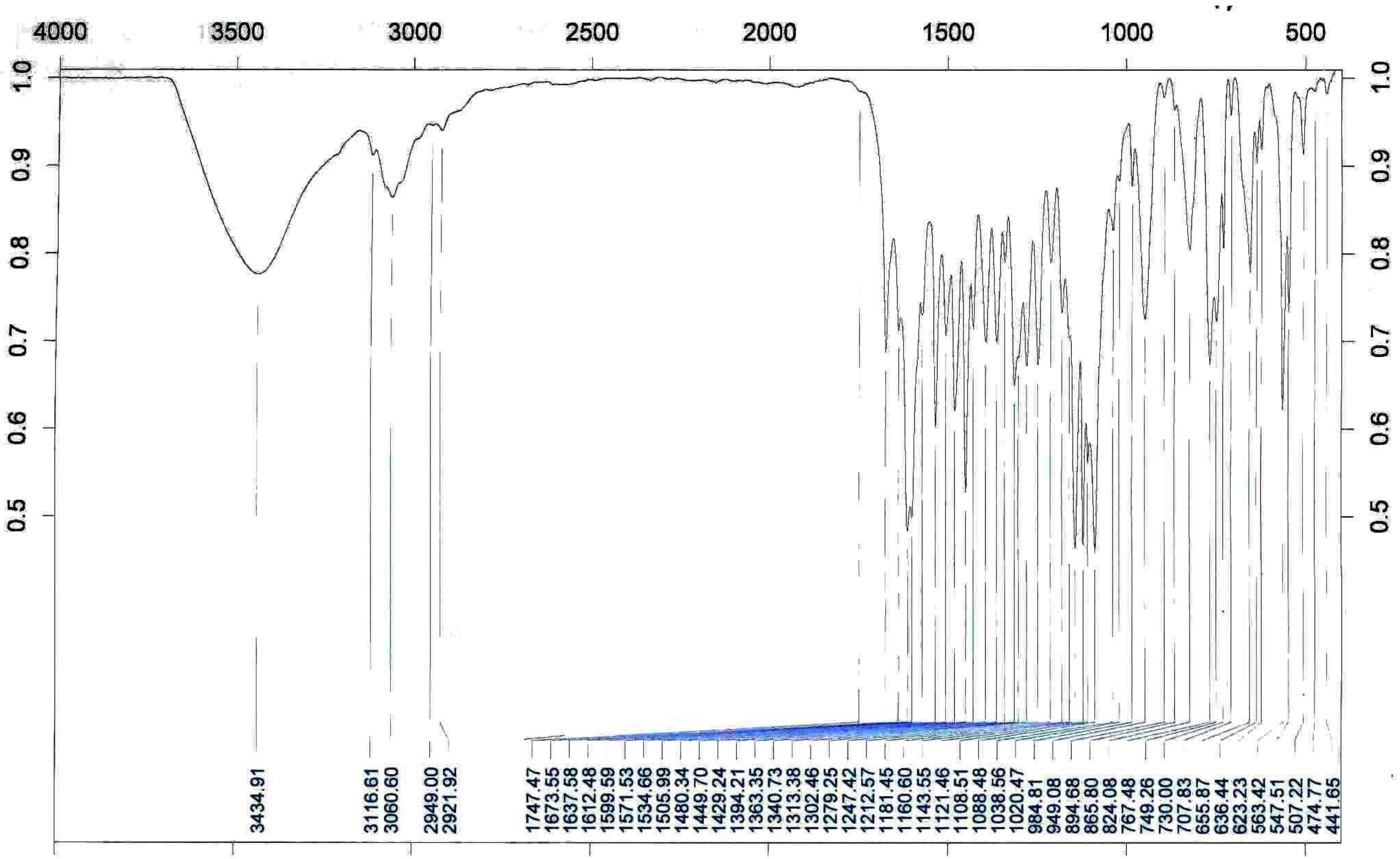

Figure S28. IR spectrum of complex [CoL(2,2′-bipy)]

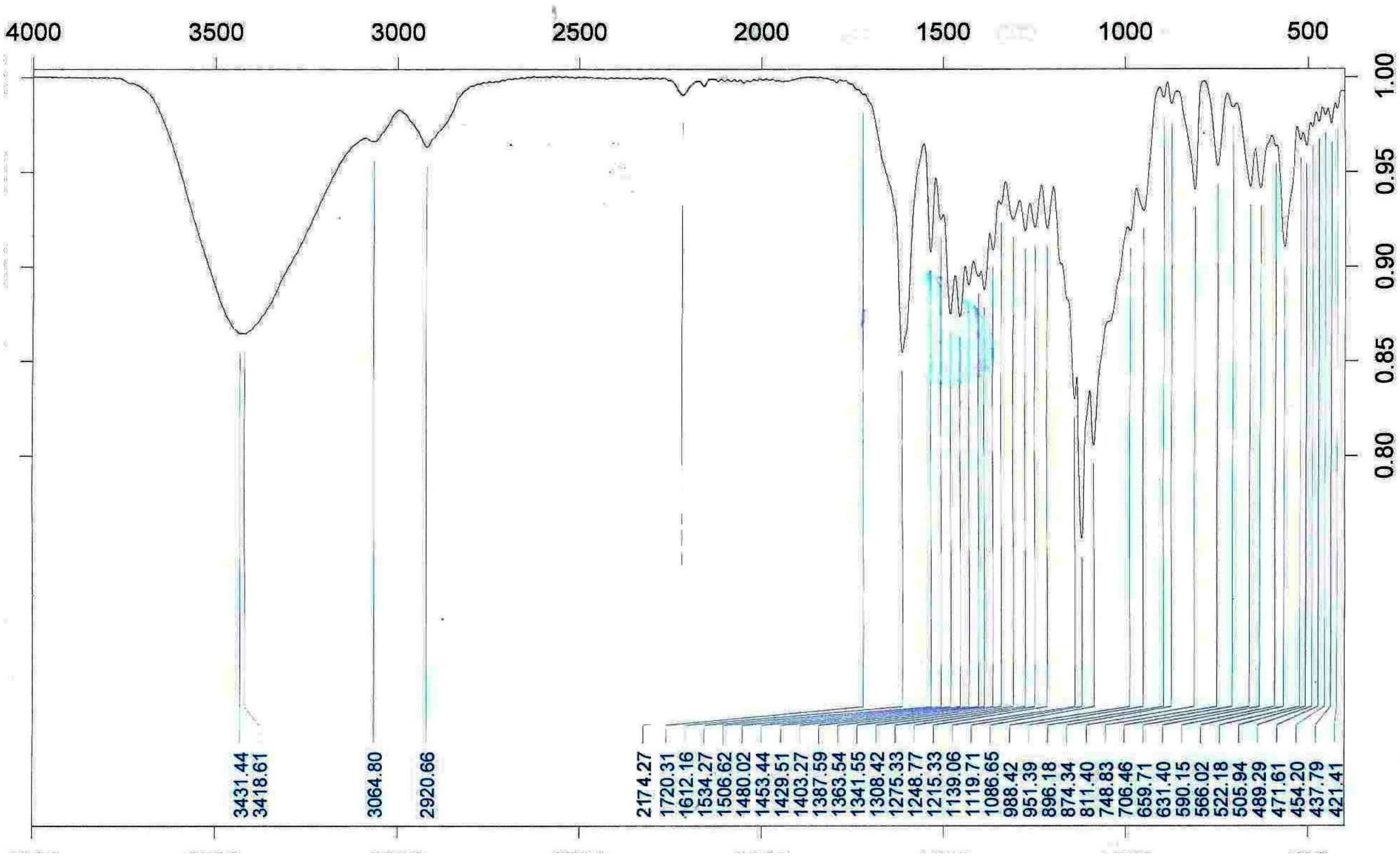

Figure S29. IR spectrum of complex [(Co_2L_2(4,4′-bipy)]

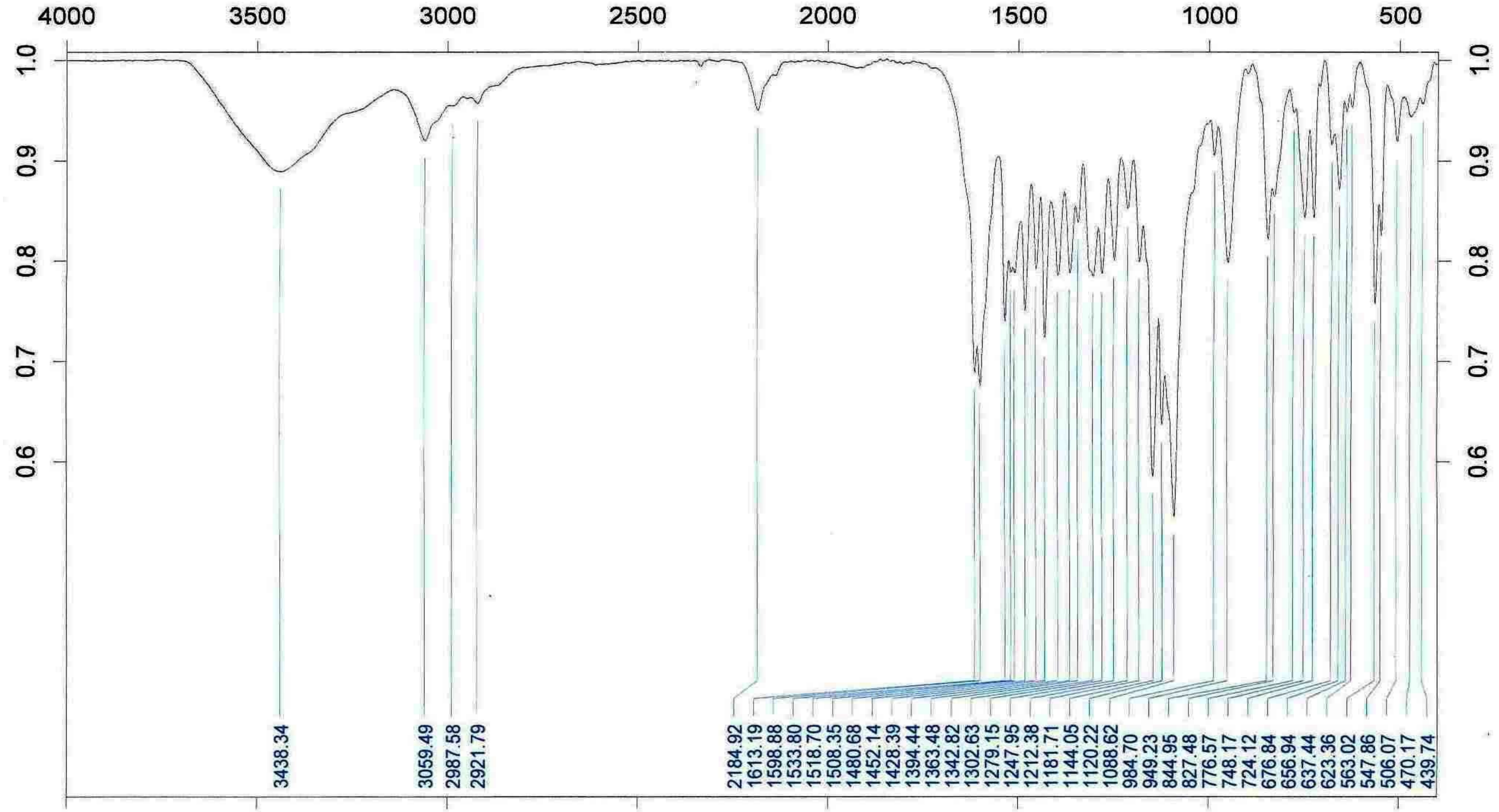

Figure S30. IR spectrum of complex [CoL(phen)]

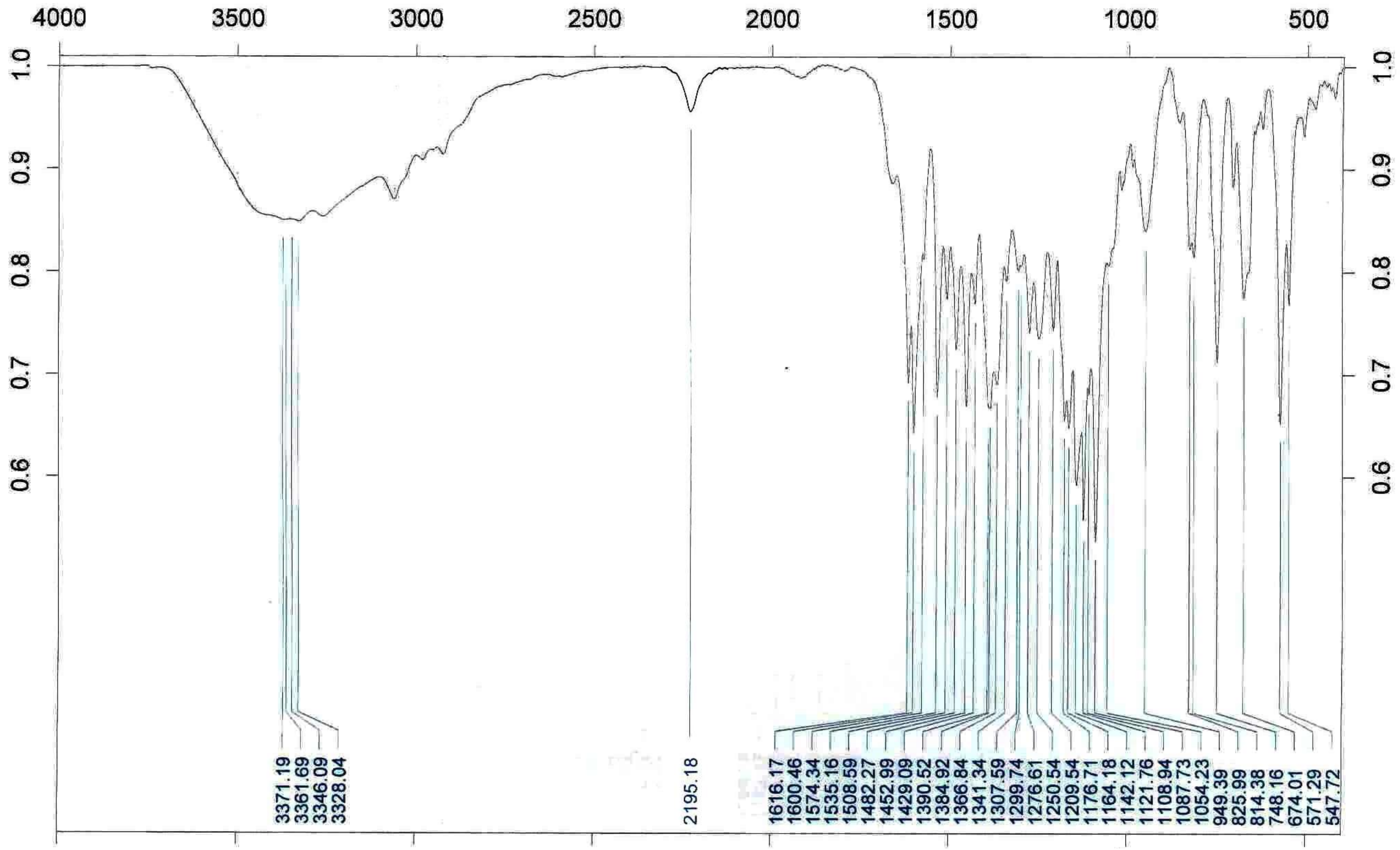

Figure S31. IR spectrum of complex [$NiL(CH_3CN)(H_2O)$]

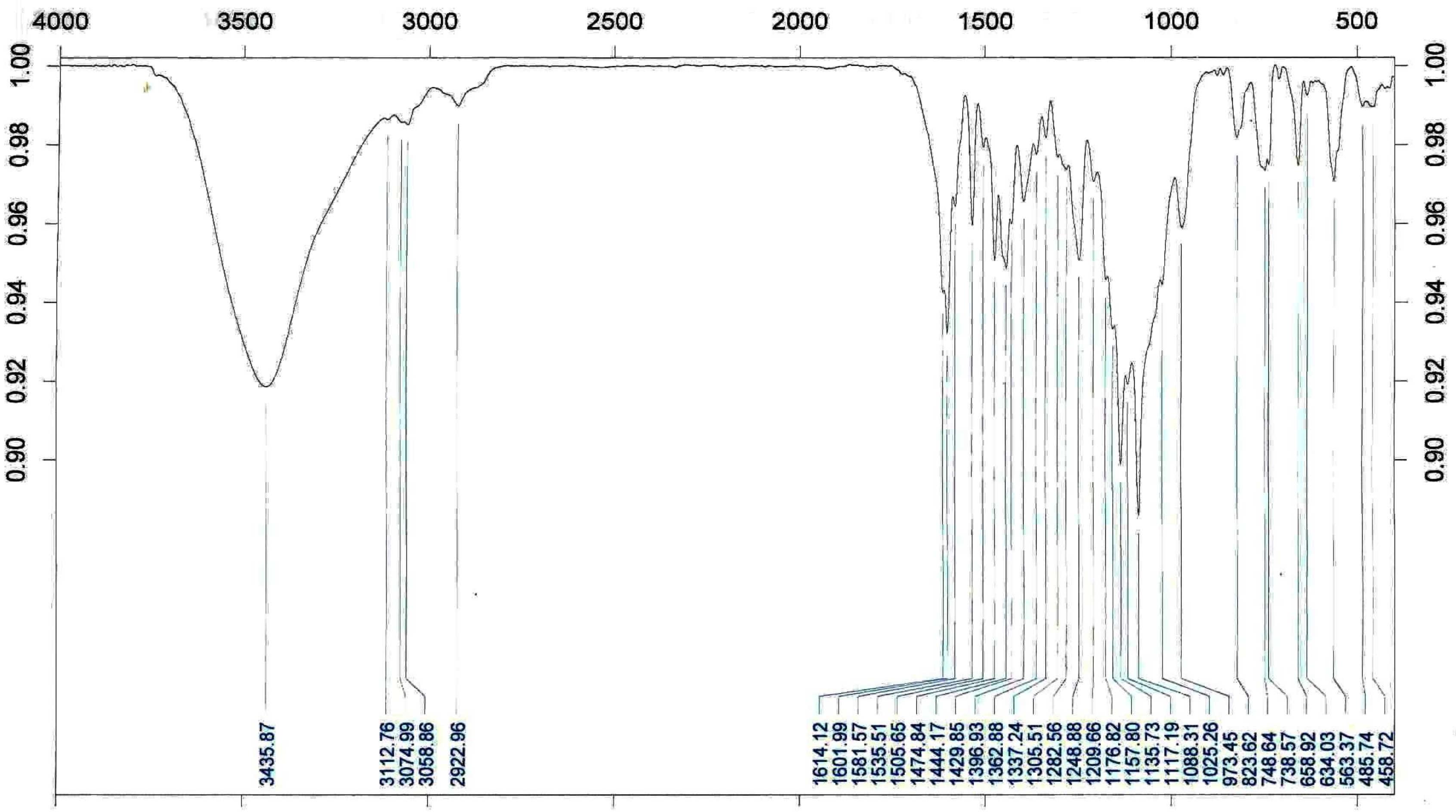

Figure S32. IR spectrum of complex [NiL(2,2′-bipy)]

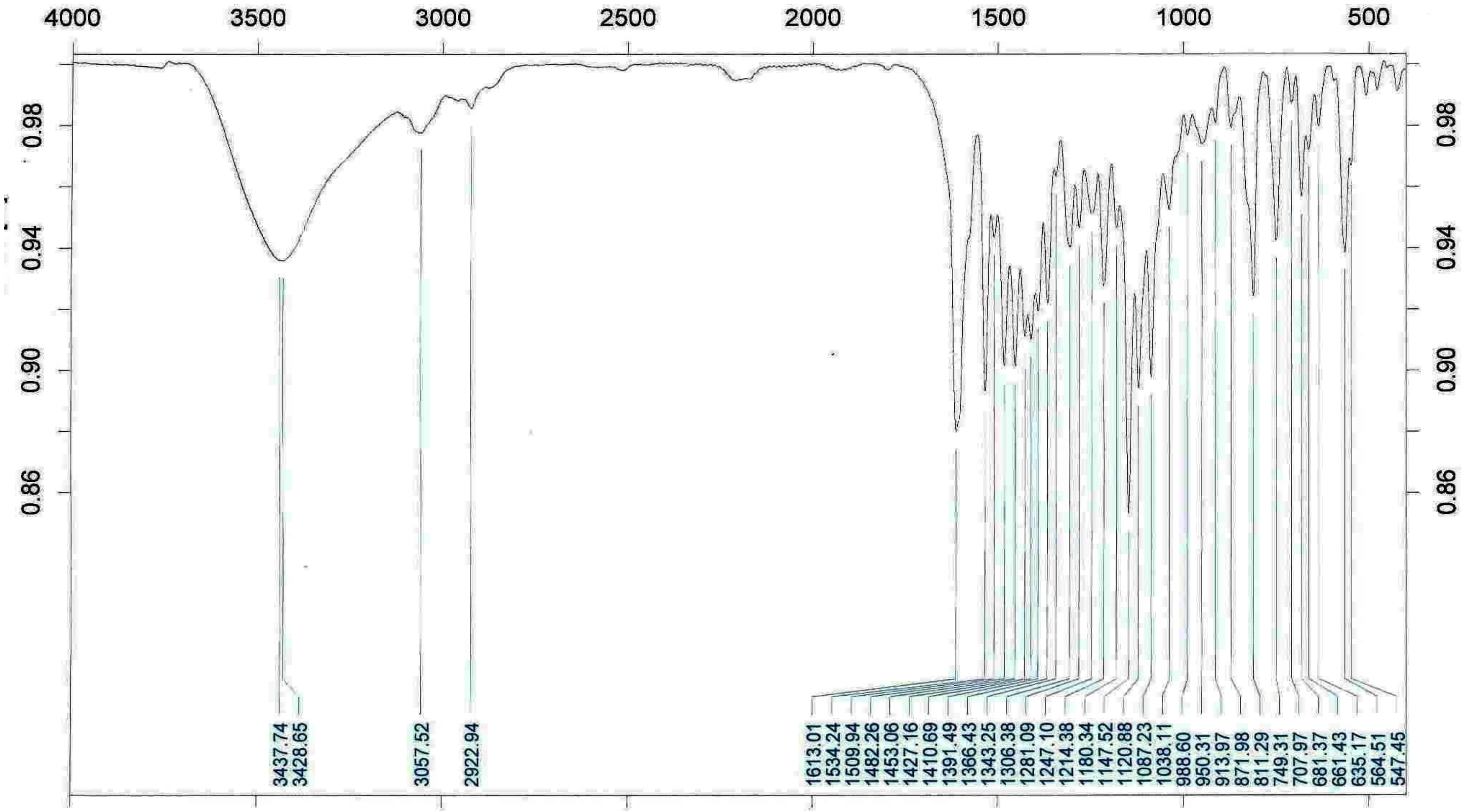

Figure S33. IR spectrum of complex $[Ni_2(L)_2(4,4'\text{-bipy})]$

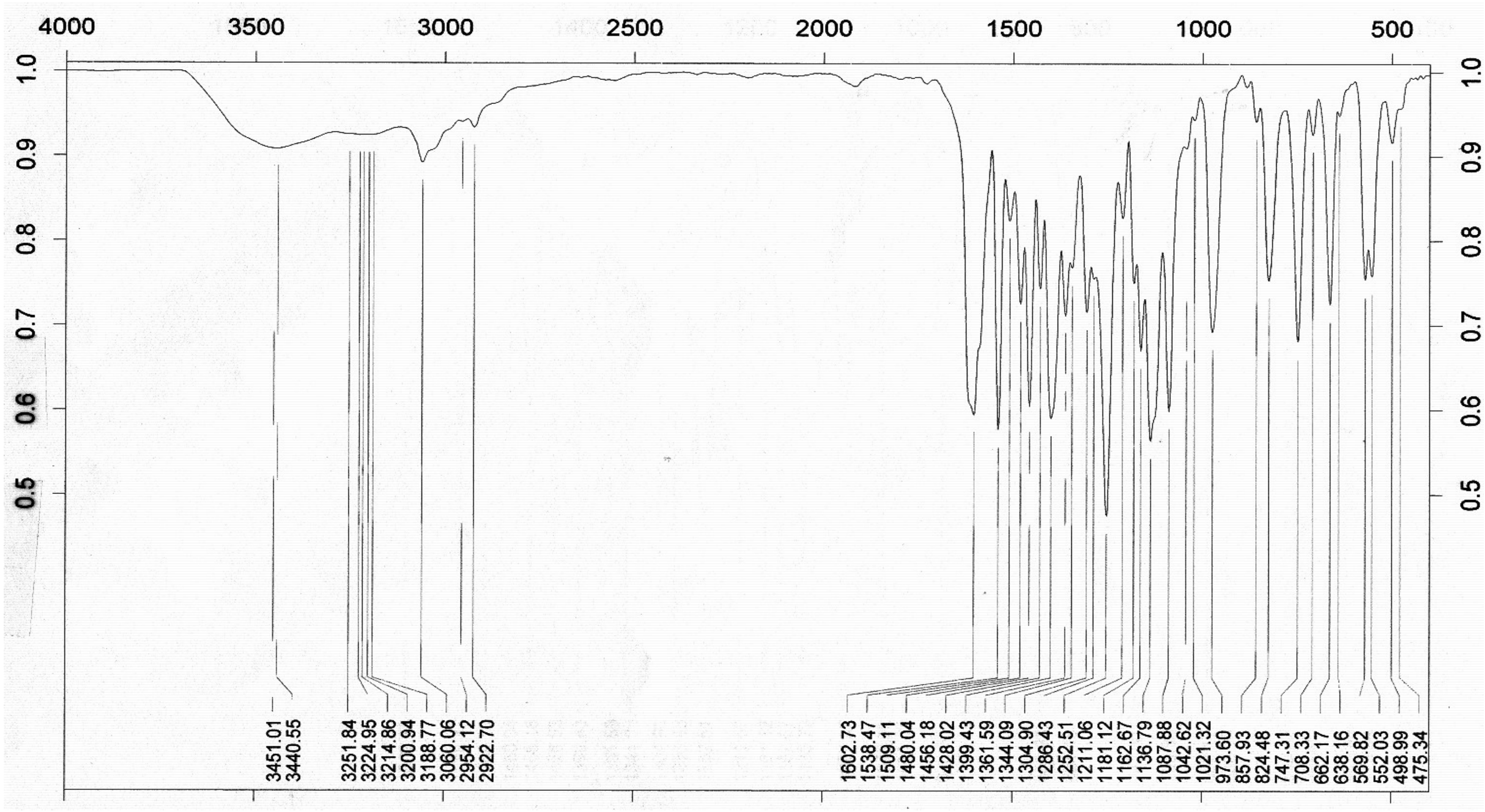

Figure S34. IR spectrum of complex [NiL(phen)]

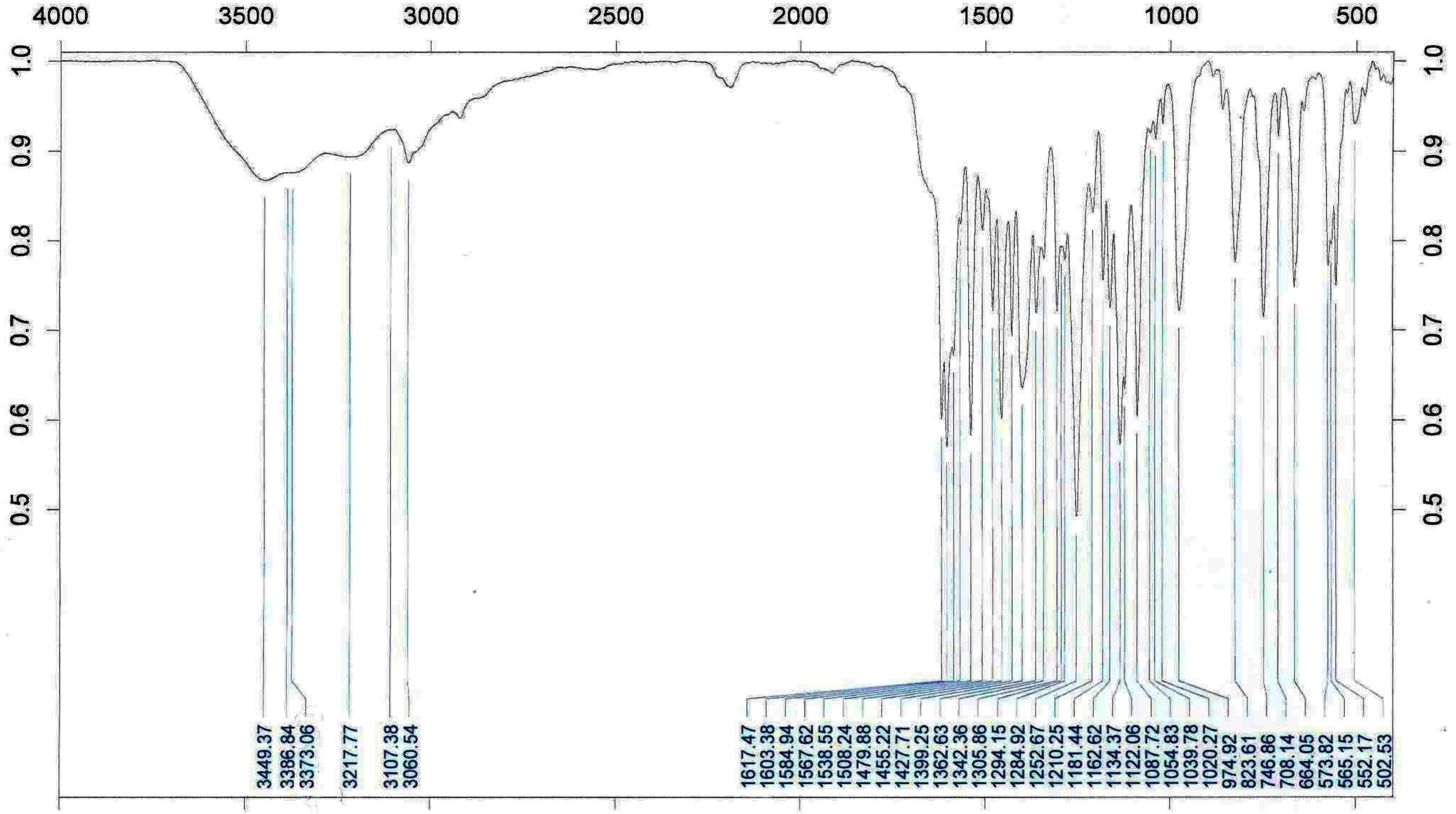

Figure S35. Espectro IR de $[Zn_2L_2(CH_3OH)_2]$

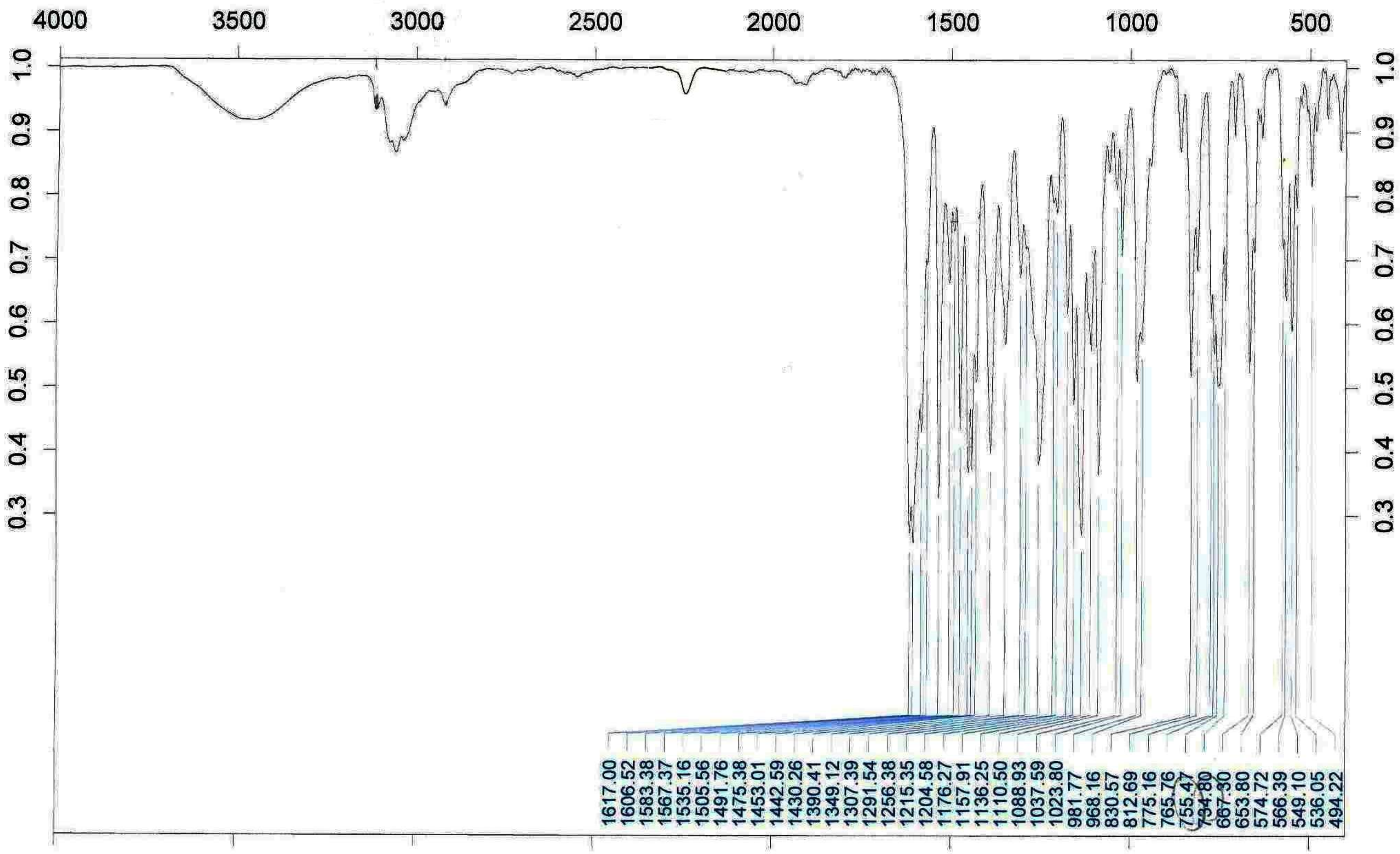

Figure S36. IR spectrum of complex [ZnL(2,2′-bipy)]

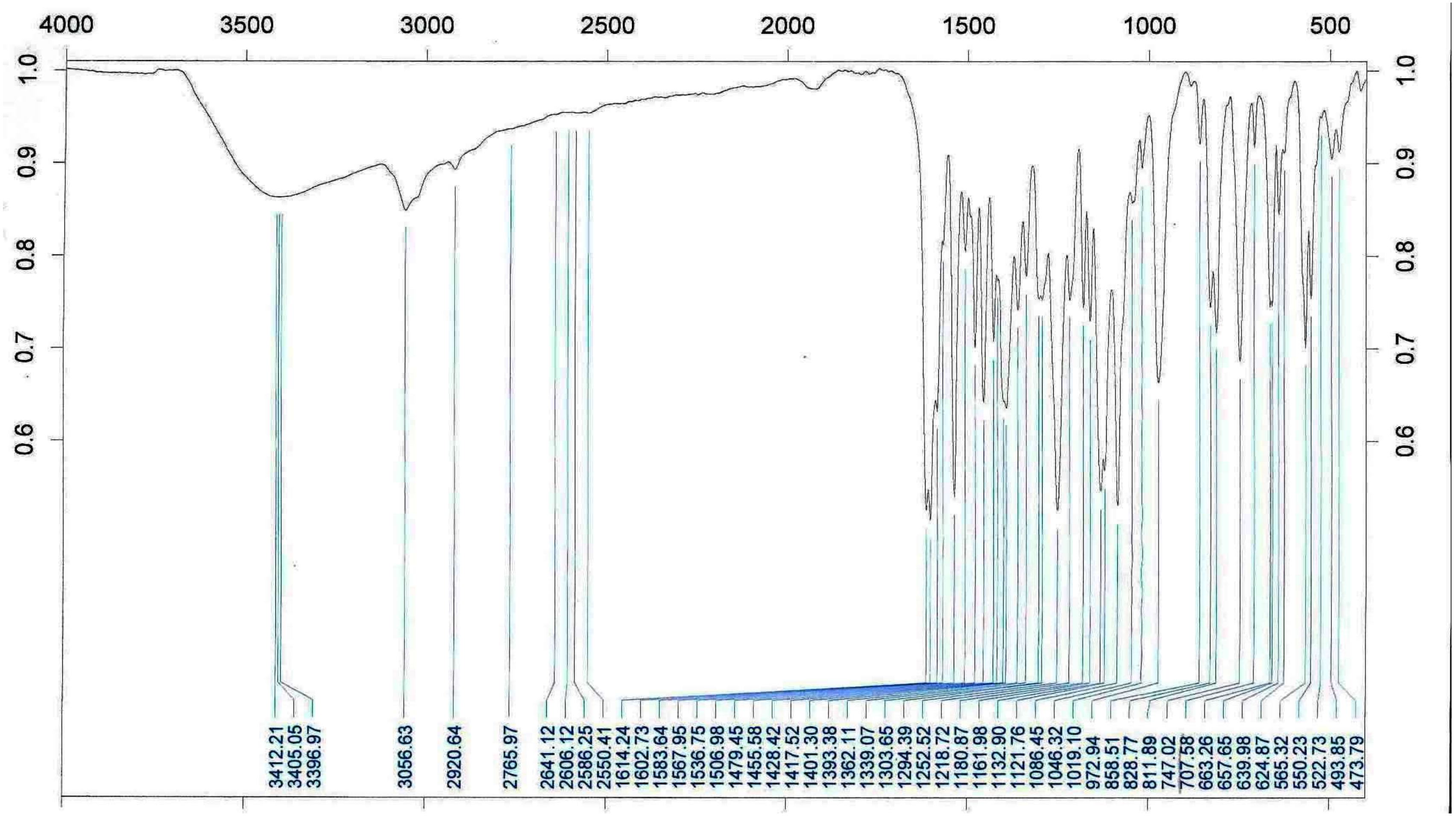

Figure S37. IR spectrum of complex [Zn_2L_2(4,4′-bipy)]

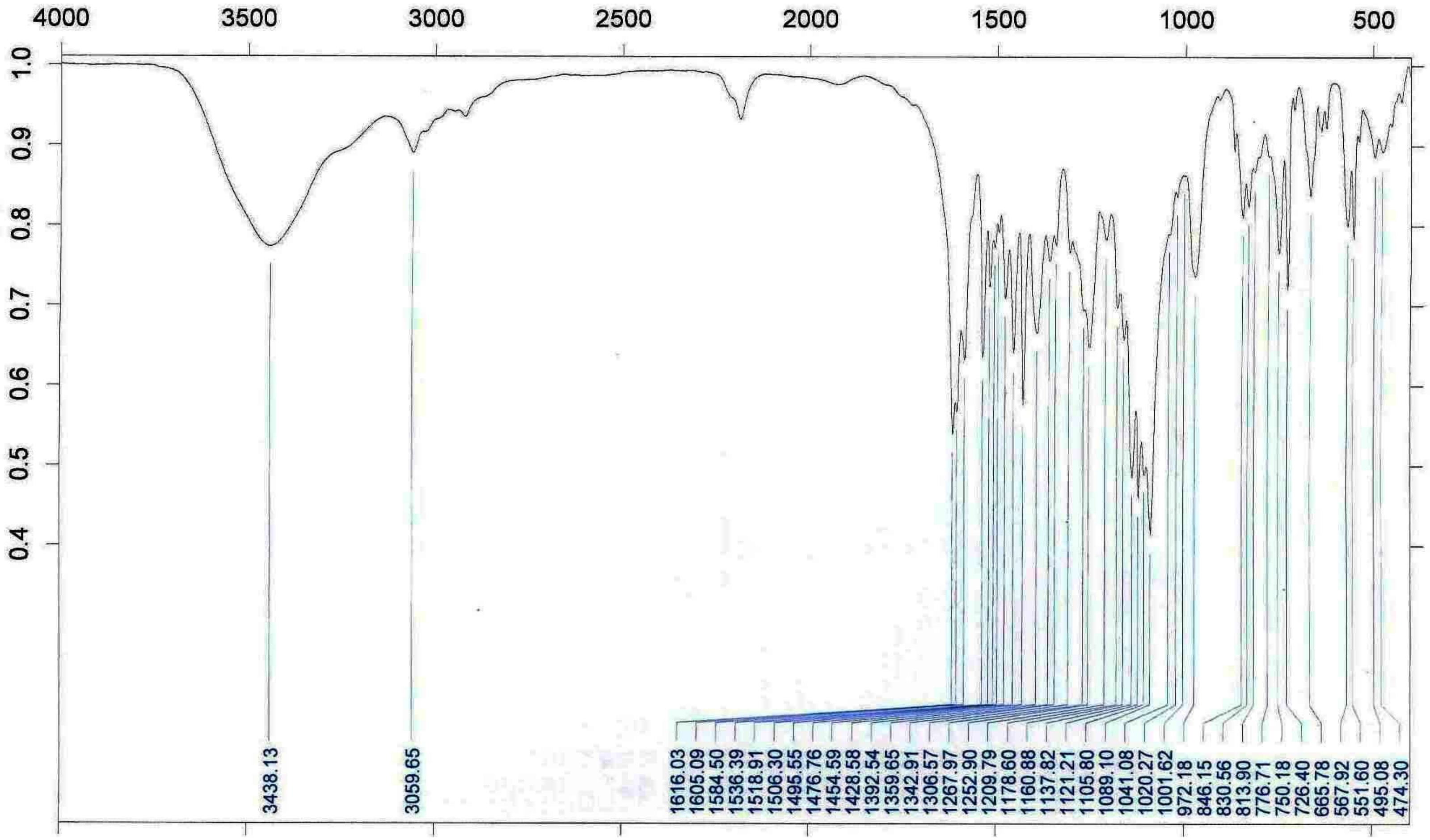

Figure S38. IR spectrum of complex [ZnL(phen)]

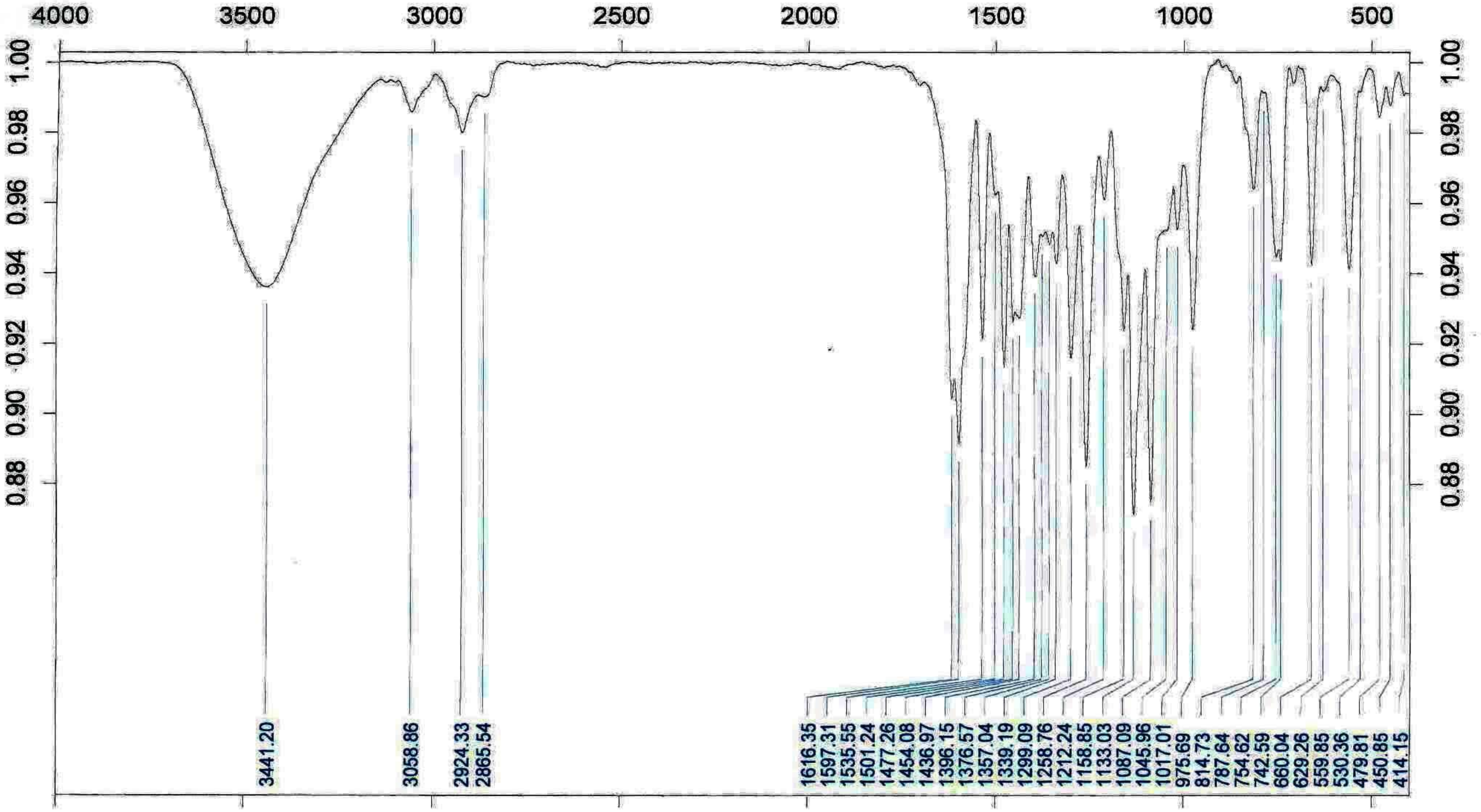

Figure S39. IR spectrum of complex [CdL(2,2′-bipy)]

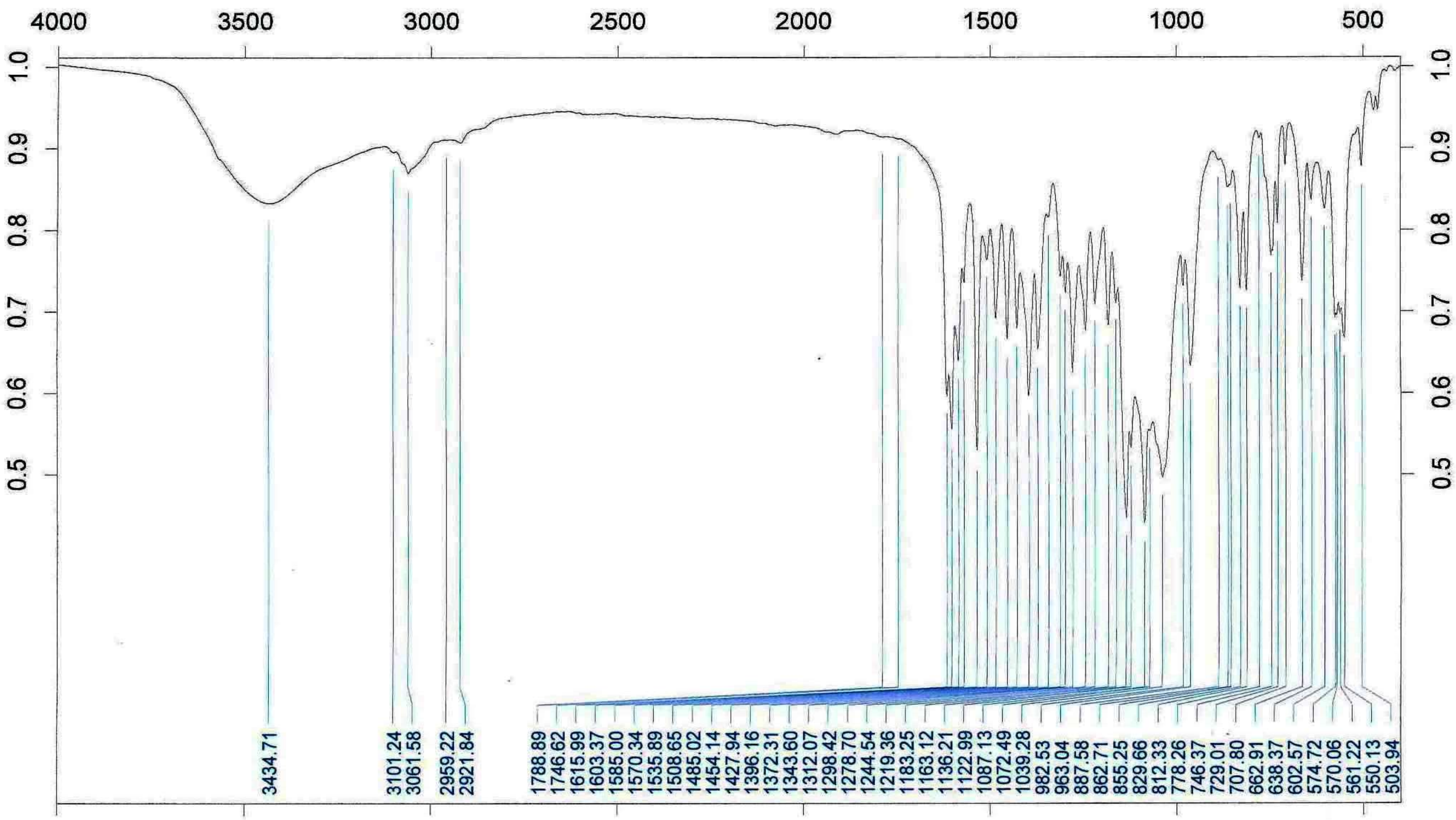

Figure S40. IR spectrum of complex [Cd_2L_2(4,4′-bipy)]

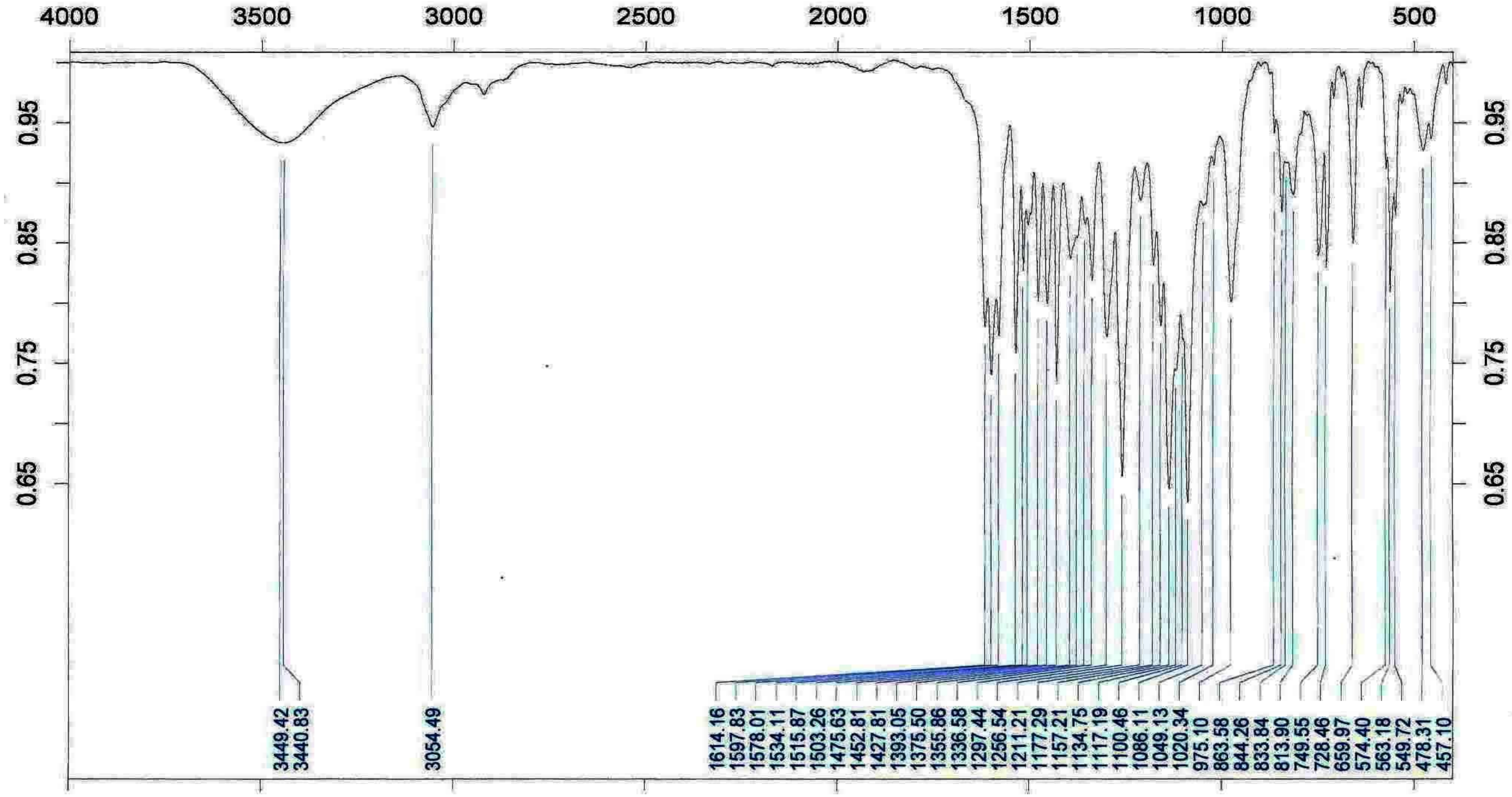

Figure S41. IR spectrum of complex [CdL(phen)]

5 ¹H NMR Spectra

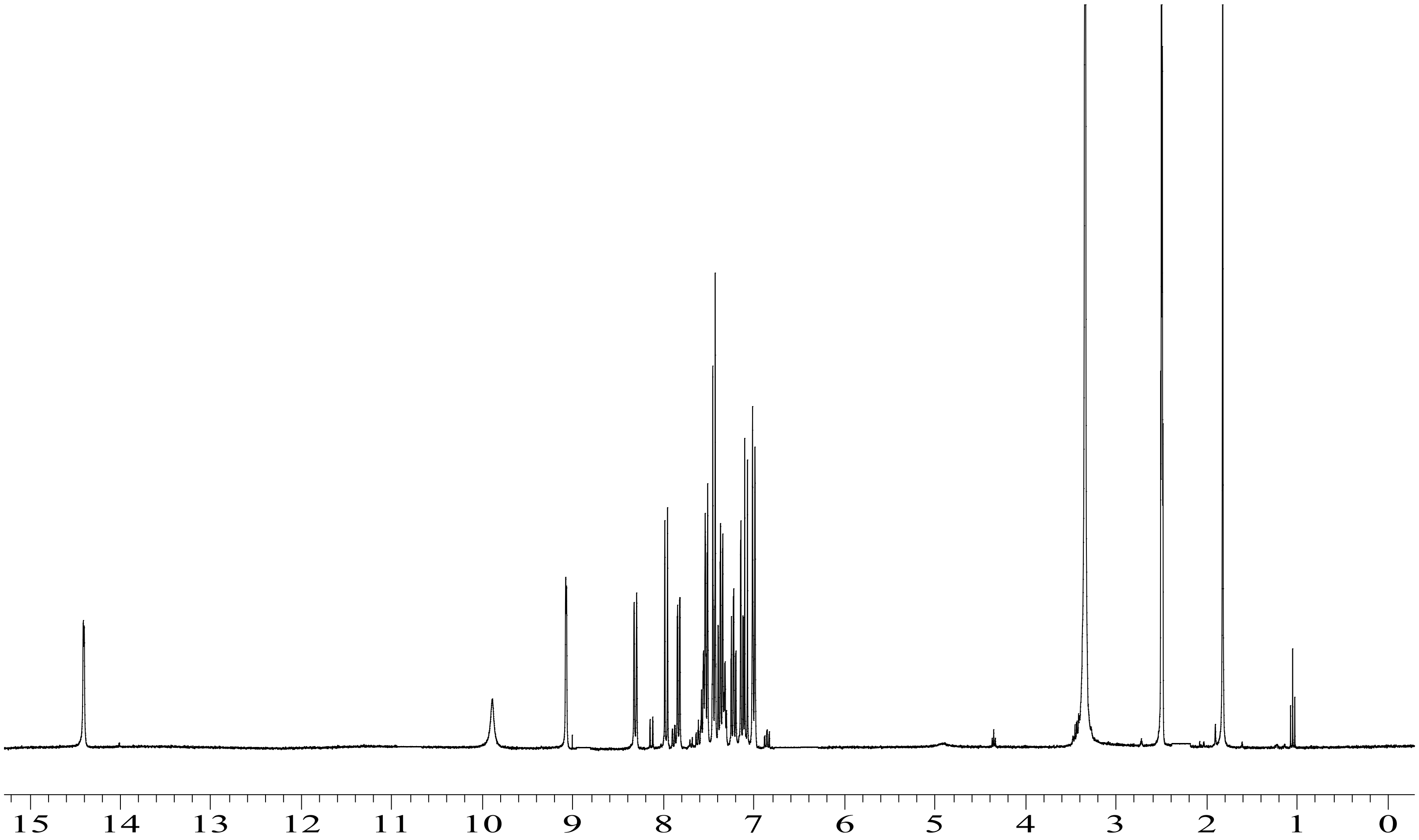

Figure **S42.** ¹H RMN spectrum of the ligand (E)-N-(2-(((2-hydroxynaphthalen-1-yl)methylene)amino)phenyl)-4-methylbenzenesulfonamide (H_2L)

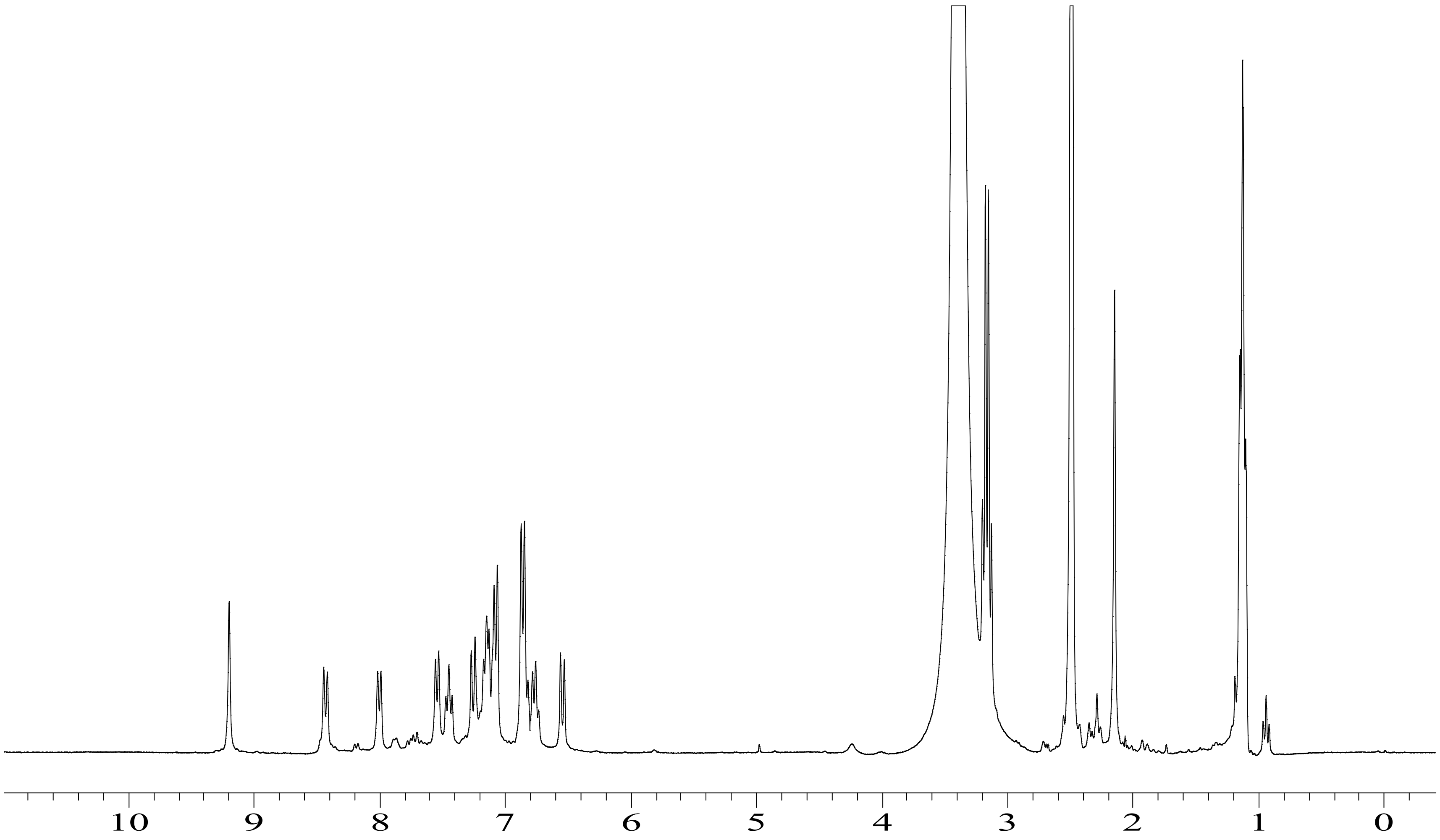

Figure **S43.** ^{1}H RMN spectrum of compound $NEt_4[CoL_2]$

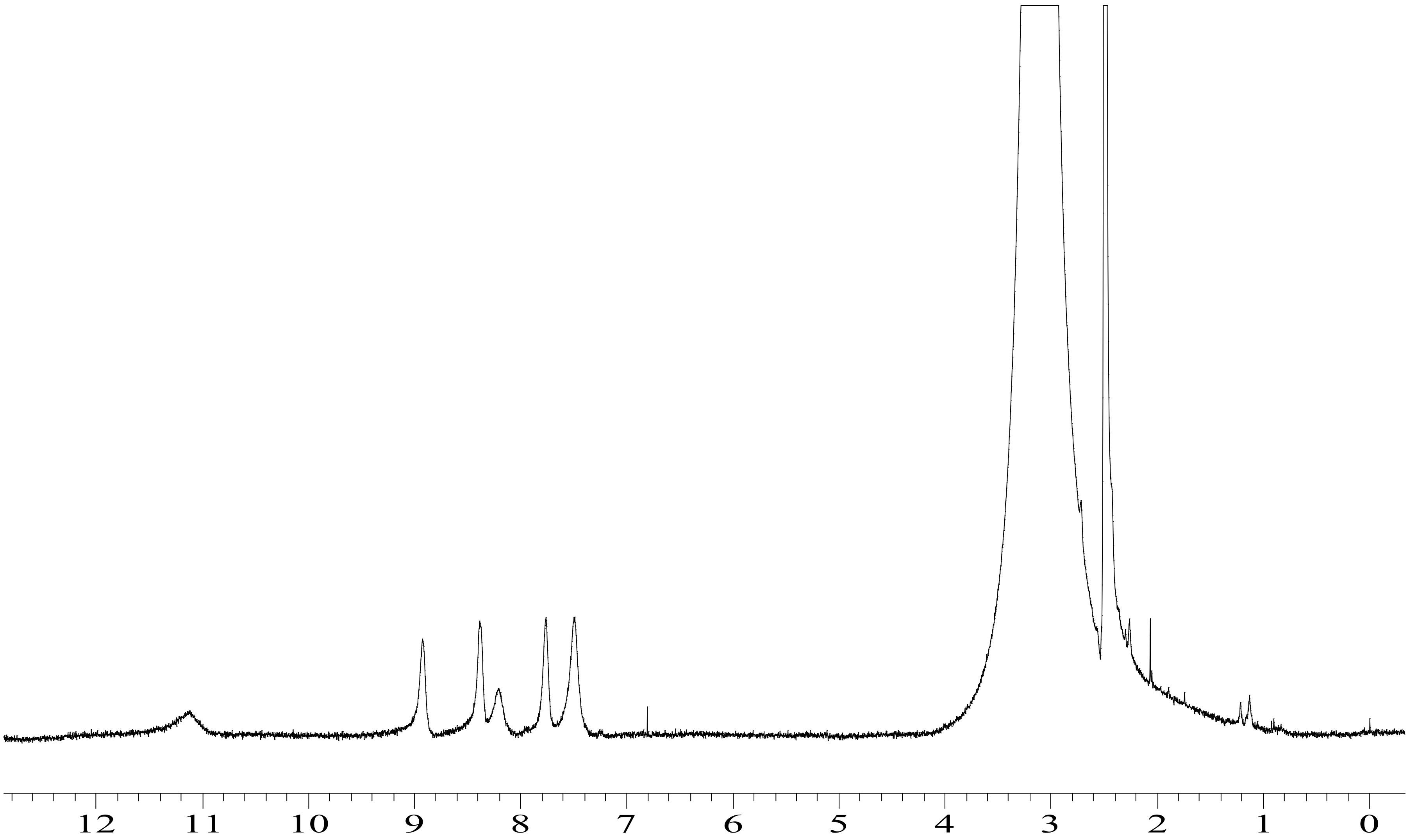

Figure **S44.** ^{1}H RMN spectrum of compound [Ni_2L_2(4,4′-bipy)]

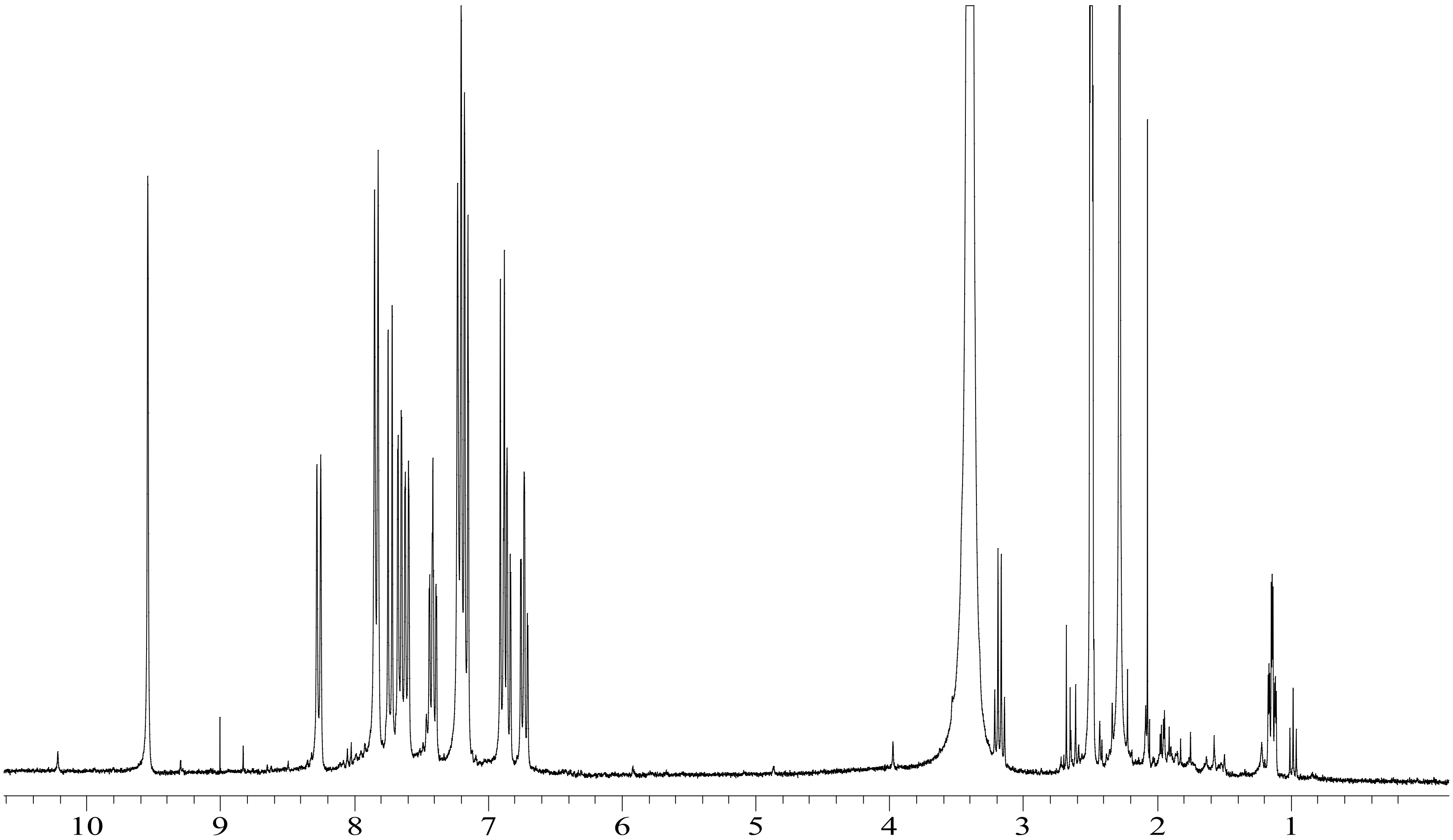

Figure **S45.** ^{1}H RMN spectrum of compound $[Zn_2L_2(CH_3OH)_2]$

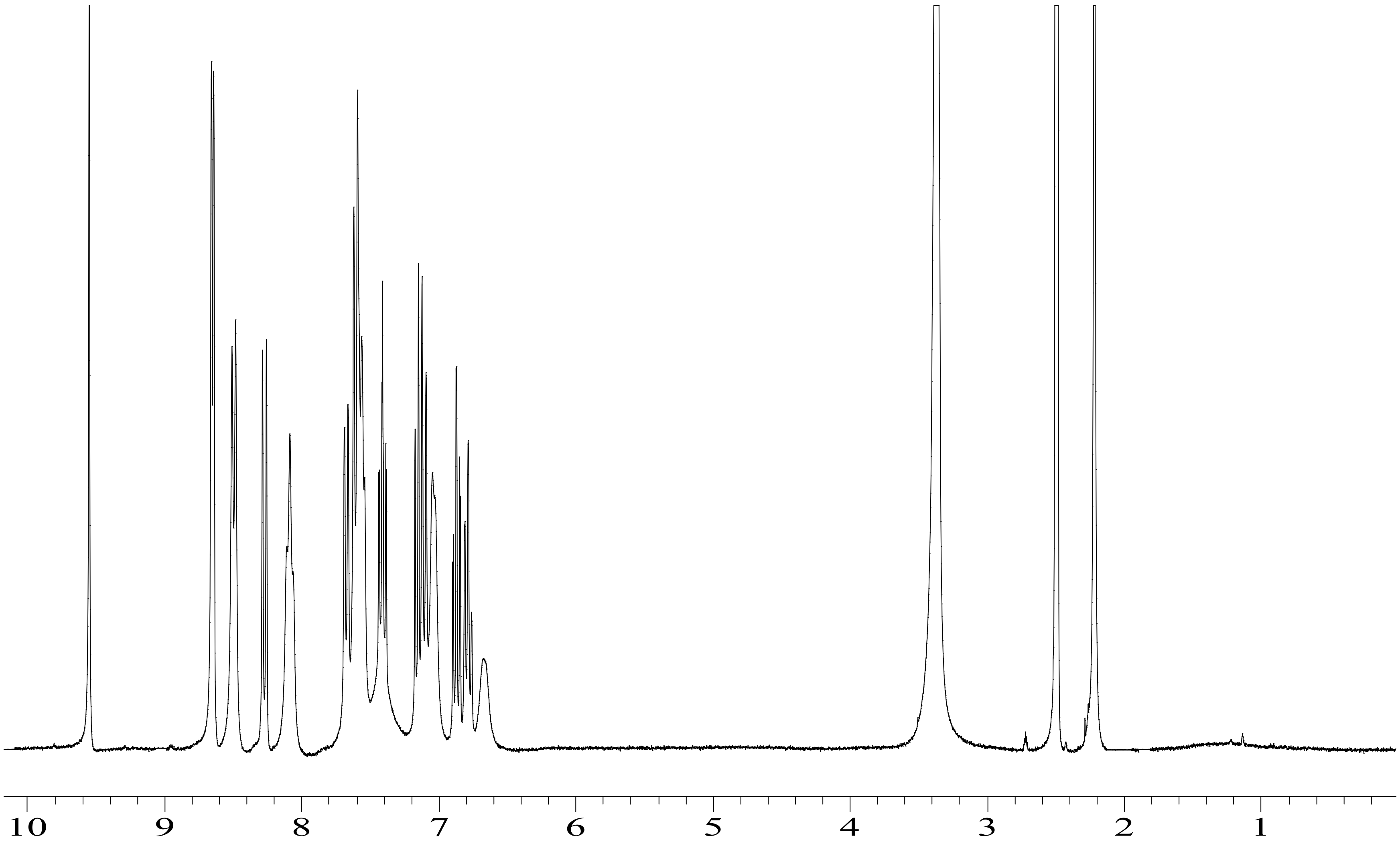

Figure **S46.** ^{1}H RMN spectrum of compound [ZnL(2,2′-bipy)]

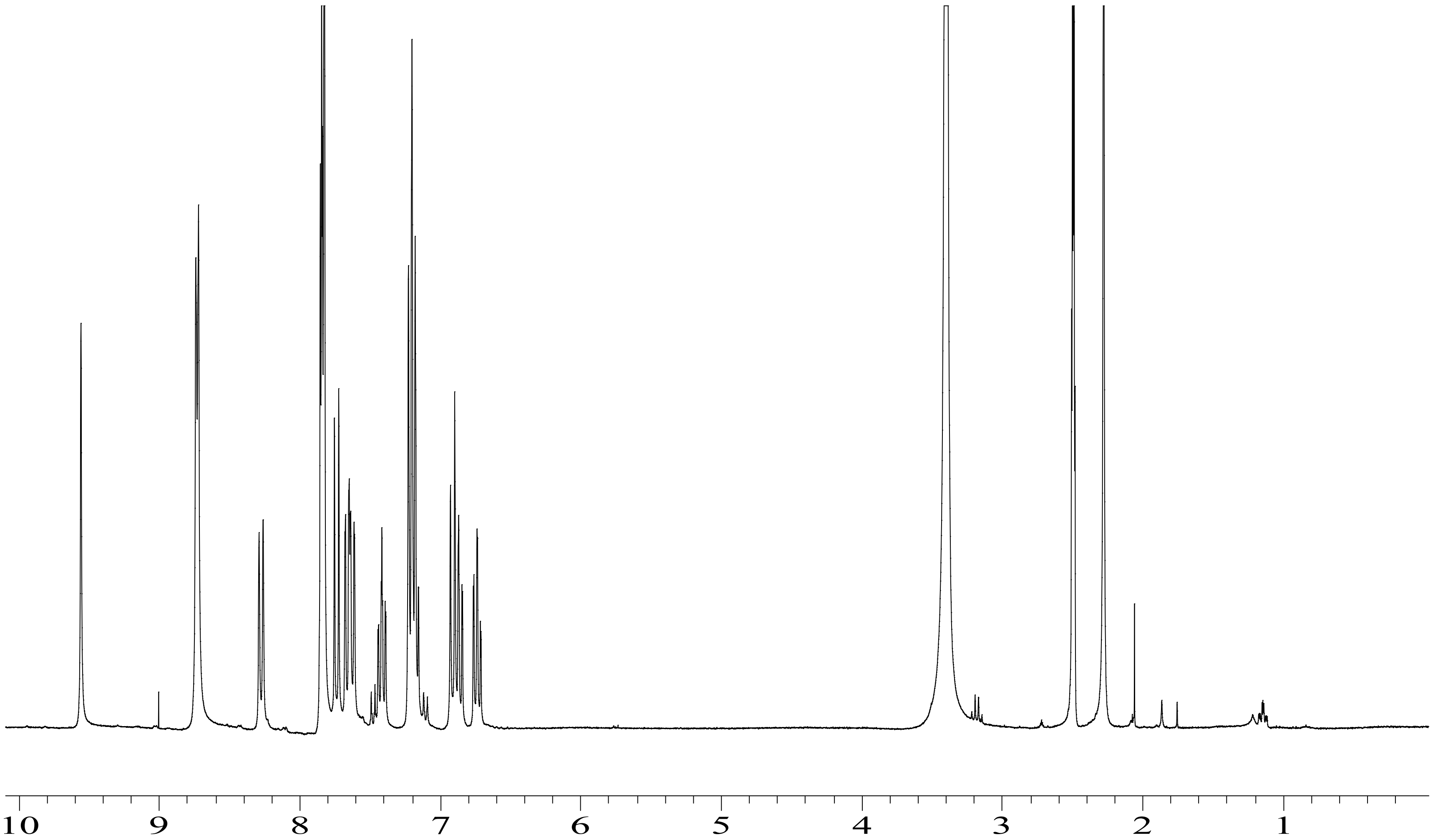

Figure S47. ^{1}H RMN spectrum of compound [(Zn$_2$L$_2$(4,4′-bipy)]

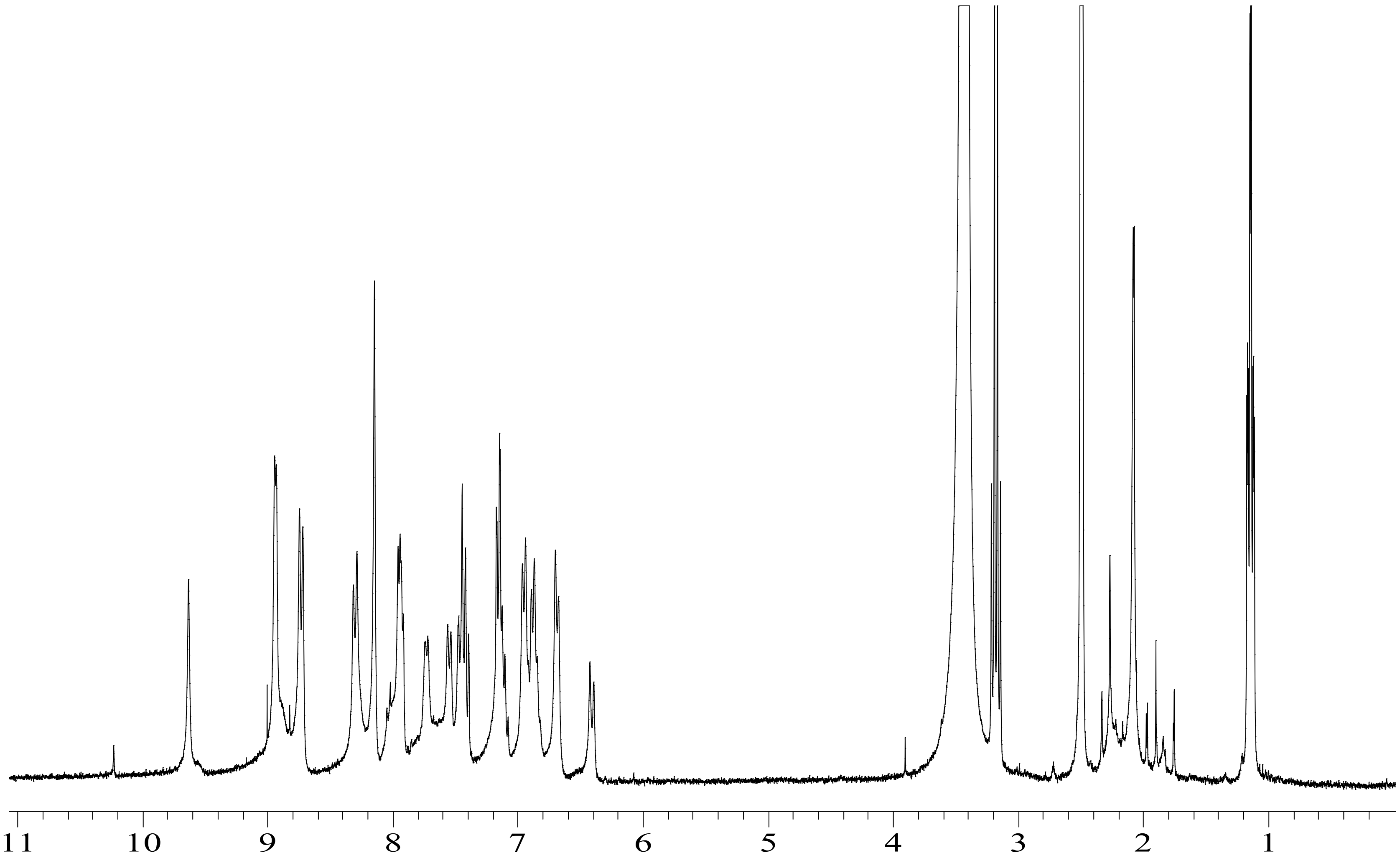

Figure S48. ^{1}H RMN spectrum of compound [ZnL(phen)]

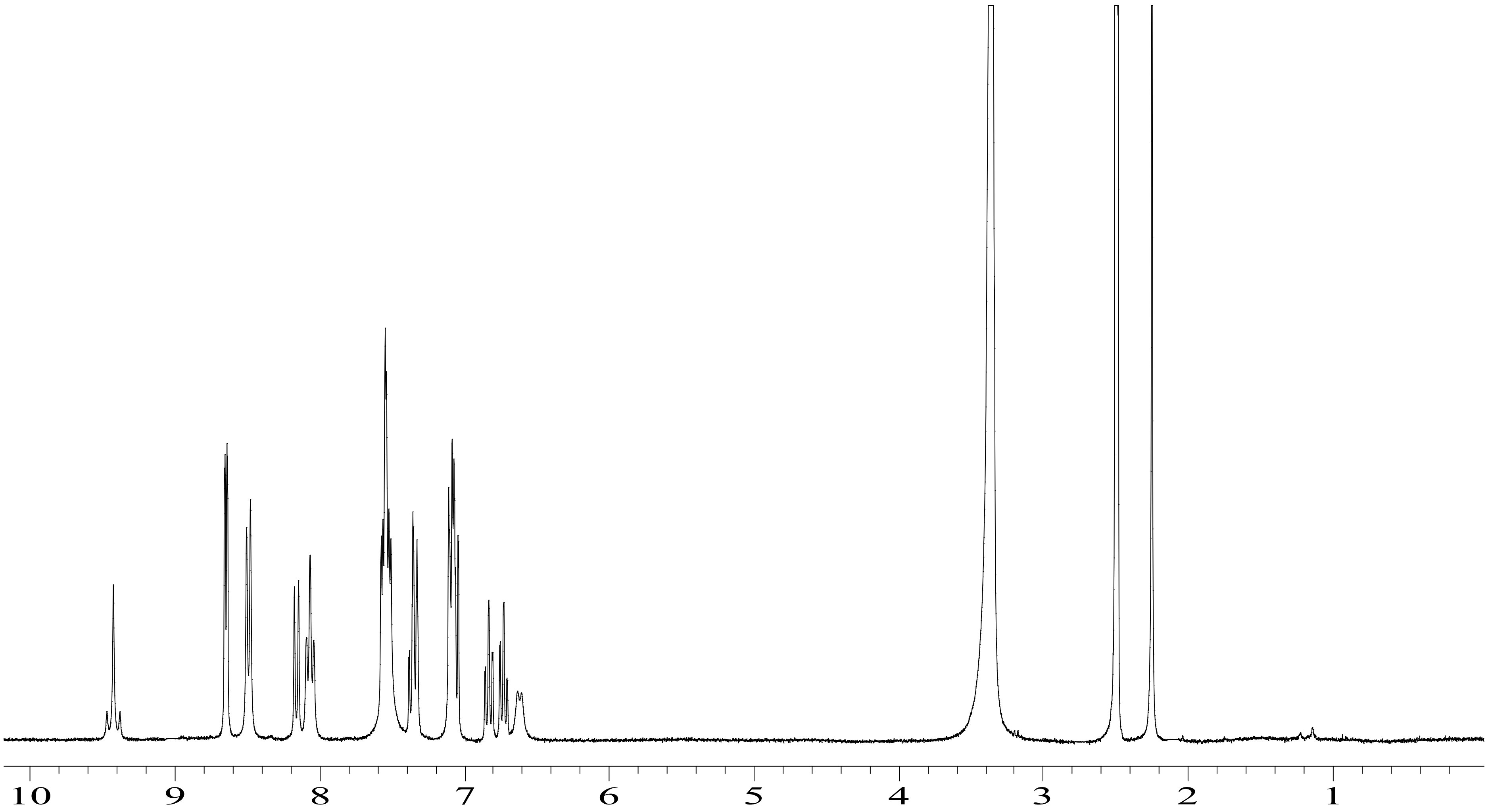

Figure **S49.** ¹H RMN spectrum of compound [CdL(2,2′-bipy)]

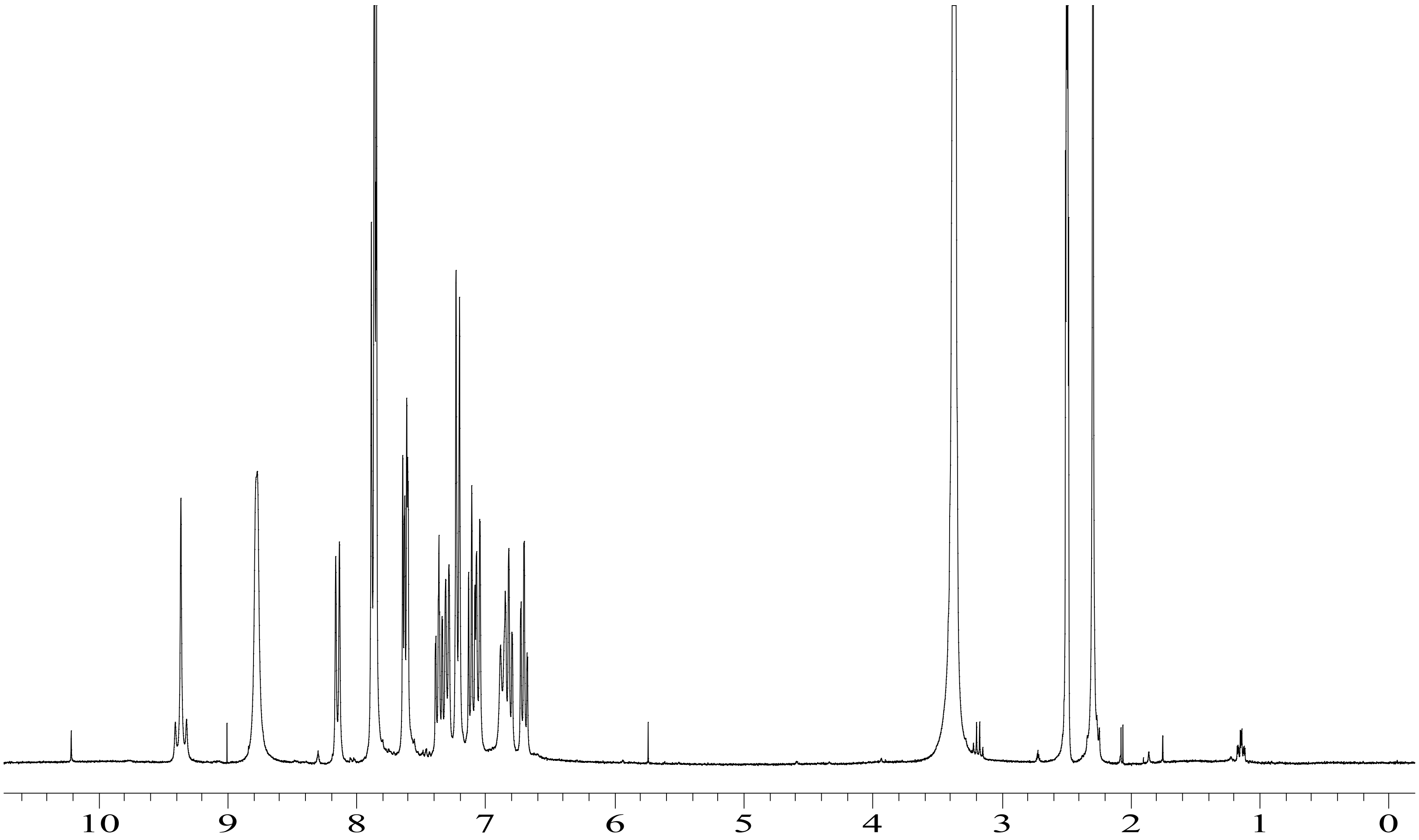

Figure **S50.** ^{1}H RMN spectrum of compound [Cd_2L_2(4,4′-bipy)]

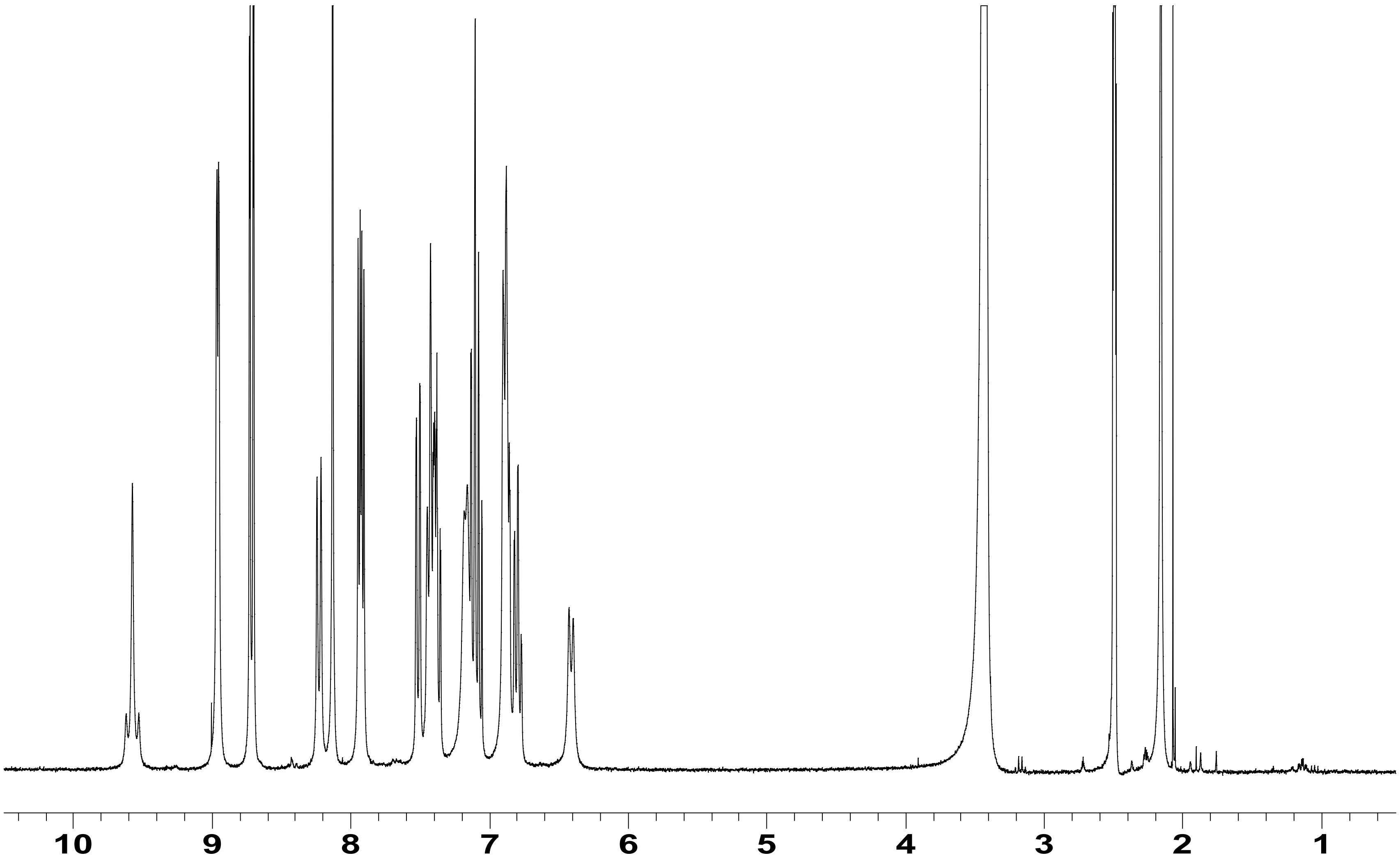

Figure **S51.** ^{1}H RMN spectrum of compound [CdL(phen)]
